# Comparative life-cycle analyses reveal interacting climatic and biotic drivers of population responses to climate change

**DOI:** 10.1093/pnasnexus/pgaf286

**Published:** 2025-09-05

**Authors:** Esin Ickin, Eva Conquet, Briana Abrahms, Steve D Albon, Daniel T Blumstein, Monica L Bond, P Dee Boersma, Tyler J Clark-Wolf, Tim Clutton-Brock, Aldo Compagnoni, Tomáš Dostálek, Sanne M Evers, Claudia Fichtel, Marlène Gamelon, David García-Callejas, Michael Griesser, Brage B Hansen, Stéphanie Jenouvrier, Kurt Jerstad, Peter M Kappeler, Kate Layton-Matthews, Derek E Lee, Francisco Lloret, Maarten J J E Loonen, Anne-Kathleen Malchow, Marta B Manser, Julien G A Martin, Ana Morales-González, Zuzana Münzbergová, Chloé R Nater, Neville Pillay, Maud Quéroué, Ole W Røstad, Teresa Sánchez-Mejía, Carsten Schradin, Bernt-Erik Sæther, Arpat Ozgul, Maria Paniw

**Affiliations:** Department of Evolutionary Biology and Environmental Sciences, University of Zurich, Zurich 8057, Switzerland; Department of Evolutionary Biology and Environmental Sciences, University of Zurich, Zurich 8057, Switzerland; Department of Biology, Center for Ecosystem Sentinels, University of Washington, Seattle, WA 98195, USA; The James Hutton Institute, Craigiebuckler, Aberdeen AB15 8QH, United Kingdom; Department of Ecology and Evolutionary Biology, University of California Los Angeles, Los Angeles, CA 90095, USA; The Rocky Mountain Biological Laboratory, Crested Butte, CO 81224, USA; Department of Evolutionary Biology and Environmental Sciences, University of Zurich, Zurich 8057, Switzerland; Wild Nature Institute, Concord, NH 03301, USA; Department of Biology, Center for Ecosystem Sentinels, University of Washington, Seattle, WA 98195, USA; Department of Biology, Center for Ecosystem Sentinels, University of Washington, Seattle, WA 98195, USA; Department of Wildland Resources and Ecology Center, Utah State University, Logan, UT 84322, USA; Department of Zoology, University of Cambridge, Downing St 1, Cambridge CB2 3EJ, United Kingdom; Kalahari Research Trust, Kuruman River Reserve, Kuruman, Northern Cape 8460, South Africa; Mammal Research Institute, University of Pretoria, Hatfield 0028, South Africa; Martin Luther University Halle-Wittenberg, Halle (Saale) 06108, Germany; German Centre for Integrative Biodiversity Research (IDiv), Leipzig 04103, Germany; Department of Population Ecology, Institute of Botany, Czech Academy of Sciences, Průhonice 25243, Czech Republic; Department of Botany, Faculty of Science, Charles University, Prague 12800, Czech Republic; German Centre for Integrative Biodiversity Research (IDiv), Leipzig 04103, Germany; Department of Conservation Biology and Global Change, Estación de Doñana (EBD-CSIC), Seville 41092, Spain; Department of Community Ecology, Helmholtz Centre for Environmental Research—UFZ, Halle (Saale) 06120, Germany; Behavioral Ecology and Sociobiology Unit, German Primate Center, Leibniz Institute for Primate Research, Göttingen 38510, Germany; Laboratoire de Biométrie et Biologie Evolutive, UMR 5558, CNRS, Université Claude Bernard Lyon 1, Villeurbanne 69622, France; Centre for Integrative Ecology, School of Biological Sciences, University of Canterbury, Private Bag 4800, Christchurch 8140, Aotearoa New Zealand; Manaaki Whenua-Landcare Research, PO Box 69040, Lincoln 7640, Aotearoa New Zealand; Center for the Advanced Study of Collective Behavior, University of Konstanz, Konstanz 78457, Germany; Department of Collective Behaviour, Max Planck Institute of Animal Behavior, Konstanz 78457, Germany; Gjærevoll Centre for Biodiversity Foresight Analyses, Norwegian University of Science and Technology (NTNU), Trondheim 7034, Norway; Department of Terrestrial Ecology, Norwegian Institute of Nature Research (NINA), Trondheim 7034, Norway; Biology Department, Woods Hole Oceanographic Institution, Woods Hole, MA 02543-1050, USA; Jerstad Viltforvaltning, Mandal 4514, Norway; Behavioral Ecology and Sociobiology Unit, German Primate Center, Leibniz Institute for Primate Research, Göttingen 38510, Germany; Department of Sociobiology/Anthropology, Johann-Friedrich-Blumenbach Institute of Zoology and Anthropology, University of Göttingen, Göttingen 37073, Germany; Department Oslo, Norwegian Institute for Nature Research, Trondheim 7034, Norway; Wild Nature Institute, Concord, NH 03301, USA; Center for Ecological Research and Forestry Applications (CREAF), Cerdanyola del Vallès 08193, Spain; Department Animal Biology, Plant Biology and Ecology, Universitat Autònoma Barcelona, Cerdanyola del Vallès 08193, Spain; Arctic Centre, Faculty of Arts, University of Groningen, PO Box 716, Groningen NL-9700 AS, The Netherlands; Theoretical Ecology, Universität Regensburg, Regensburg 93053, Germany; Department of Evolutionary Biology and Environmental Sciences, University of Zurich, Zurich 8057, Switzerland; Kalahari Research Trust, Kuruman River Reserve, Kuruman, Northern Cape 8460, South Africa; Mammal Research Institute, University of Pretoria, Hatfield 0028, South Africa; Department of Biology, University of Ottawa, ON, Canada K1N 9A4; Department of Conservation Biology and Global Change, Estación de Doñana (EBD-CSIC), Seville 41092, Spain; Department of Population Ecology, Institute of Botany, Czech Academy of Sciences, Průhonice 25243, Czech Republic; Department of Botany, Faculty of Science, Charles University, Prague 12800, Czech Republic; Department of Terrestrial Biodiversity, Norwegian Institute for Nature Research, Trondheim 7034, Norway; School of Animal, Plant and Environmental Sciences, University of the Witwatersrand, Johannesburg 2000, South Africa; CEFE, University of Montpellier, CNRS, EPHE, IRD, Montpellier 34090, France; Department of Ecology and Natural Resource Management, Norwegian University of Life Sciences, Ås 1432, Norway; Center for Ecological Research and Forestry Applications (CREAF), Cerdanyola del Vallès 08193, Spain; Department Animal Biology, Plant Biology and Ecology, Universitat Autònoma Barcelona, Cerdanyola del Vallès 08193, Spain; School of Animal, Plant and Environmental Sciences, University of the Witwatersrand, Johannesburg 2000, South Africa; Université de Strasbourg, CNRS, IPHC UMR 7178, Strasbourg F-67000, France; Department of Biology, Centre for Biodiversity Dynamics, Norwegian University of Science and Technology (NTNU), Trondheim 7034, Norway; Department of Evolutionary Biology and Environmental Sciences, University of Zurich, Zurich 8057, Switzerland; Department of Conservation Biology and Global Change, Estación de Doñana (EBD-CSIC), Seville 41092, Spain

**Keywords:** density dependence, comparative demography, structured population models, ecological forecasting, biodiversity conservation

## Abstract

Responses of natural populations to climate change are driven by how multiple climatic and biotic factors affect survival and reproduction, and ultimately shape population dynamics. Yet, despite substantial progress in synthesizing the sensitivity of populations to climatic variation, comparative studies still overlook such complex interactions among drivers that generate variation in population-level metrics. Here, we use a common framework to synthesize how the joint effects of climate and biotic drivers on different vital rates impact population change, using unique long-term data from 41 species, ranging from trees to primates. We show that simultaneous effects of multiple climatic drivers exacerbate population responses to climate change, especially for fast-lived species. However, accounting for density feedbacks under climate variation buffers the effects of climate change on population dynamics. In all species considered in our analyses, such interactions between climate and density had starkly different effects depending on the age, size, or life-cycle stage of individuals, regardless of the life history of species. Our work provides the first general framework to assess how covarying effects of climate and density across a wide range of population models can impact populations of plants and animals under climate change.

Significance Statement There is a growing consensus that complex interactions among vital rates and numerous abiotic and biotic drivers complicate simple predictions of climate-change impacts on plant and animal populations. Here, we use a unique dataset of some of the longest-studied populations of 41 plant, bird, and mammal species to compare the effects of such complex mechanisms on population persistence. Despite the unique context of each study population, our results show remarkable generalizable patterns of population responses to climate variation. To advance future research, we provide fully reproducible models and an open-access data repository, enabling broad-scale integration of demographic responses to climate change.

## Introduction

Among the multiple challenges for biodiversity conservation, the increasing severity of climate change, interacting with other global-change drivers, is of particular concern (1). Inferring general patterns of how populations of plants and animals respond to such complex interactions, beyond single case studies, is a priority for theoretical and applied research and management (2). All populations in natural communities are structured by variation in genetic and phenotypic traits, and often also developmental stages, which determine how different rates of survival and reproduction are spread throughout the life cycle (3). In structured populations, climatic effects on population abundances are then filtered by how different biotic and abiotic drivers (including climate) affect trait-, age-, or stage-specific survival and reproduction (4–13)). For instance, population persistence may be particularly affected when several climatic factors simultaneously reduce survival and reproduction of several life-cycle stages, accelerating population decline (5). In particular, compound effects of hotter and drier climatic conditions on individuals are projected to increase under climate change and can have strong negative impacts on natural populations and communities (14, 15), especially in combination with land-use change (16). However, populations may also be buffered from adverse climatic effect, when vital rates with higher impact on population growth, i.e. adult survival, exhibit the least temporal variability and thus stabilize population fitness (17–21). Furthermore, a decrease in one vital rate under climate stress (e.g. recruitment) can be compensated with increases in other vital rates, such as survival of the remaining recruits or adults, under negative density feedbacks (6, 7, 22). This occurs because, when individuals compete for resources, negative climatic effects on hetero- or conspecific abundance will also ease competition (6, 23), which can allow the populations to recover faster from or show higher resilience to adverse climatic effects (24). The role of density dependence may be particularly important in assessing climate-change effects on population dynamics (23). Therefore, to broadly understand the impacts of climate change in complex natural systems, we need to understand how intrinsic and interspecific mechanisms interact to mediate such impacts on natural populations (25, 26).

Despite substantial progress to synthesize the sensitivity of populations to climatic variation, comparative studies have largely overlooked complex mechanisms of interacting drivers and vital rates that generate variation in population-level metrics. For instance, previous studies have linked global indices of temperature and rainfall to abundances or population growth rates to show that terrestrial populations of plants and animals with shorter generation times are relatively more sensitive to climatic variation (27, 28). Despite producing important insights, such analyses have not investigated vital-rate responses to multiple climatic factors and did not consider biotic drivers such as density dependence. A recent study compared the relative effect on plant population growth rates of perturbing abiotic vs. biotic drivers, but did not assess how simultaneous effects of different drivers on different vital rates affect populations (29). This contrasts with the growing consensus that complex interactions among vital rates and biotic and climatic drivers complicate projections of persistence under climate change (25, 30–34).

We synthesize, for the first time, how interacting climatic and biotic drivers change population dynamics across taxa by affecting different vital rates such as reproduction and juvenile and adult survival. Given the evidence for the importance of the effects of multiple abiotic drivers and their interactions with density feedbacks on population dynamics (5–12), we hypothesized that, generally, the simultaneous effects of several climatic drivers in vital-rate models amplify population responses to climate change, but that climate-change impacts on populations are buffered when intra- or interspecific density dependence is incorporated in vital-rate models.

We reviewed the ecological literature and identified studies that quantitatively linked at least two climatic drivers or one climatic and one biotic driver to at least two vital rates. Following (31), we defined climatic drivers as direct measures of temperature or precipitation, i.e. not drivers that affected climate indirectly, such as the Southern Annular Mode (i.e. *Catharacta lönnbergi* from (35); see Supplementary material for a complete list of selection criteria). Among the biotic drivers, we distinguished intraspecific interactions (e.g. density dependence and social interactions) and interspecific interactions (e.g. competition, food availability, predation, and diseases). We then built structured population models and used them to compute sensitivities of population growth rates (36) to a given climatic driver, either accounting for simultaneous effects of all other drivers on vital rates or keeping other drivers fixed, thus reducing the complexity of environmental effects. We also compared the effects of perturbing different single vital rates to understand whether population-level sensitivities are driven by changes in specific vital rates across species. When testing our hypothesis, we controlled for potential confounding factors, most importantly the life-history strategy of populations, which has been shown to strongly mediate population responses to environmental change (27, 37). We created a database making all data and code freely available online to allow researchers to link age- or stage-specific vital rates to population responses under environmental change for further analyses such as forecasts.

## Results

We extracted data from 23 studies, including 41 species (15 birds, eight mammals, and 18 plant species). Among these species, 18 matrix population models, eight integral projection models, five integrated population models, and 10 individual-based models were used, and vital rates were typically modeled using generalized linear models. Among biotic drivers, intraspecific density dependence was most commonly included as a driver in vital-rate models (i.e. in 13 studies: four birds, six mammals, and three plants), while interspecific interactions were considered in only four cases. For an overview of life-history strategies, covariates, and demographic status of the species included in this comparative study, see Table S7. For each species, we calculated the scaled absolute sensitivities (|*S*|), i.e. changes in the population growth rate, *λ*, to observed climatic variation (standardized differences between maximum and minimum climatic values) (29). In most studies, we calculated *λ* for either a single (meta)population or a representative average population across the habitat range, as in the case of eight bird species (38) and 11 Mediterranean tree species (39)—that is, vital-rate models did not distinguish populations explicitly. However, three studies (see Supplementary material) modeled vital-rate responses to climatic and biotic drivers that differed among populations. Here, we averaged sensitivities across populations to calculate species-specific average sensitivities to climate comparable across species (29). Additional analyses showed that such averaging did not affect results (Table S4). We also repeated analyses excluding these three studies altogether; this did not affect our results either (Table S5).

**Fig. 1.**
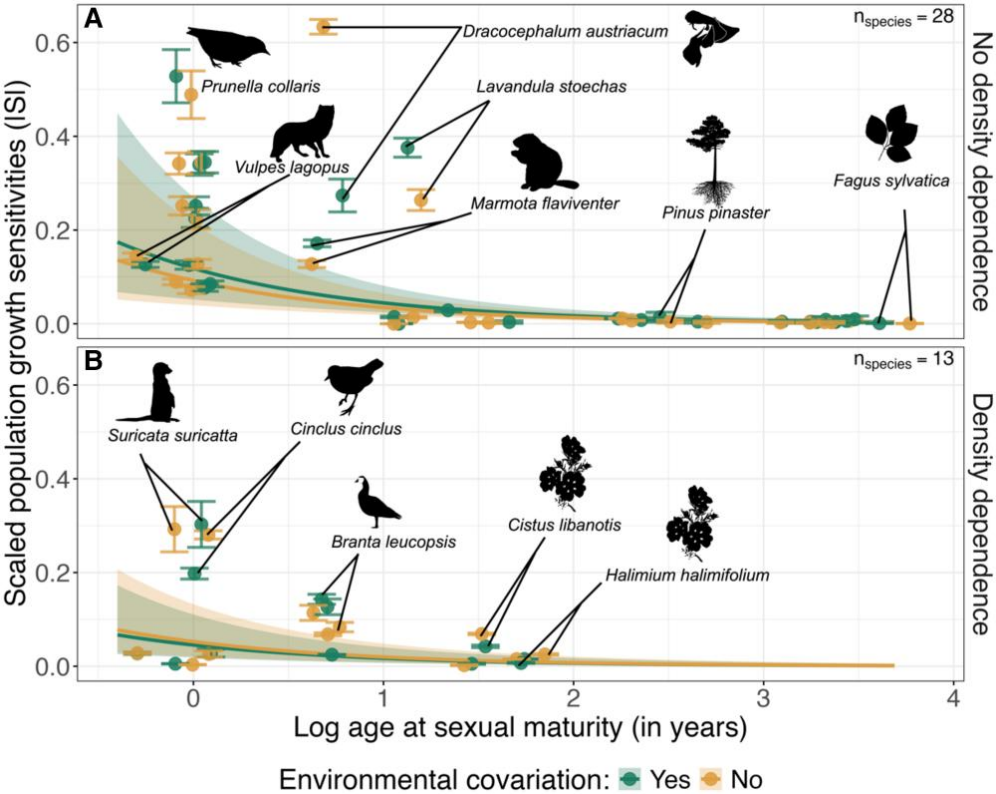
Scaled sensitivities of population growth rates to climate, |*S*|, are lower when accounting for changes in population density under climate change. Sensitivities are shown for species where density effects were not modeled explicitly (A) or were added (B) as covariates in vita-rate models. Different colors indicate sensitivity analyses under full environmental complexity (covariation with other drivers considered when perturbing a focal climate driver in vital-rate models) or reduced complexity (keeping other drivers as their average values when perturbing a focal driver). The lines represent predicted |*S*| over a range of ages of sexual maturity. The shaded areas indicate 95% model prediction intervals (see Table 1 for model coefficients). To aid visualization, the points show the observed sensitivity values of each species and perturbation scenario averaged over all perturbed climatic drivers and all resampled |*S*| under parameter uncertainty with error bars showing the SE. Figures S9–S11 show the full distributions of resampled values per species. We labeled some example species across different life histories and taxa. Note that the points for a given species on the *x* axis are slightly separated so that error bars do not overlap. Silhouettes were downloaded from PhyloPic, licence CC0 1.0, and CC BY-NC-SA 3.0 for *Dracocephalum austriacum* (credit: Alexander Schmidt-Lebuhn) and CC BY 4.0 for *Prunella collaris* (credit: Matej Frantisek Calfa).

We modeled the variation in |*S*| using a modified meta-regression approach (40), where we pooled the results from all studies into one generalized linear hierarchical model. Our model included average age at maturity, a proxy for the fast-slow continuum of life-history strategies (41). As expected, slower-paced species had lower absolute sensitivities of *λ* (|*S*|) to climatic drivers compared to faster-paced species (Fig. 1, Table 1; *β*_Maturity_ = −1.13 ± 0.19). These patterns agree with theoretical expectations (i.e. demographic buffering hypothesis (18, 42)) and previous empirical studies (27, 28, 37, 43) and suggest that fast-paced life histories across taxa are more labile to, or track, climatic fluctuations, whereas slow-paced life histories buffer population dynamics from multiple climatic effects (18, 27, 37).

**Table 1.** Output of model assessing how age at sexual maturity, covariation with other drivers, presence of density feedbacks in vital-rate models, and other covariates affected scaled sensitivities of population growth rates to changes in climate, |*S*|.

Fixed effects	Coefficient	SE	*P*-value
Intercept	−3.085	0.945	**0**.**001**
Covariation_no_	−0.250	0.112	**0**.**026**
Density_yes_	−1.004	0.556	0.070
Age at sexual maturity	−0.991	0.200	**<0**.**001**
Number of vital rates	−0.221	0.501	0.660
Parameters per vital rate	0.760	0.497	0.127
Covariation_no_:density_yes_	0.470	0.192	**0**.**014**

Random effects	Variance	SD	Prop. variance
Species/group (intercept)	1.738	1.318	0.633
Species/group covariation_no_	0.241	0.473	0.088
Group (intercept)	<0.001	<0.001	<0.01
Group covariation_no_	<0.001	<0.001	<0.01
Residual	0.767	0.757	0.279

Marginal *R*^2^ (variance explained by fixed effects): 0.300. Conditional *R*^2^ (variance explained by fixed and random effects): 0.829. The fixed effects and random effects of the GLMM with gamma log link are shown here. The coefficient, SE, and *P*-value are reported for each fixed effect, whereas variance and SD are reported for each random effect, as well as proportion of variance, which indicates the proportion of the total random-effect variance explained by different grouping variables. Nested random effects were incorporated due to multiple observations within species and groups (*n*_samples_ = 17,240, *n*_species_ = 41, *n*_groups_ = 3). *n*_samples_ reflects all resampled |*S*| for each perturbation scenario and species to account for parameter uncertainty. Bold *P*-values indicate statistical significance (*α* = 0.05).

### Population responses to multiple climatic drivers and density dependence

Across life histories, sensitivities |*S*| to changes in a focal climatic driver were consistently higher when covarying climatic drivers were also perturbed than when holding other climatic drivers constant (*β*_NoCovariation_ = −0.25 ± 0.11; Table 1, Fig. 1). Thus, synergistic effects of different climatic drivers can have a stronger impact on population dynamics than considering the effects of such drivers in isolation, as is typically done in sensitivity analyses. At the same time, |*S*| were lower for populations where intraspecific density dependence explicitly affected vital rates along with climatic drivers, as opposed to populations that did not consider how climatic drivers interact with density dependence (*β*_DensityYes_ = −1.00 ± 0.56; Table 1, Figs. 1 and S1). These differences in including vs. excluding density dependence in population models were strongest when we accounted for the full complexity of environmental effects in sensitivity analyses (Fig. S1). That is, |*S*| increased by holding density dependence constant when perturbing a climatic driver as opposed to adjusting for observed changes in intraspecific density when the focal perturbed climatic driver was at its minimum and maximum (*β*_NoCovariation:Density_ = 0.40 ± 0.19). This suggests that covariation between climate and density may be critical in moderating climate-change impacts on populations across a wide range of taxa (5–12, 44, 45). Additional analyses further isolating the effects of density feedbacks vs. different biotic and abiotic drivers in vital-rate models confirmed that covariation with density lowered |*S*| when climatic drivers were perturbed (Fig. S2).

### Demographic pathways of climate effects on populations

**Fig. 2.**
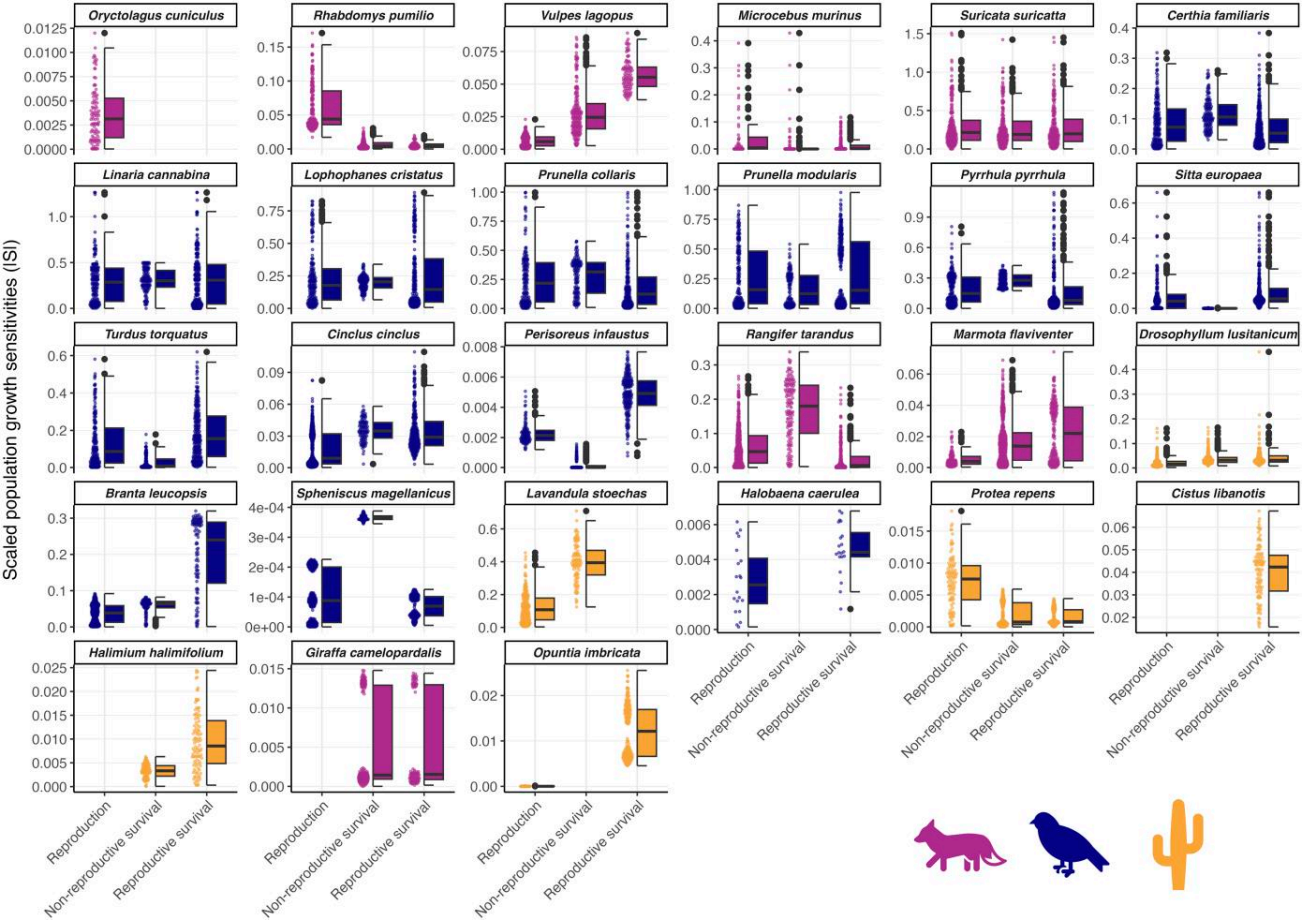
For any species, scaled sensitivities of population growth rates (|*S*|) vary substantially when perturbing single vital rates. Perturbations are shown for the species where we could perturb single vital rates. The plots are ordered by ascending age at sexual maturity and the colors indicate the taxa mammals, birds, and plants. The points represent |*S*| for each species, driver, vital rate, and parameter sample in vital-rate models. The boxplots display the distribution of |*S*|, including the median (central line), the interquartile range (box), and the range of the data (whiskers), with outliers shown as black points (*n*_samples per species and vital rate_ = 100, *n*_sample for *Halobaena caerulea* per vital rate_ = 50; see Supplementary material). If some sensitivities of some vital rates are missing, it is because these species did not have a climatic variable (but could have a biotic variable) in this specific vital rate.

We perturbed climatic drivers in each vital-rate model separately for 26 species to understand how different vital rates mediate the sensitivity of *λ* (|*S*|) to these drivers. For the remaining species, we could not perturb single vital rates due to the complexity of the models. A generalized linear regression model revealed that fast-paced life histories, i.e. ones with a lower age at maturity (43), were relatively more sensitive to climate perturbations in reproduction and survival of nonreproductive individuals than slow-paced life histories (Table 2, Fig. S5). This is to be expected, as reproduction contributes relatively more to population dynamics of fast-paced species (37). Our results provide further evidence that fast-paced life histories buffer critical vital rates from climatic perturbations less than slow-paced ones (18–20, 37), because they have a higher energy budget that they can invest into growth, reproduction, or dispersal after perturbations (46). However, a closer look at sensitivities of *λ* to vital-rate-specific effects of climatic drivers revealed a complex picture (Fig. 2). Across life histories, *λ* can be equally affected by perturbations in several vital rates, and some vital rates showed strong responses to one environmental variable but weak responses to other variables (Figs. 2 and S9–S38).

**Table 2.** Output of model assessing how age at sexual maturity, vital-rate type, presence of density feedbacks in vital-rate models, and other covariates affected scaled sensitivities of population growth rates to changes in climate, |*S*|, calculated by perturbing individual vital rates.

Fixed effects	Coefficient	SE	*P*-value
Intercept	−3.324	1.143	**0**.**003**
Vital rate_nonreproductive survival_	−0.620	0.385	0.107
Vital rate_reproductive survival_	0.030	0.363	0.936
Age at sexual maturity	−2.157	0.529	**<0**.**001**
Number of vital rates	−0.738	0.564	0.191
Parameters per vital rate	0.850	0.541	0.117
Age at sex. mat.:vital rate_nonreproductive survival_	1.412	0.596	**0**.**012**
Age at sex. mat.:vital rate_reproductive survival_	1.097	0.491	**0**.**025**

Random effects	Variance	SD	Prop. variance
Species/group (intercept)	2.057	1.434	0.272
Species/group vital rate_nonreproductive survival_	2.336	1.528	0.283
Species/group vital rate_reproductive survival_	2.078	1.442	0.264
Group (intercept)	<0.001	<0.001	<0.01
Group vital rate_nonreproductive survival_	<0.001	<0.001	<0.01
Group vital rate_reproductive survival_	<0.001	<0.001	<0.01
Residual	0.957	0.998	0.180

Marginal *R*^2^ (variance explained by fixed effects): 0.271. Conditional *R*^2^ (variance explained by fixed and random effects): 0.878. The fixed effects and random effects of the GLMM with gamma log link are shown here. The coefficient, SE, and *P*-value are reported for each fixed effect, whereas variance and SD are reported for each random effect, as well as proportion of variance, which indicates the proportion of the total random-effect variance explained by different grouping variables. Nested random effects were incorporated due to multiple observations within species and groups (*n*_samples_ = 13,040, *n*_species_ = 26, *n*_groups_ = 3). *n*_samples_ reflects all resampled |*S*| for each perturbation scenario and species to account for parameter uncertainty. Bold *P*-values indicate statistical significance (*α* = 0.05). Note that while perturbing one vital rate at a time, we accounted for covariation with other factors in the focal rate but set the covariates in the other vital-rate models to their mean values.

Overall, our results showed that growth-rate sensitivities, |*S*|, varied substantially among species/studies (Tables 1 and 2). While the fixed and random effects in our generalized linear mixed models (GLMMs) jointly explained >80% of the variance in |*S*|, the proportion of variance attributed to random effects was always relatively higher (Tables S1–S5, Fig. S3). The effect of species explained >50% of the random variation in the model. We also note that while 20 studies included only one species, three modeled several species, and we could not completely separate species and study effect—attempting to do so resulted in overparameterized random effects. Although we accounted for potential variables that may have confounded our results, i.e. number of vital rates modeled and average number of parameters per vital rate, one reason for such high variance among species or studies may be the varying complexity among studies in model design or the specific climatic variable considered—complexity that we could not account for as independent covariates in our analysis. On the other hand, high variability in responses to environmental drivers among species has also been observed in recent studies (28, 31, 47, 48). Thus, while we can discern generalizable patterns in population responses to climatic perturbations, only the inclusion of a wider range of future studies can disentangle the complex sources of context-dependent variation in population dynamics.

## Discussion

Natural populations of plants and animals are increasingly affected by climate-change worldwide (49, 50). By identifying under what context populations are more susceptible to negative effects of climatic drivers, we can prioritize conservation efforts and develop targeted strategies to mitigate adverse effects. Our comparative analyses shed light on some common demographic pathways through which populations of plants, mammals, and birds respond to complex interactions of climatic and biotic drivers. We show that simultaneous effects of multiple climatic drivers increase population sensitivity to climate change, while interactions between density dependence and climate can effectively lower such sensitivity. Our results thus have important implications for assessing how resilient populations are to climate change. They suggest that, in cases in which we know that multiple climate drivers influence vital rates, measuring the effect of only one of these climatic drivers on population dynamics likely overestimates its effects, while omitting how climate interacts with density feedbacks can substantially underestimate indirect effects of climate on populations.

Recent studies have emphasized that future climate risks to natural populations and humans will be exacerbated by compound effects of climate drivers (1, 51). While previous research has focused on understanding such compound effects on single species or populations (e.g. reviewed in 28, 32, 52), our results provide the first comparative evidence across different contexts that synergistic effects of different climatic drivers can have a strong impact on population dynamics. Compound climatic effects, such as low rainfall and high temperature, often constitute climatic extremes, e.g. hot droughts (51) and are becoming increasingly common (1). Such extremes can have strong, nonadditive effects on physiological processes of plants (53) and animals (54), negatively affecting population dynamics (5, 30, 55). In meerkats (*Suricata suricatta*), for instance, extreme heat in a relatively dry rainy season can lead to substantial loss of body mass and increased risks of deadly disease outbreaks (56). We note, however, that our study assessed changes in the magnitude but not in the direction of population responses to perturbations in climate. Therefore, compound effects, such as unusually warm and rainy reproductive seasons, may also lead to strong increases in population growth (56), particularly for fast life histories (42, 57).

Climatic factors do not affect populations in isolation; other abiotic and biotic factors also play a role, and their impacts vary among populations and individuals within those populations (32, 58). Our results suggest that across taxa, adverse climate effects can be buffered by decreasing the number of individuals in a population and thus easing the effects of intraspecific density, when present in populations (5, 7). In turn, for populations that increase in abundance under climate change, a resulting stronger effect of negative density dependence may increase population fluctuations under adverse environmental conditions (34). Other studies have also demonstrated the importance of density feedbacks in regulating population responses under land-use change (59) or disease outbreaks (60, 61), while populations of some social species that show nonlinear responses to population densities may be particularly susceptible to climate change if adverse climatic effects reduce optimal densities (5). Similarly, climate change also affects populations through changes in interspecific interactions such as predation, competition, or facilitation (12, 62, 63). However, interspecific interactions are still very rarely explicitly modeled when projecting population dynamics (31), including in the studies used in our meta-analysis.

Despite this growing evidence on the importance of assessing interactions of abiotic and biotic effects when quantifying population persistence under climate change (4, 5, 13, 29, 31), such assessments are challenging. Unlike climatic variables that are often included as continuous covariates in vital-rate models and are easily perturbed, interactions with individuals of the same population or even different species took on many complex forms in the population models we used in this study. Some studies only included indirect or static measures of biotic effects. For example, the tree species in our analysis had a colonization factor in their models, which was indirectly related to density but was decoupled from climate variables in vital rates (39). Similarly, the models of *Certhia familiaris*, *Linaria cannabina*, *Lophophanes cristatus*, *Prunella collaris*, *Prunella modularis*, *Pyrrhula pyrrhula*, *Sitta europaea*, and *Turdus torquatus* did not contain density as a continuous driver in their vital-rate models (which was required for our sensitivity analyses), but density served as a fixed species-specific parameter affecting fecundity (38). Thus, we could only assess the effects of covariation between climate and density dependence in 13 of the 41 modeled species. Although they represented all three taxonomic groups and covered a wide range of life histories, resulting in an unbiased sample, understanding whether density feedbacks are a general mechanism that moderates population fluctuations under climate change for a wider range of taxa requires broadening comparative analyses that can account for complex density effects.

Density feedbacks are not equally important in all populations (64), and their effects have been tested and considered to not substantially affect population dynamics in the case of *Marmota flaviventer* and *Lavandula stoechas* (see Supplementary material). However, the potential effects of density feedbacks have not been tested in many recent population model (31), likely due to a combination of lack of data and model complexity. In addition, most frameworks to predict biodiversity loss under global change do not explicitly model dynamic interactions between density and global-change drivers (65). We thus emphasize that including density feedbacks in the climate-demography models, for instance using population density or population size as a covariate in models (12, 34), may be key to understand how resilient natural populations are to climate change. If such feedback is not included due to data limitations or modeling constraints, our results suggest that it is important to at least discuss the potential implications of such omissions (66).

Ultimately, the effects of climate change on population dynamics are filtered by the strength and direction of driver effects on different vital rates and how much the latter contribute to population dynamics (4–13, 19, 23, 30, 33–35, 37). For any life history, even slow-paced ones where adult survival is the key vital rate driving population dynamics (37), changes in population growth were the results of complex effects of various drivers across different vital rates, showing high context dependence (13). Rainfall scarcity or extreme temperatures may differently affect individuals depending on the habitat, season, and life-cycle stage considered (5, 30), or depending on how other species in a given community are responding to climate change (62). The complexity of the life cycle may also indicate how much a population is buffered from adverse environmental effects (52). Some species have dormant life-cycle stages that can protect populations from environmental fluctuations (62). Dispersal, which was modeled in some studies considered here (see Supplementary material), can stabilize decreasing populations and allow individuals to track new suitable habitats and may itself be strongly mediated by climate (67). Therefore, from trees to primates, identifying how different abiotic and biotic factors impact populations across their full life cycle is a key to be able to target conservation efforts towards certain factors during certain times of the life cycle.

Our work has advanced comparative demographic analyses in two important ways. First, we standardized sensitivity analyses across a wide variety of population models, ranging from classic matrix population models to integrated population and integral projection models and individual-based models. By including the experts for each study system, we ensured that our methods did not produce inadvertent errors. Second, we provide a freely accessible and dynamic (i.e. constantly updated) database of population models that was compiled for this study. This offers an ideal basis to expand the number of studies and analyses in the future—for instance, forecasting how changes of local climatic drivers may affect populations and whether such effects can be approximated by global climate indices (68). We also recognize several limitations of our work. One limitation is that we could not account for taxonomic and geographical biases, as we relied on available high-quality structured models that integrate multiple environmental factors (see Supplementary material for study-specific details). Such tailored models are available for specific terrestrial plants, mammals, and birds but are still lacking for many invertebrate species (69, 70), where relatively little is known on the demographic pathways through which climate-change impacts abundance (71). We also have a geographic bias in our data, as most study systems are from the Northern Hemisphere. Additionally, we only considered studies published in English. These types of biases can limit our ability to generalize patterns and employ conservation efforts based to comparative analyses (72, 73).

When searching the literature for appropriate studies, we also discovered that reproducibility of ecological studies remains a problem. Of the 76 studies that met our search criteria, we could only replicate population models of 24%. For the remaining studies, data and code to replicate analyses were not freely available and could often not be reproduced even when in contact with authors. Thus, we emphasize that making not just data but also code available is an important step towards reproducible comparative analyses in ecology (74).

Our comparative analyses provide evidence that interactions among biotic and abiotic drivers, and the complex effects of such multiple drivers on different vital rates, hinder simplistic predictions of population persistence under climate change. We emphasize the need to recognize and incorporate interactions between climate and density dependence into full life-cycle models in order to understand and potentially mitigate the threat that climate-change poses on natural populations.

## Materials and methods

### Literature search

Our main objective was to collect code and data from studies which (i) modeled vital rates (e.g. survival, growth, and reproduction) in natural populations as a function of at least two climatic variables or one climatic and one biotic variable and (ii) constructed structured population models from which population growth rates could be obtained. We focused on studies where data were obtained in natural, unmanipulated populations (i.e. discarding experimental studies); and where the environmental variables were continuous so that we could calculate means and SEs (Eq. 1). We therefore excluded studies that constructed models for good/bad, dry/wet environments, etc. To obtain suitable studies, we performed a targeted review of the literature. We first considered a recent review, which revealed a lack of understanding regarding comprehensive demographic responses to climate change for terrestrial mammals, including 87 species (31). From the publications in this review, we selected those that met our criteria. To supplement data from this list of studies, we conducted a Web of Science search using the search terms from (31) and also checked the Padrino database (75) as well as (76) (details in Supplementary material). To be included in our database, vital-rate models had to be reproducible, i.e. the regression models were fully reported, including their formula, coefficients, and SEs. We were able to obtain data from 23 studies that met all these criteria.

As the first step of the analysis, we prepared a standardized protocol to build and perturb different structured population models to maximize the ease of comparison across studies (https://doi.org/10.5281/zenodo.16992231). For help with conducting these analyses for the selected models, we contacted the authors of relevant studies. We extracted regression coefficients from tables to rebuild vital-rate models when possible; alternatively, the latter were provided by the authors of a given study. We then reconstructed population models from these vital rates, and the authors from the original papers reviewed these models to ensure that they were correct. In some cases, authors already provided the R code to rebuild the population model (for more information, see Supplementary material). The environmental covariate data were also obtained from the authors of the papers. All studies built structured population models based on >7 years of demographic data collection and/or using data across the distribution range of species, and the range of environmental covariate values was sufficient to robustly build and perturb structured population models (see Supplementary material on study-specific details).

Next, we compared among the species how perturbations in climatic variables affect long-term population fitness, *λ*, i.e. the sensitivity of *λ* to climatic drivers. For studies that provided matrix population models or integral projection models, we calculated *λ* as the annual asymptotic population growth rate using the R package popbio (77) version 2.7. For studies that developed individual-based or integrated models, we calculated *λ* as the mean of annual growth rates over at least 50 years from at least 100 simulations (see Supplementary material for study-specific details; Figs. S38–S52). The approach of how *λ* was calculated did not affect our results (Table S3, Fig. S6). To obtain sensitivities of *λ* to climatic drivers, we calculated *λ* under minimum and maximum values of a climatic driver while (i) accounting for the actual observed values of other drivers when the focal driver was at its minimum or maximum (sensitivities with “covariation”) or (ii) holding the other drivers constant at their average values (sensitivities “without covariation”). When studies modeled random year effects consistently across vital rates, we set the years to ones where a climatic driver was at its minimum or maximum in analyses. We then calculated the scaled sensitivities according to Morris et al. (29) for each population and driver (Eq. 1):


(1) $$|S|=\left|\frac{\lambda_{\max}-\lambda_{\min}}{(d_{\max}-d_{\min})/SD_{d}}\right|.$$

The driver values *d*_max_ and *d*_min_ produced the population growth rates when the driver was set to its maximum value (*λ*_max_) and its minimum value (*λ*_min_). The denominator of the scaled sensitivity |*S*| is the difference in the driver levels in SD units. The *scaled* sensitivity makes it possible to compare |*S*| across different studies and driver types (29). We calculated |*S*| for each climatic driver in vital-rate models (see “Sensitivity analyses” in Supplementary material). We tested the robustness of the sensitivity metric by comparing |*S*| to the most common type of metric for summarizing outcomes in ecological meta-analyses: log response ratios (see “Alternative sensitivity parameterizations” in Supplementary material, Figs. S7 and S8, Table S6).

We accounted for uncertainties around all |*S*| estimates by resampling parameters from vital-rate models and recalculating *λ* and |*S*| each time. More specifically, if a study reported the SEs of the regression coefficients, we simulated the parameter distributions and sampled parameters from it, whereas in the case of Bayesian regressions, we sampled parameters from the Markov Chain Monte Carlo (MCMC) posteriors. We produced 100 |*S*| estimates for most species but had to use fewer samples in some cases due to computational limits (see species-specific details in Supplementary material). In three cases, we averaged |*S*| over different populations to get species-specific results. However, this averaging did not affect our overall conclusions (Table S4).

Further, we perturbed the climatic drivers in each vital rate separately whenever possible (Figs. S12–S38 for the specific vital rates in each species’ model), in the same manner as above, to get vital-rate-specific |*S*|. In this case, all environmental driver values covaried with the focal driver in the perturbed vital rate but were held at their average values in other vital rates. Lastly, for populations (*n* = 13) where intraspecific density dependence was explicitly considered as a driver in vital-rate models, we performed additional perturbations: We accounted for the actual observed values of other climatic or biotic drivers when perturbing a focal climatic driver (sensitivities with covariation), but held densities constant (i.e. did not account for covariation with density). We did this to test how much |*S*| depended on density dependence moderating the effects of climatic changes.

### Statistical analyses

We used a GLMM, assuming a Gamma-distributed response under a log link function, to understand the underlying mechanisms influencing population-level sensitivities |*S*| to climate change. We chose the Gamma distribution because the scaled sensitivities were positive values larger than zero. The resulting model fit well to observed data (Fig. 1), and model fit was substantially better than using a log-normal distribution, based on Akaike information criterion (AIC) and residual plots (78). We included log(age at sexual maturity) as a continuous covariate for the effect of life-history speed on |*S*|. To test whether covariation among climatic drivers and lambda changed |*S*|, we incorporated as predictor variables covariation with other drivers when *λ* was calculated under minimum/maximum values of a focal climatic driver (categorical; accounted for or not), intraspecific density effects (categorical; incorporated or not in vital-rate models), and the interaction between the two. We focused on intraspecific density effects to analyze the role of biotic interactions in population dynamics because this was the most common type of biotic variable included in vital rate models across species (Table S7). We also controlled for a potential effect of model complexity on |*S*|, by including the log(number of vital rates) and log(mean parameters per vital rate) in each population model. Taxonomic groups and species were integrated as nested random effects on the model intercept to account for nonindependent species-specific perturbations of different climatic drivers in vital-rate models. To account for differences among taxonomic groups and species in how much driver covariation affects |*S*|, the same nested random effects were also applied on the slope of the covariation variable. We also assessed whether |*S*| differed depending on which type of climatic driver was perturbed in vital-rate models (temperature vs. rainfall) by fitting another GLMM akin to the main analysis but including climatic driver as a covariate (Table S2, Fig. S4).

To better understand which vital rates were driving |*S*|, we repeated the GLMMs using |*S*| calculated by perturbing climatic drivers in single vital rates. To facilitate comparisons among species, we grouped the vital rates of each species into three main types: survival of nonreproductive individuals (including juveniles), survival of reproductive individuals, and reproduction (including reproductive success and recruitment). We excluded trait change (including growth and maturation) as a vital rate, as it was only modeled in four species: *M. flaviventer*, *Rhabdomys pumilio*, *Suricata suricatta*, and *Protea repens*. The resulting GLMM had a similar structure as the one for the global |*S*|, with two differences. First, as we calculated vital-rate-specific |*S*| without simplifying driver covariation in specific vital rates, covariation was not included in the model. Second, as we held variables constant in nonperturbed vital rates, we simplified the model structure further by excluding whether species included or excluded density feedbacks in vital-rate and population models. We included main vital-rate type as a covariate and tested whether the climatic effects of different vital rates on |*S*| differed among life histories, via the effects of log(age at maturity), and used an interaction term of vital rate and age at sexual maturity.

We calculated marginal and conditional *R*^2^ for all GLMMs to quantify the variance in the data explained by the fixed effects and random and fixed effects, respectively (79). We made all the data and code available online, along with the templates, ensuring that future analyses follow the same structure (https://doi.org/10.5281/zenodo.16992231).

## Supplementary Material

pgaf286_Supplementary_Data

## Data Availability

All data and code have been archived at Zenodo (https://doi.org/10.5281/zenodo.16992231). All analyses are fully reproducible.

## Preprints

This manuscript was posted on a preprint at https://doi.org/10.32942/X24C92.

## References

[1] Zscheischler J, et al 2018. Future climate risk from compound events. Nature Clim Change. 8:469–477.

[2] Leclère D, et al 2020. Bending the curve of terrestrial biodiversity needs an integrated strategy. Nature. 585:551–556. 32908312 10.1038/s41586-020-2705-y

[3] Ebenman B, Persson L. Size-structured populations: ecology and evolution. Springer Science & Business Media, 2012.

[4] Coulson T, et al 2001. Age, sex, density, winter weather, and population crashes in soay sheep. Science. 292:1528–1531. 11375487 10.1126/science.292.5521.1528

[5] Paniw M, Maag N, Cozzi G, Clutton-Brock T, Ozgul A. 2019. Life history responses of meerkats to seasonal changes in extreme environments. Science. 363:631–635. 30733418 10.1126/science.aau5905

[6] Reed TE, Grøtan V, Jenouvrier S, Sæther B-E, Visser ME. 2013. Population growth in a wild bird is buffered against phenological mismatch. Science. 340:488–491. 23620055 10.1126/science.1232870

[7] Hansen BB, et al 2019. More frequent extreme climate events stabilize reindeer population dynamics. Nat Commun. 10:1616. 30962419 10.1038/s41467-019-09332-5 PMC6453938

[8] Lima M, Stenseth NC, Jaksic FM. 2002. Population dynamics of a South American rodent: seasonal structure interacting with climate, density dependence and predator effects. Proc Biol Sci. 269:2579–2586. 12573073 10.1098/rspb.2002.2142 PMC1691189

[9] Barbraud C, Weimerskirch H. 2003. Climate and density shape population dynamics of a marine top predator. Proc R Soc Lond B Biol Sci. 270:2111–2116. 10.1098/rspb.2003.2488 PMC1691492 14561273

[10] Sanczuk P, et al 2023. Microclimate and forest density drive plant population dynamics under climate change. Nat Clim Chang. 13:840–847.

[11] Stenseth HC, et al 2003. Seasonality, density dependence, and population cycles in Hokkaido voles. Proc Natl Acad Sci U S A. 100:11478–11483. 14504382 10.1073/pnas.1935306100 PMC208783

[12] Nater CR, Van Benthem KJ, Canale CI, Schradin C, Ozgul A. 2018. Density feedbacks mediate effects of environmental change on population dynamics of a semidesert rodent. J Anim Ecol. 87:1534–1546. 30058150 10.1111/1365-2656.12888

[13] Jenouvrier S . 2013. Impacts of climate change on avian populations. Glob Change Biol. 19:2036–2057. 10.1111/gcb.12195 23505016

[14] Bourne AR, Cunningham SJ, Spottiswoode CN, Ridley AR. 2020. Hot droughts compromise interannual survival across all group sizes in a cooperatively breeding bird. Ecol Lett. 23:1776–1788. 32945068 10.1111/ele.13604

[15] Larsen TH . 2012. Upslope range shifts of Andean dung beetles in response to deforestation: compounding and confounding effects of microclimatic change. Biotropica. 44:82–89.

[16] Forister ML, et al 2010. Compounded effects of climate change and habitat alteration shift patterns of butterfly diversity. Proc Natl Acad Sci U S A. 107:2088–2092. 20133854 10.1073/pnas.0909686107 PMC2836664

[17] Stearns SC . The evolution of life histories. Oxford University Press, 1992.

[18] Hilde CH, et al 2020. The demographic buffering hypothesis: evidence and challenges. Trends Ecol Evol. 35:523–538. 32396819 10.1016/j.tree.2020.02.004

[19] Sæther B-E, Bakke Ø. 2000. Avian life history variation and contribution of demographic traits to the population growth rate. Ecology. 81:642–653.

[20] Gaillard J-M, Yoccoz NG. 2003. Temporal variation in survival of mammals: a case of environmental canalization? Ecology. 84:3294–3306.

[21] Le Coeur C, Yoccoz NG, Salguero-Gómez R, Vindenes Y. 2022. Life history adaptations to fluctuating environments: combined effects of demographic buffering and lability. Ecol Lett. 25:2107–2119. 35986627 10.1111/ele.14071 PMC9804727

[22] McDonald JL, et al 2017. Divergent demographic strategies of plants in variable environments. Nat Ecol Evol. 1:0029. 10.1038/s41559-016-0029 28812611

[23] Peeters B, et al 2022. Harvesting can stabilise population fluctuations and buffer the impacts of extreme climatic events. Ecol Lett. 25:863–875. 35103374 10.1111/ele.13963

[24] Conquet E, et al 2022. Demographic consequences of changes in environmental periodicity. Ecology. 104:e3894. 10.1002/ecy.3894 36208282

[25] Urban MC, et al 2016. Improving the forecast for biodiversity under climate change. Science. 353:aad8466. 27609898 10.1126/science.aad8466

[26] de Roos AM . 2021. Dynamic population stage structure due to juvenile–adult asymmetry stabilizes complex ecological communities. Proc Natl Acad Sci U S A. 118:e2023709118. 34021084 10.1073/pnas.2023709118 PMC8166188

[27] Compagnoni A, et al 2021. Herbaceous perennial plants with short generation time have stronger responses to climate anomalies than those with longer generation time. Nat Commun. 12:1824. 33758189 10.1038/s41467-021-21977-9 PMC7988175

[28] Jackson J, Coeur CL, Jones O. 2022. Life history predicts global population responses to the weather in terrestrial mammals. eLife. 11:e74161. 35775734 10.7554/eLife.74161 PMC9307275

[29] Morris WF, Ehrlén J, Dahlgren JP, Loomis AK, Louthan AM. 2020. Biotic and anthropogenic forces rival climatic/abiotic factors in determining global plant population growth and fitness. Proc Natl Acad Sci U S A. 117:1107–1112. 31888999 10.1073/pnas.1918363117 PMC6969536

[30] Clark-Wolf TJ, Dee Boersma P, Rebstock GA, Abrahms B. 2023. Climate presses and pulses mediate the decline of a migratory predator. Proc Natl Acad Sci U S A. 120:e2209821120. 36623194 10.1073/pnas.2209821120 PMC9934075

[31] Paniw M, et al 2021. The myriad of complex demographic responses of terrestrial mammals to climate change and gaps of knowledge: a global analysis. J Anim Ecol. 90:1398–1407. 33825186 10.1111/1365-2656.13467

[32] Benton TG, Plaistow SJ, Coulson TN. 2006. Complex population dynamics and complex causation: devils, details and demography. Proc Biol Sci. 273:1173–1181. 16720388 10.1098/rspb.2006.3495 PMC1560275

[33] Radchuk V, Turlure C, Schtickzelle N. 2013. Each life stage matters: the importance of assessing the response to climate change over the complete life cycle in butterflies. J Anim Ecol. 82:275–285. 22924795 10.1111/j.1365-2656.2012.02029.x

[34] Gamelon M, et al 2017. Interactions between demography and environmental effects are important determinants of population dynamics. Sci Adv. 3:e1602298. 28164157 10.1126/sciadv.1602298 PMC5287705

[35] Quéroué M, et al 2021. Multispecies integrated population model reveals bottom-up dynamics in a seabird predator–prey system. Ecol Monogr. 91:e01459.

[36] Caswell H . Matrix population models: construction, analysis, and interpretation. 2nd ed. Sinauer Associates, 2001.

[37] Morris WF, et al 2008. Longevity can buffer plant and animal populations against changing climatic variability. Ecology. 89:19–25. 18376542 10.1890/07-0774.1

[38] Malchow A-K, Hartig F, Reeg J, Kéry M, Zurell D. 2023. Demography–environment relationships improve mechanistic understanding of range dynamics under climate change. Philos Trans R Soc Lond B Biol Sci. 378:20220194. 37246385 10.1098/rstb.2022.0194 PMC10225853

[39] García-Callejas D, Molowny-Horas R, Retana J. 2017. Projecting the distribution and abundance of Mediterranean tree species under climate change: a demographic approach. J Plant Ecol. 10:731–743.

[40] Koricheva J, Gurevitch J, Mengersen K. Handbook of meta-analysis in ecology and evolution. Princeton University Press, 2013.

[41] Healy K, Ezard THG, Jones OR, Salguero-Gómez R, Buckley YM. 2019. Animal life history is shaped by the pace of life and the distribution of age-specific mortality and reproduction. Nat Ecol Evol. 3:1217–1224. 31285573 10.1038/s41559-019-0938-7

[42] Morris WF, Doak DF. 2004. Buffering of life histories against environmental stochasticity: accounting for a spurious correlation between the variabilities of vital rates and their contributions to fitness. Am Nat. 163:579–590. 15122504 10.1086/382550

[43] Forcada J, Trathan PN, Murphy EJ. 2008. Life history buffering in Antarctic mammals and birds against changing patterns of climate and environmental variation. Glob Chang Biol. 14:2473–2488.

[44] Turchin P . Population regulation: old arguments and a new synthesis. In: Cappuccino N, Price PW, editors. Population dynamics. Elsevier, 1995. p. 19–40.

[45] Tyler NJC, Forchhammer MC, Øritsland NA. 2008. Nonlinear effects of climate and density in the dynamics of a fluctuating population of reindeer. Ecology. 89:1675–1686. 18589531 10.1890/07-0416.1

[46] Smallegange IM, Guenther A. 2024. A development-centric perspective on pace-of-life syndromes. Evol Lett. 9:172–183 40191411 10.1093/evlett/qrae069 PMC11968188

[47] Van De Walle J, et al 2023. Individual life histories: neither slow nor fast, just diverse. Proc Biol Sci. 290:20230511. 37403509 10.1098/rspb.2023.0511 PMC10320331

[48] Buderman FE, Devries JH, Koons DN. 2023. A life-history spectrum of population responses to simultaneous change in climate and land use. J Anim Ecol. 92:1267–1284. 36995500 10.1111/1365-2656.13919

[49] Core Writing Team, Lee H, Romero J, editors. Climate change 2023: synthesis report. Contribution of working groups I, II and III to the sixth assessment report of the intergovernmental panel on climate change. IPCC, Geneva, Switzerland, 2023.

[50] Thomas CD, et al 2011. A framework for assessing threats and benefits to species responding to climate change. Methods Ecol Evol. 2:125–142.

[51] King KE, et al 2024. Increasing prevalence of hot drought across western North America since the 16th century. Sci Adv. 10:eadj4289 . 38266096 10.1126/sciadv.adj4289 PMC10807802

[52] González-Suárez M, Revilla E. 2013. Variability in life-history and ecological traits is a buffer against extinction in mammals. Ecol Lett. 16:242–251. 23216830 10.1111/ele.12035

[53] Feller U, Vaseva II. 2014. Extreme climatic events: impacts of drought and high temperature on physiological processes in agronomically important plants. Front Environ Sci. 2:39.

[54] Fuller A, et al 2021. How dryland mammals will respond to climate change: the effects of body size, heat load and a lack of food and water. J Exp Biol. 224:jeb238113 . 33627465 10.1242/jeb.238113

[55] Harris RM, et al 2018. Biological responses to the press and pulse of climate trends and extreme events. Nat Clim Chang. 8:579–587.

[56] Paniw M, et al 2022. Higher temperature extremes exacerbate negative disease effects in a social mammal. Nat Clim Chang. 12:284–290.

[57] Schmid M, Paniw M, Postuma M, Ozgul A, Guillaume F. 2022. A trade-off between robustness to environmental fluctuations and speed of evolution. Am Nat. 200:E16–E35. 35737989 10.1086/719654

[58] Zarnetske PL, Skelly DK, Urban MC. 2012. Biotic multipliers of climate change. Science. 336:1516–1518. 22723403 10.1126/science.1222732

[59] Stears AE, Heidel B, Paniw M, Salguero-Gómez R, Laughlin DC. 2024. Negative density dependence promotes persistence of a globally rare yet locally abundant plant species *Oenothera coloradensis*. Oikos. 2025(1):e10673.

[60] Woodroffe R, et al 2009. Social group size affects *Mycobacterium bovis* infection in European badgers (*Meles meles*). J Anim Ecol. 78:818–827. 19486382 10.1111/j.1365-2656.2009.01545.x

[61] Brandell EE, Dobson AP, Hudson PJ, Cross PC, Smith DW. 2021. A metapopulation model of social group dynamics and disease applied to Yellowstone wolves. Proc Natl Acad Sci U S A. 118:e2020023118. 33649227 10.1073/pnas.2020023118 PMC7958402

[62] Paniw M, et al 2023. Pathways to global-change effects on biodiversity: new opportunities for dynamically forecasting demography and species interactions. Proc Biol Sci. 290:20221494. 36809806 10.1098/rspb.2022.1494 PMC9943645

[63] Layton-Matthews K, Hansen BB, Grøtan V, Fuglei E, Loonen Mjje. 2020. Contrasting consequences of climate change for migratory geese: predation, density dependence and carryover effects offset benefits of high-Arctic warming. Glob Chang Biol. 26:642–657. 31436007 10.1111/gcb.14773

[64] Herrando-Pérez S, Delean S, Brook BW, Bradshaw CJA. 2012. Strength of density feedback in census data increases from slow to fast life histories. Ecol Evol. 2:1922–1934. 22957193 10.1002/ece3.298 PMC3433995

[65] Urban MC, et al 2022. Coding for life: designing a platform for projecting and protecting global biodiversity. BioScience. 72:91–104.

[66] Nater CR, Eide NE, Pedersen ÅØ, Yoccoz NG, Fuglei E. 2021. Contributions from terrestrial and marine resources stabilize predator populations in a rapidly changing climate. Ecosphere. 12:e03546.

[67] Travis JMJ, et al 2013. Dispersal and species’ responses to climate change. Oikos. 122:1532–1540.

[68] Snover AK, et al 2013. Choosing and using climate-change scenarios for ecological-impact assessments and conservation decisions. Conserv Biol. 27:1147–1157. 24299081 10.1111/cobi.12163

[69] van der Sluijs JP . 2020. Insect decline, an emerging global environmental risk. Curr Opin Environ Sustain. 46:39–42.

[70] Wagner DL, Grames EM, Forister ML, Berenbaum MR, Stopak D. 2021. Insect decline in the Anthropocene: death by a thousand cuts. Proc Natl Acad Sci U S A. 118:e2023989118. 33431573 10.1073/pnas.2023989118 PMC7812858

[71] Boggs CL . 2016. The fingerprints of global climate change on insect populations. Curr Opin Insect Sci. 17:69–73. 27720076 10.1016/j.cois.2016.07.004

[72] Martin LJ, Blossey B, Ellis E. 2012. Mapping where ecologists work: biases in the global distribution of terrestrial ecological observations. Front Ecol Environ. 10:195–201.

[73] Konno K, et al 2020. Ignoring non-English-language studies may bias ecological meta-analyses. Ecol Evol. 10:6373–6384. 32724519 10.1002/ece3.6368 PMC7381574

[74] Powers SM, Hampton SE. 2019. Open science, reproducibility, and transparency in ecology. Ecol Appl. 29:e01822. 30362295 10.1002/eap.1822

[75] Levin SC, et al 2022. Rpadrino: an R package to access and use PADRINO, an open access database of integral projection models. Methods Ecol Evol. 13:1923–1929.

[76] Ehrlén J, Morris WF, von Euler T, Dahlgren JP. 2016. Advancing environmentally explicit structured population models of plants. J Ecol. 104:292–305.

[77] Stubben C, Milligan B. 2007. Estimating and analyzing demographic models using the popbio package in R. J Stat Softw. 22:1–23.

[78] Hartig F . 2016. *DHARMa: residual diagnostics for hierarchical (multilevel/mixed) regression models*. CRAN. [accessed 2025 Apr 5]. 10.32614/CRAN.package.DHARMa.

[79] Nakagawa S, Schielzeth H. 2013. A general and simple method for obtaining *R*^2^ from generalized linear mixed-effects models. Methods Ecol Evol. 4:133–142.

