# Supplementary material for: Comparative life-cycle analyses reveal interacting climatic and biotic drivers of population responses to climate change
Source: PNAS Nexus. 2025 Sep 5;4(9):pgaf286. doi: 10.1093/pnasnexus/pgaf286 (PMC12461854; doi:10.1093/pnasnexus/pgaf286)
Supplement: pgaf286_Supplementary_Data [file pgaf286_supplementary_data.pdf]

Supplementary Materials for

Comparative Life-Cycle Analyses Reveal Interacting Climatic and Biotic Drivers of Population Responses to Climate Change

Ickin *et al.*

* Corresponding authors: Esin Ickin and Maria Paniw. Email: ickin.esin@gmail.com; maria.paniw@ebd.csic.es

All data and code are freely available on this repository, and we intend to continue adding more to this database in the future: https://github.com/MariaPaniw/Comparative-demography-project

This PDF file includes:

Supplementary text

Figures S1 to S53

Tables S1 to S7

Supplementary text, figures, and tables

Selection of studies

We used the following selection criteria for a study to be included in our database:

- A study had to be conducted on a wild natural population, i.e., (33).
- Quantitative models had to link at least two climatic, or one climatic and one biotic driver, to at least one vital rates, e.g., (80).
- The above drivers needed to be continuous for us to calculate the maximum, minimum, mean, and standard deviation, making results comparable across studies. This also allowed for perturbations with covariation, accounting for observed values of other drivers when the focal driver was at its extremes, i.e., (31).
- To facilitate comparisons, climatic drivers had to be direct measures of temperature or precipitation, meaning it couldn't be a driver that influences climate, such as the Southern Annular Mode, i.e., *Catharacta lönnbergi* from (37).
- The study should have constructed a structured population model such as a matrix population model, integrated population model, integral projection model, or individual-based model (36, 80, 81).

To find suitable studies, we first searched open databases on structured population models. We searched through the original papers in COMADRE and COMPARDE databases on matrix population models (82, 83). We also searched the open database Padrino, which has been collecting studies that parameterized vital rates as functions of traits and other covariates to build integral projection models (75). We also examined the database collected in (76), who compiled information on studies examining the relationship between environmental drivers and population growth rates in plants using structured population models. Lastly, we considered the studies published in (33).

To consider additional papers that were not part of the previous databases, we also searched Web of Science (WoS). For this, we used the search term:

TS = ((“vital rate” OR demograph* OR population OR life-history OR “life history” OR model) AND (climat* OR precipitation OR rain* OR temperature OR weather OR density)).

We acknowledge that we could have missed other relevant studies if our search terms were not mentioned in the title, abstract, or key words. Initial selection of studies from the above-listed databases showed that it was difficult to obtain all necessary data for our analyses from studies published prior to 2016. This was because information in the papers was not sufficient to replicate the models, we could not reach the authors of the studies, or they were not able to provide all the necessary information. We therefore restricted our WoS search to the most recent years (2016-2023). This yielded over three million results. We ordered the results by relevance and scanned through the first 300 papers, as further results were not relevant to our selection criteria.

Sensitivity analyses

We used different perturbations of climatic variables in underlying vital-rate models to calculate long-term population growth rates – which approximate population fitness under environmental change. In cases where we constructed matrix population or integral projection models (see *Details on Study Species*)**,** we calculated the asymptotic population growth rate (λ), using the R package popbio (77) version 2.7. For individual-based models, we calculated λ as the long-term average of N_{t+1}/N_t after projecting the population dynamics for at least 50 time steps and discarding the first 5-50 time steps to exclude an effect of transient dynamics in simulations (see *Details on Study Species*). We verified visually that λ calculated from simulations converged, corresponding to a distribution of growth rates that fluctuated with the same magnitude and direction across simulations (Figs. S30 – S43).

All perturbations included calculating λ under minimum (d_{min}) and maximum (d_{max}) values of a climatic driver (d) observed during a study period. In doing so, we used the actual observed values of other covariates when the focal driver was at its minimum or maximum (covariation) to account for the full complexity of environmental fluctuations and their effects on demography. We compared these perturbations to simplified ones, where we kept the remaining environmental covariates in vital-rate models fixed at their average values (no covariation) when perturbing a focal driver, which is typically done in classic sensitivity analyses (38). We then calculated the absolute scaled sensitivities, |S|, for each population and climatic driver (31) (**Equation 1**):

$$|S| = \left|\frac{\lambda_{max} - \lambda_{min}}{(d_{max} - d_{min})/\mathrm{SD}_d}\right|$$

The denominator of |S| is the difference in the driver levels in SD (standard deviation) units. This allows to compare the sensitivities of λ to drivers that vary over different scales, i.e., across different studies (31). We used the absolute values of S, |S|, because we were interested in the magnitude of the driver's effects on λ rather than the direction.

We calculated |S| for each climatic variable in turn. For instance, given a hypothetical example of predictors X, Y, and Z, where X and Y are climatic drivers in vital-rate models (e.g., rainfall and temperature), we calculated |S| for X (i) based on maximum and minimum values of X and keeping Y and Z at their average values (without covariation); (ii) using observed values of Y and Z when X was at its maximum or minimum (with covariation). We then calculated |S| for Y (iii) based on maximum and minimum values of Y and keeping X and Z at their average values (no covariation); (iv) using observed values of X and Z when Y was at its maximum or minimum (covariation). We point to the mouse lemur case study as a straightforward example of these calculations, as the vital rates models and predictors here are relatively simple (https://github.com/MariaPaniw/Comparative-demography-project/blob/main/Files_MouseLemur/SensitivityAnalysis_MouseLemur.Rmd).

We calculated uncertainties around |S| from standard errors of regression coefficients or from MCMC posterior distributions in those cases where vital rates were modeled using Bayesian regression. In the first situation, we used parametric bootstrapping; that is, we simulated the distributions of the regression coefficients based on their mean and SE and then ran the sensitivity analyses again by taking 100 parameter samples from the distribution. In the case of Bayesian regressions, we took 100 samples directly from the MCMC posterior distributions. We also tested other parameterizations of sensitivities to assess how much our choice of how to assess sensitivities affected results (see *Alternative sensitivity parameterizations* below). All analyses were conducted in R version 4.2.2.

In most studies, we calculated λ for either a single (meta)population or a representative average population across the habitat range. For the eight bird species, Malchow et al. developed a model using data from two sources. The species included *Certhia familiaris*, *Linaria cannabina*, *Lophophanes cristatus*, *Prunella collaris*, *Prunella modularis*, *Pyrrhula pyrrhula*, *Sitta europaea*, and *Turdus torquatus*, and the data covered 2585 sites across Switzerland (39). Although the individual-based models were spatially explicit, we adopted the matrix model and simulated

mean population growth rates and mean sensitivities for each species across sites. Similarly, the 11 Mediterranean tree species *Fagus sylvatica*, *Quercus faginea*, *Quercus ilex*, *Quercus robur/petraea*, *Pinus nigra*, *Pinus pinea*, *Quercus suber*, *Pinus uncinata*, *Pinus halepensis*, *Pinus pinaster*, and *Pinus sylvestris* were located across the continental territory of Spain in a 1 km x 1 km grid system (40) and we first calculated the scaled sensitivities and then averaged across the grid. In the case of *Drosophyllum lusitanicum*, Conquet et al. (84) included eight distinct populations, for which we first conducted the sensitivity analyses separately, and then averaged the results across sites. The study species *Dracocephalum austriacum* and *Perisoreus infaustus* also included four and two populations, respectively, for which we again first calculated the sensitivities separately, and then averaged the results across sites. We did this averaging in the main analyses to compare results at the species level. However, we performed additional analyses where we separated the different populations for *Drosophyllum lusitanicum*, *Dracocephalum austriacum*, and *Perisoreus infaustus* (see Table S4).

To understand the underlying mechanisms influencing population-level sensitivities to climate change ($|S_c|$), we fit a global generalized linear mixed model (GLMM), assuming a Gamma distribution with a log link function (**Equation 2**):

$$\log(|S_c|) = \mu_1 + \beta_1 * \mathrm{COV} + \beta_2 * \mathrm{DENS} + \beta_3 * \log(\mathrm{MAT}) + \beta_4 * \log(\mathrm{VR}) + \beta_5 * \log(\mathrm{PAR}) + \beta_6 * (\mathrm{COV} \times \mathrm{DENS}),$$

where μ_1 is the intercept, β_1 is the slope for the variable covariation (COV) which is categorical (no/yes), β_2 is the slope for the variable density (DENS; i.e., density dependence explicitly included in vital-rate models) which is also categorical with two levels (no/yes), β_3 is the slope for the log-transformed age at sexual maturity (MAT), β_4 is the slope for the log-transformed total number of vital rates that had climatic or biotic covariates (VR), β_5 is the slope for the log-transformed mean number of parameters per vital rate (PAR), and β_6 is the slope for the interaction of covariation and density. To address potential phylogenetic differences or variances within species, taxonomic groups and species were integrated as nested random intercepts, and covariation was added as a random slope.

We also fitted a simpler model, where we averaged sensitivities $|S|$, based on perturbations that considered the full complexity (i.e., covariation) of environmental drivers, across all perturbed

drivers for each species. As Fig. S1 demonstrates, average |S| were significantly lower for species where vital-rate models included density dependence.

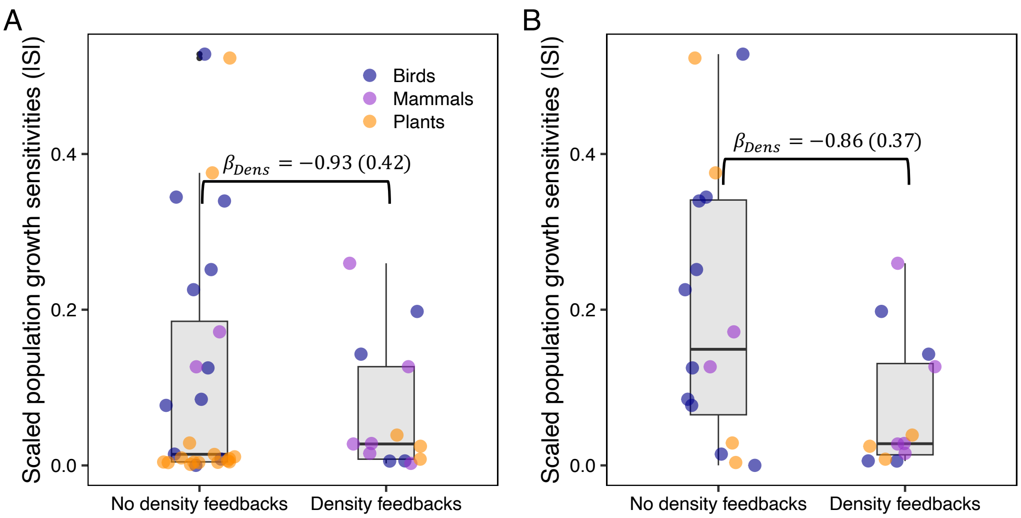

Figure S1. Scaled sensitivities of population growth rate to climate (|S|) averaged across all drivers for the 41 species used in the comparative analysis. The points represent the calculated averages, and the boxplots display the distribution of these averages among species where vital-rate models included or excluded density dependence. The boxplots show the median (central line), the interquartile range (box), and the range of the data (whiskers), with outliers shown as black points. A GLMM (with a Gamma link family) was used to model the average sensitivities as a function of presence or absence of density dependence, with species group as a random effect on the mean, and mean (SE) β estimates are shown. All calculations and plotting were done on the full dataset (A) or omitting species with very large or small ages at sexual maturity (B).

To investigate further whether the patterns (|S| lower for species where vital-rate models included density dependence) were driven by the effect of density dependence in vital-rate models, we performed additional perturbations for those species that modeled density dependence: We repeated the perturbations of climatic drivers considering covariation with other biotic and abiotic covariates, but not with intraspecific density (keeping density fixed). |S| increased for most populations with modeled density dependence when changes in the effects of density dependence were fixed in perturbations (Fig. S2).

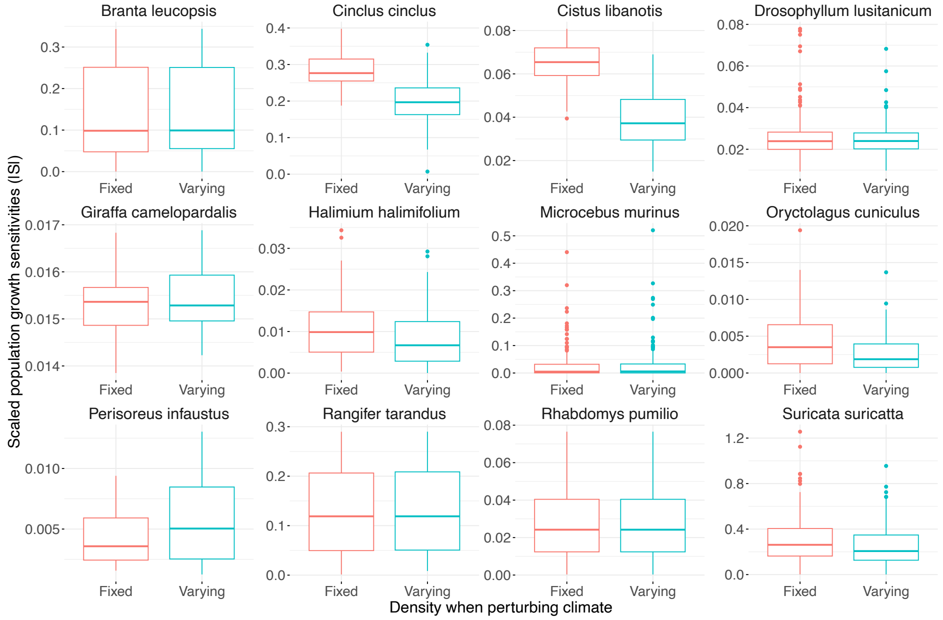

Figure S2. Scaled sensitivities of population growth rates to climate, |S|, for species where density dependence was considered explicitly in vital-rate models. |S| were calculated either keeping density fixed at their average values (but considering covariation with other environmental drivers) or considering covariation with density (along with other drivers, as in the global analysis in the main text) when perturbing a focal climate driver in vital-rate models. Boxplots summarize |S| across all resampled values for all focal climate drivers. Note that for *Perisoreus infaustus*, density has a positive effect on key vital rates, along with climatic variables, and density interacts non-linearly with climate (see (93)), which may explain why sensitivities increase under covariation of density and climate.

We then fitted an additional GLMM to see whether fixing interactions with density in full complexity perturbations still resulted in populations with density dependence having a lower |S| (**Equations 3**):

$$\log(|S_{cfixDensity}|) = \mu_1 + \beta_2 * \text{DENS} + \beta_3 * \log(\text{MAT}) + \beta_4 * \log(\text{VR}) + \beta_5 * \log(\text{PAR})$$

Parameter estimates from this model showed that, compared to results from the full global model (Equation 2), the effect of density decreases, and is not significant, $\beta_2 = -0.711(\pm 0.589)$ (see sensitivity_fixed_density.R).

We performed the global analyses (Equation 2) separately for plants, where we had a good representation of age at maturity and studies that included and excluded density dependence in vital-rate models. The results represented well the general results where all taxa were included

(Fig. S3). We note that we simplified the random error structure to allow the model to converge (Table S1).

Table S1. Output of model assessing how age at maturity, covariation with other drivers, presence of density dependence in vital-rate models, and other covariates affected scaled sensitivities of population growth rates of **plant species** to observed variation in climatic drivers.

A Fixed Effects	Coefficient	SE	P
Intercept	-0.788	1.743	0.651
$Covariation_{no}$	-0.483	0.042	**<0.001**
$Density_{yes}$	-1.102	1.081	0.308
Age at maturity	-1.386	0.296	**<0.001**
Number of Vital Rates	0.093	0.923	0.919
Parameters per Vital Rate	-0.149	0.577	0.796
$Covariation_{no}$:$Density_{yes}$	1.455	0.068	**<0.001**
B Random Effects	Variance	SD	Prop. variance
Species (Intercept)	0.396	0.629	0.483
Residual	0.454	0.674	0.517

Marginal R^2 (variance explained by fixed effects): 0.559

Conditional R^2 (variance explained by fixed and random effects): 0.785

The fixed effects (A) and random effects (B) of the generalized linear mixed model with gamma log link are shown here. The coefficient, standard error (SE), and p-value are reported for each fixed effect. Whereas variance and standard deviation (SD) are reported for each random effect. Random effects were incorporated due to multiple observations within species ($n_{samples}$ = 3420, $n_{species}$ = 18). $n_{samples}$ reflects all resampled |S| for each perturbation scenario and species to account for parameter uncertainty. Bold p-values indicate significance (α = 0.05). Prop. variance indicates the proportion of the total random-effect variance explained by different grouping variables.

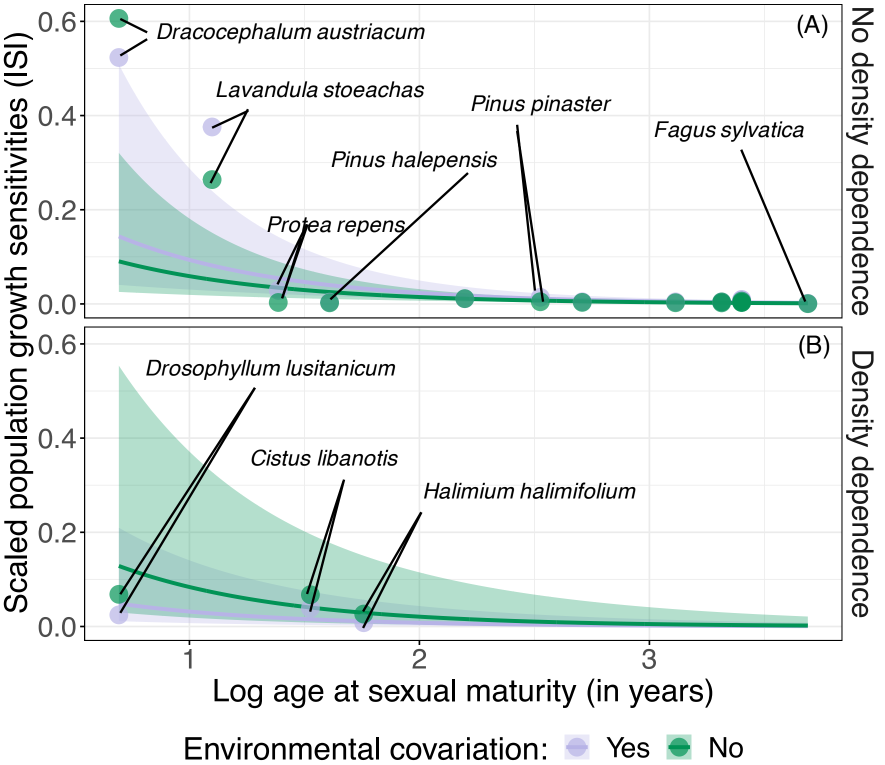

Figure S3. Scaled sensitivities of population growth rates to climate, |S|, across **plant species**. The age at sexual maturity in years (x-axis) is log-transformed using the natural logarithm. Sensitivities are shown for species where density dependence was not modeled (n =15) (A) or were added (n = 3) (B) as covariates in models. Different colors indicate sensitivity analyses under full environmental complexity (covariation with other drivers considered when perturbing a focal climate driver in vital-rate models) or reduced complexity (keeping other drivers as their average values when perturbing a focal driver). The lines represent predicted |S| over a range of ages of sexual maturity. The shaded areas indicate 95% model prediction intervals (see Table 1 for model coefficients). To aid visualization, the points show the observed sensitivity values of each species and perturbation scenario averaged over all perturbed climatic drivers and all resampled |S| under parameter uncertainty. We labeled some example species across different life histories and taxa.

We also assessed the differences between the sensitivities to temperature and rain ($|S_{TR}|$) by fitting another GLMM like above but this time untangling the climatic drivers (**Equation 4**):

$$\log(|S_{TR}|) = \mu_2 + \beta_1 * \text{COV} + \beta_2 * \text{DENS} + \beta_3 * \log(\text{MAT}) + \beta_4 * \text{DRIVER} + \beta_7 * (\text{COV} \times \text{DENS}) + \beta_8 * (\text{COV} \times \text{DRIVER}) + \beta_9 * (\text{DENS} \times \text{DRIVER}) + \beta_{11} * \log(\text{VR}) + \beta_{12} * \log\,(\text{PAR}),$$

where μ_2 is the intercept, α_1 is the slope for the variable covariation (COV) which is categorical (no/yes), β_2 is the slope for the variable density (DENS; i.e., density dependence present in vital-rate models) which is also categorical with two levels (no/yes), β_3 is the slope for the log-

transformed age at sexual maturity (MAT), β_4 is the slope for the driver (DRIVER), β_7 is the slope for the interaction of covariation and density, β_8 is the slope for the interaction of covariation and driver, β_9 is the slope for the interaction of density and driver, β_{10} is the slope for the log-transformed age at maturity (MAT), β_{11} is the slope for the log-transformed total number of vital rates that had climatic or biotic covariates (VR), and β_{12} is the slope for the log-transformed mean number of parameters per vital rate (PAR). To address potential phylogenetic differences or variances within species, taxonomic groups and species were integrated as nested random intercepts, and covariation was added as a random slope (Table S2; Fig. S4).

Table S2. Output of model assessing how age at maturity, covariation with other drivers, presence of density dependence in vital-rate models, driver type, and other covariates affected scaled sensitivities of population growth rates to changes in rain or temperature.

A Fixed Effects	Estimate	SE	P
Intercept	-3.474	1.010	**<0.001**
Covariation$_{no}$	-0.450	0.122	**<0.001**
Density$_{yes}$	-0.651	0.568	0.255
Driver$_{temp}$	0.297	0.028	**<0.001**
Age at Maturity	-0.983	0.204	**<0.001**
Number of Vital Rates	-0.141	0.526	0.788
Parameters per Vital Rate	0.748	0.501	0.136
Covariation$_{no}$:Density$_{yes}$	0.487	0.201	**0.015**
Covariation$_{no}$:Driver$_{temp}$	0.234	0.036	**<0.001**
Density$_{no}$:Driver$_{temp}$	-0.446	0.045	**<0.001**
B Random Effects	Variance	SD	Prop. variance
Species:Group			
Intercept	1.636	1.279	0.490
Covariation$_{yes}$	0.237	0.487	0.186
Group			
Intercept	<0.001	<0.001	<0.01
Covariation$_{yes}$	<0.001	<0.001	<0.01
Residual	0.715	0.846	0.324

Marginal R^2 (variance explained by fixed effects): 0.261

Conditional R^2 (variance explained by fixed and random effects): 0.824

The fixed effects (A) and random effects (B) of the generalized linear mixed model with gamma log link are shown here. The coefficient, standard error (SE), and p-value are reported for each fixed effect. Whereas variance and standard deviation (SD) are reported for each random effect. Random effects were incorporated due to multiple observations within species ($n_{samples}$ = 17’105, $n_{species}$ = 41, n_{groups} = 3). $n_{samples}$ reflects all resampled |S| for each perturbation scenario and species to account for parameter uncertainty. Bold p-values indicate significance (α = 0.05). Prop. variance indicates the proportion of the total random-effect variance explained by different grouping variables.

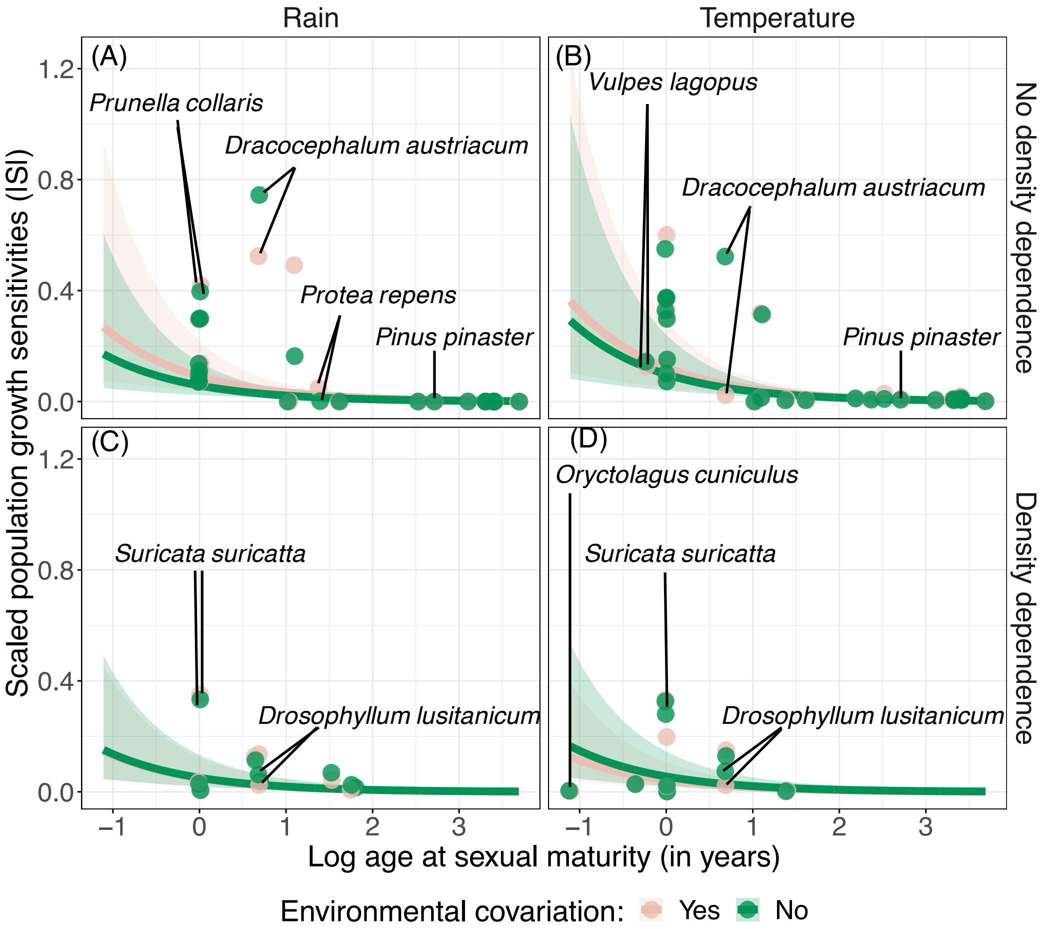

Figure S4. Scaled sensitivities of population growth rates to rain and temperature, |S|, across species. The age at sexual maturity in years (x-axis) is log-transformed using the natural logarithm. The sensitivities are shown for species where density dependence was not modeled in vital-rate models or were considered. Different colors indicate sensitivity analyses under full environmental complexity (covariation with other drivers considered when perturbing a focal climate driver in vital-rate models) or reduced complexity (keeping other drivers as their average values when perturbing a focal driver). The lines represent predicted |S| over a range of ages of sexual maturity. The shaded areas indicate 95% model prediction intervals (see Table 1 for model coefficients). To aid visualization, the points show the observed sensitivity values of each species and perturbation scenario averaged over all perturbed climatic drivers and all resampled |S| under parameter uncertainty.

We also tested how specific vital rates were driving $|S_{VR}|$ and fitted a GLMM using |S| that we computed by perturbing climatic drivers in single vital rates (see methods in main text; Fig. S5).

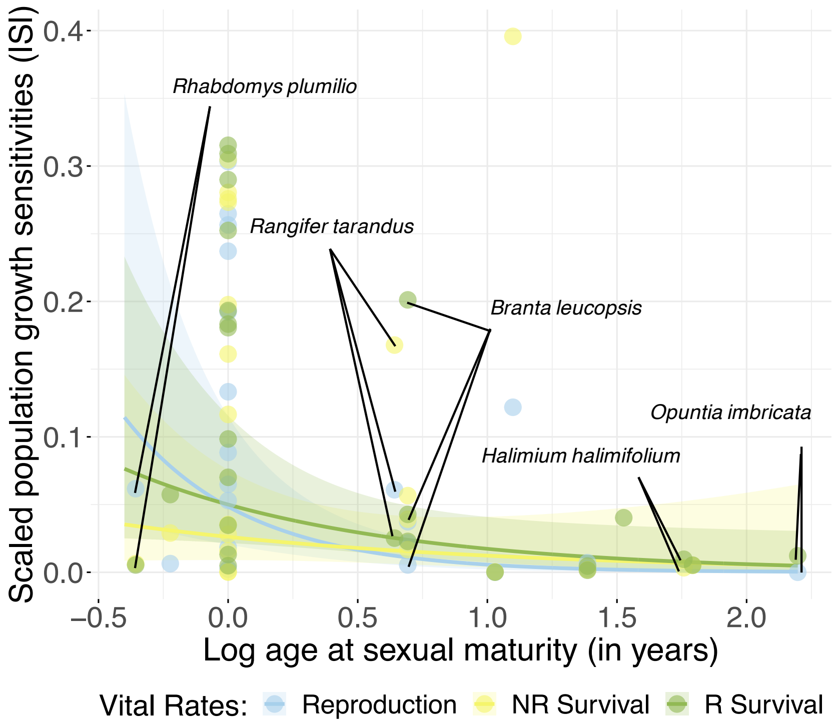

Figure S5. Scaled sensitivities of population growth rates to vital rates, |S|, across species. For each species, vital rates were categorized into three general categories (different colors. The age at sexual maturity in years (x-axis) is log-transformed using the natural logarithm. The shaded areas indicate 95% model prediction intervals. The points are average |S| per species and vital rate modeled.

To assess whether the length of the study affected any of our results, we included the variable study length as a covariate in the global GLMM (Equation 2). Study durations ranged from 3 to 40 years, with a mean of 21 years. Due to the wide range of study length, we used the natural logarithm of study length in the model. The results indicated that including study length as a covariate did not affect |S| ($\beta_{study\ length}$= -0.38 ± 0.47).

In addition, although we obtained the majority of λ values analytically and we checked that λ calculated from simulations, i.e., as the long-term average of (N_{t+1}/N_t), converged, we additionally evaluated statistically whether including λ calculated from simulations affected our results. To do so, we re-parameterized the global model above removing the subset of species where λ was calculated from simulations. The results remained unchanged, with the exception that the coefficient describing changes in sensitivities when perturbations were simplified ($Covariation_{no}$) showed relatively higher variability (see Table S3; Fig. S6).

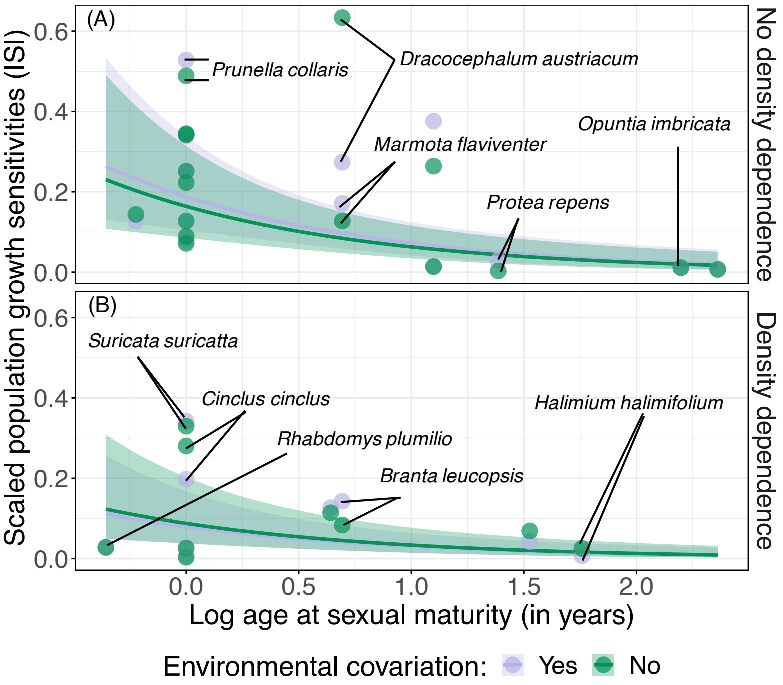

Figure S6. Scaled sensitivities of population growth rates to climate, |S|, **removing the subset of species where λ was calculated from simulations**. The age at sexual maturity in years (x-axis) is log-transformed using the natural logarithm. Sensitivities are shown for species where density dependence was not modeled (A) or were added (B) as covariates in models. Different colors indicate sensitivity analyses under full environmental complexity (covariation with other drivers considered when perturbing a focal climate driver in vital-rate models) or reduced complexity (keeping other drivers as their average values when perturbing a focal driver). The lines represent predicted |S| over a range of ages of sexual maturity. The shaded areas indicate 95% model prediction intervals (see Table 1 for model coefficients). To aid visualization, the points show the observed sensitivity values of each species and perturbation scenario averaged over all perturbed climatic drivers and all resampled |S| under parameter uncertainty. We labeled some example species across different life histories and taxa.

Table S3. Output of model assessing how age at sexual maturity, covariation with other drivers, presence of density dependence in vital-rate models and other covariates affected scaled sensitivities of population growth rates to changes in climate, |S|, **removing the subset of species where λ was calculated from simulations**.

A Fixed Effects	Coefficient	SE	P
Intercept	-2.377	0.724	**0.001**
$Covariation_{no}$	-0.137	0.141	0.337
$Density_{yes}$	-0.865	0.431	**0.045**
Age at sexual maturity	-0.958	0.266	**<0.001**
Number of vital rates	-0.316	0.357	0.377
Parameters per vital rate	0.701	0.391	0.081
$Covariation_{no}$:$Density_{yes}$	0.238	0.236	0.313
B Random Effects	Variance	SD	Prop. variance
Species/Group (Intercept)	0.813	0.902	0.388
Species/Group $Covariation_{no}$	0.263	0.517	0.222
Group (Intercept)	<0.001	<0.001	<0.01
Group $Covariation_{no}$	<0.001	<0.001	<0.01
Residual	0.823	0.908	0390
Marginal R^2 (variance explained by fixed effects): 0.338			
Conditional R^2 (variance explained by fixed and random effects): 0.748			

The fixed effects (A) and random effects (B) of the generalized linear mixed model with gamma log link are shown here. The coefficient, standard error (SE), and p-value are reported for each fixed effect, whereas variance and standard deviation (SD) are reported for each random effect. Nested random effects were incorporated due to multiple observations within species and groups ($n_{samples}$ = 14'566, $n_{species}$ = 25, n_{groups} = 3). $n_{samples}$ reflects all resampled |S| for each perturbation scenario and species to account for parameter uncertainty. Bold p-values indicate statistical significance (α =0.05). Prop. variance indicates the proportion of the total random-effect variance explained by different grouping variables.

Finally, we repeated the global model, but maintaining |S| separate for different populations of *Drosophyllum lusitanicum*, *Dracocephalum austriacum*, and *Perisoreus infaustus.* We thus included another nested level of the random effect: population nested in species, which in turn was nested in group (mammals, birds, plants). As Table S4 shows, the results remained unchanged, and variance among populations did not contribute substantially to the random effect variance.

Table S4. Output of model assessing how age at sexual maturity, covariation with other drivers, presence of density dependence in vital-rate models and other covariates affected scaled sensitivities of population growth rates to changes in climate, |S|, **including a population random effect**.

A Fixed Effects	Coefficient	SE	P
Intercept	-3.007	0.958	**0.002**
Covariation$_{no}$	-0.252	0.112	**0.024**
Density$_{yes}$	-1.000	0.559	0.071
Age at sexual maturity	-1.032	0.199	**<0.001**
Number of vital rates	-0.321	0.504	0.523
Parameters per vital rate	0.844	0.491	0.091
Covariation$_{no}$:Density$_{yes}$	0.389	0.190	**0.040**
B Random Effects	Variance	SD	Prop. variance
Population/Species/Group (Intercept)	0.015	0.123	0.015
Population/Species/Group Covariation$_{no}$	0.050	0.222	0.042
Species/Group (Intercept)	1.724	1.313	0.447
Species/Group Covariation$_{no}$	0.174	0.417	0.142
Group (Intercept)	<0.001	<0.001	<0.01
Group Covariation$_{no}$	<0.001	<0.001	<0.01
Residual	0.738	0.859	0.293
Marginal R^2 (variance explained by fixed effects): 0.302			
Conditional R^2 (variance explained by fixed and random effects): 0.829			

The fixed effects (A) and random effects (B) of the generalized linear mixed model with gamma log link are shown here. The coefficient, standard error (SE), and p-value are reported for each fixed effect, whereas variance and standard deviation (SD) are reported for each random effect. Nested random effects were incorporated due to multiple observations within species and groups ($n_{samples}$ = 17'666, $n_{species}$ = 41, n_{groups} = 3). $n_{samples}$ reflects all resampled |S| for each perturbation scenario and species to account for parameter uncertainty. Bold p-values indicate statistical significance (α =0.05). Prop. variance indicates the proportion of the total random-effect variance explained by different grouping variables.

We repeated the main analysis again but this time removing three study species that were conducted on multiple populations. As Table S5 shows, the results do not change whether we include multi-population species (Table 1) or only one population per species.

Table S5. Output of model assessing how age at sexual maturity, covariation with other drivers, presence of density dependence in vital-rate models and other covariates affected scaled sensitivities of population growth rates to changes in climate, |S|, **excluding three studies where vital-rate responses to drivers varied among different populations.**

A Fixed Effects	Coefficient	SE	P
Intercept	-3.083	1.067	**<0.001**
Covariation$_{no}$	-0.288	0.104	**0.006**
Density$_{yes}$	-0.684	0.578	0.236
Age at sexual maturity	-0.978	0.199	**<0.001**

Number of vital rates	-0.094	0.543	0.863
Parameters per vital rate	0.607	0.626	0.332
$Covariation_{no}$:$Density_{yes}$	0.413	0.185	**0.026**
B Random Effects	Variance	SD	Prop. variance
Species/Group (Intercept)	1.843	1.358	0.646
Species/Group $Covariation_{no}$	0.189	0.435	0.066
Group (Intercept)	<0.001	<0.001	<0.001
Group $Covariation_{no}$	<0.001	<0.001	<0.001
Residual	0.818	0.905	0.287
Marginal R^2 (variance explained by fixed effects): 0.229			
Conditional R^2 (variance explained by fixed and random effects): 0.766			

The fixed effects (A) and random effects (B) of the generalized linear mixed model with gamma log link are shown here. The coefficient, standard error (SE), and p-value are reported for each fixed effect, whereas variance and standard deviation (SD) are reported for each random effect. Nested random effects were incorporated due to multiple observations within species and groups ($n_{samples}$ = 15'640, $n_{species}$ = 38, n_{groups} = 3). $n_{samples}$ reflects all resampled |S| for each perturbation scenario and species to account for parameter uncertainty. Bold p-values indicate statistical significance (α =0.05). Prop. variance indicates the proportion of the total random-effect variance explained by different grouping variables.

<u>Alternative sensitivity parameterizations</u>

When using the same data to calculate the range (maximum-minimum) and SD of a variable, there is necessarily a positive correlation between those two metrics (Fig. S7). This means that our scaling approach (Equation 1) results in a denominator that is similar across different species. One way to break this correlation is to calculate long-term SD of climatic drivers, while taking the range of values over a given shorter-term study period. In our comparative analysis, it was not possible to calculate long-term SD of climatic drivers in many studies we examined. However, in all studies but one (on the gray mouse lemur, *Microcebus* murinus (80)) the covariates in vital rate models did not show a large range (Fig. S7), and covariates were already scaled to represent *z* scores (mean = 0; SD = 1 regardless of range) for 23 species of the 41 species (Fig. S7). In other words, the covariates were already on a similar scale across most studies. The grey mouse lemur was the only study that used raw climatic values with large ranges of temperatures (30°C – 32°C) and rainfall (621 mm – 1404 mm).

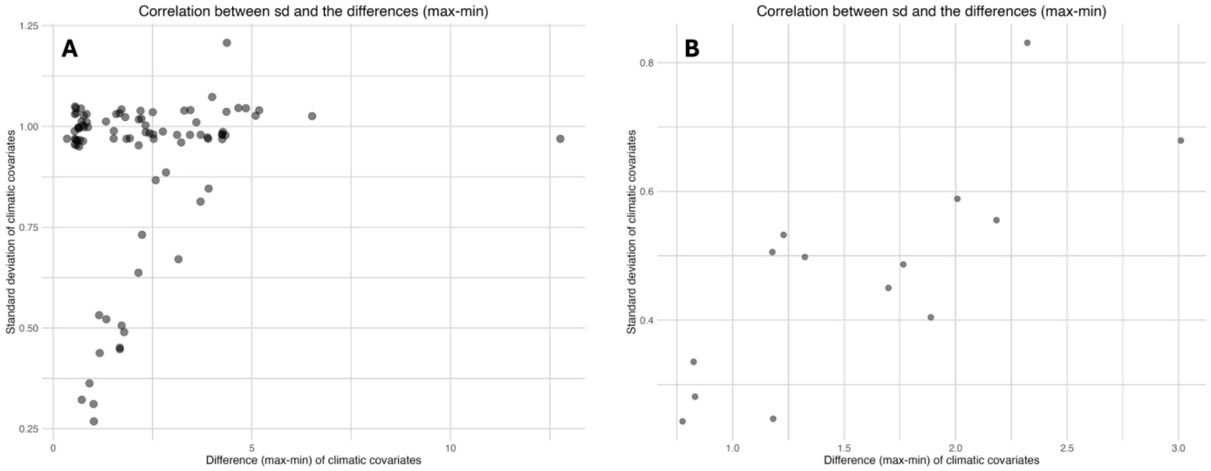

Figure S7. Relationship between observed ranges (maximum – minimum values) and standard deviations of climatic variables perturbed in this comparative study. These values were used to scale sensitivities of population growth rates, λ, across different studies (see Equation 1). The plots show (A) all ranges, including for studies where climatic drivers were already scaled in the original study (SD = 1); or (B) removing the latter drivers.

In addition, we calculated another sensitivity metric to test whether our scaling impacted our conclusions. We calculated the log response ratios (|L|) of perturbed population growth rates, λ, (**Equation 5**):

$$|L| = |\log(\lambda_{max}/\lambda_{min})|,$$

where *min* and *max* refer to λ calculated at the minimum and maximum values of a climatic driver. As with |S|, we considered absolute values as we were interested in the magnitude of the effects only. Log response ratios are the most common type of metric for summarizing outcomes in ecological meta-analyses (85, 86), but do not account for different scales in perturbations (31). As Fig. S8 and Table S6 show, our conclusions remain largely unchanged when considering |L| as sensitivity metric (except for a higher uncertainty associated with the main effect of "*Density in vital rate modes*"). This highlights that our results are not sensitive to different sensitivity parameterizations.

Table S6. Output of model assessing how age at sexual maturity, covariation with other drivers, presence of density dependence in vital-rate models and other covariates affected log response ratios, |L|.

A Fixed Effects	Coefficient	SE	P
Intercept	-2.587	0.894	**0.004**
Covariation_{no}	-0.281	0.127	0.027
Density_{yes}	-0.561	0.510	0.254
Age at sexual maturity	-0.378	0.181	**0.036**
Number of vital rates	0.209	0.464	0.652
Parameters per vital rate	0.451	0.451	0.316
$\text{Covariation}_{no}\text{:Density}_{yes}$	0.455	0.219	**0.037**
B Random Effects	Variance	SD	Prop. variance
Species/Group (Intercept)	1.954	1.398	0.470
Species/Group Covariation_{no}	0.374	0.612	0.206
Group (Intercept)	<0.001	<0.001	<0.01
Group Covariation_{no}	<0.001	<0.001	<0.01
Residual	0.933	0.966	0325
Marginal R^2 (variance explained by fixed effects): 0.101			
Conditional R^2 (variance explained by fixed and random effects): 0.761			

The fixed effects (A) and random effects (B) of the generalized linear mixed model with gamma log link are shown here. The coefficient, standard error (SE), and p-value are reported for each fixed effect, whereas variance and standard deviation (SD) are reported for each random effect. Nested random effects were incorporated due to multiple observations within species and groups ($n_{samples}$ = 16’805, $n_{species}$ = 41, n_{groups} = 3). $n_{samples}$ reflects all resampled |S| for each perturbation scenario and species to account for parameter uncertainty. Bold p-values indicate statistical significance (α =0.05). Prop. variance indicates the proportion of the total random-effect variance explained by different grouping variables.

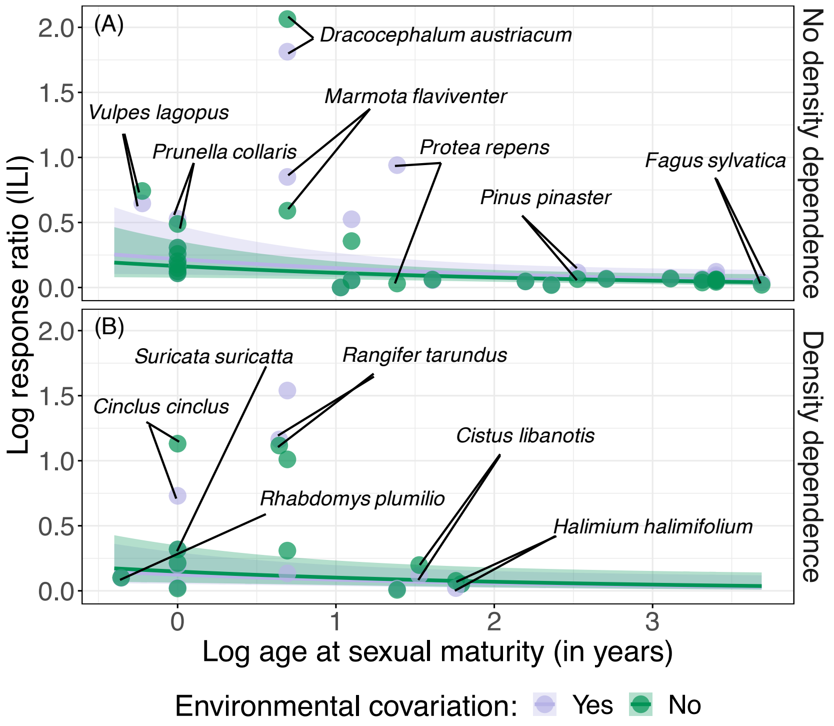

Figure S8. Log response ratios (|L|) of population growth rates, λ, under perturbations of climatic variables in vital-rate models. The age at sexual maturity in years (x-axis) is log-transformed using the natural logarithm. Response ratios are shown for species where density dependence was not modeled (A) or were added (B) as covariates in vital-rate models. Different colors indicate response ratios under full environmental complexity (covariation with other drivers considered when perturbing a focal climate driver in vital-rate models) or reduced complexity (keeping other drivers as their average values when perturbing a focal driver). The lines represent predicted |L| over a range of ages of sexual maturity. The shaded areas indicate 95% model prediction intervals (see Table 1 for model coefficients). To aid visualization, the points show the observed sensitivity values of each species and perturbation scenario averaged over all perturbed climatic drivers and all resampled |S| under parameter uncertainty. We labeled some example species across different life histories and taxa.

Details on study species

Table S7. Overview of all the species included in this comparative study. The covariates used in the models were temperature (T), precipitation (P), intraspecific density (D1), interspecific density (D2), southern annular mode (SAM), sea surface temperature (SST), rain-on-snow (ROS), sea-ice concentration (SIC), food (B), potential evapotranspiration (PET), and latent climatic variable (Q). The IUCN status comprises of least concern (LC), vulnerable (VU), data deficient (DD), and unknown (?). The population trend categories, also obtained from the IUCN red list, are stable (=), increasing (+), decreasing (-), or unknown (?). The sources of the original studies the data were obtained from are listed in sources, alongside the sources of IUCN status, population trend, and age at sexual maturity if it was not obtained from the original study.

Species	Common name	Covariates	IUCN status	Population trend	Age at sex. maturity (years)	Source
Certhia familiaris	Eurasian treecreeper	T, P	LC	=	1	39, 114
Linaria cannabina	Common linnet	T, P	LC	-	1	39, 115
Lophophanes cristatus	Crested tit	T, P	LC	-	1	39, 116
Prunella collaris	Alpine accentor	T, P	LC	=	1	39, 117
Prunella modularis	Dunnock	T, P	LC	-	1	39, 118
Pyrrhula pyrrhula	Eurasian bullfinch	T, P	LC	-	1	39, 119
Sitta europaea	Eurasian nuthatch	T, P	LC	=	1	39, 120
Turdus torquatus	Ring ouzel	T, P	LC	=	1	39, 121
Cinclus cinclus	White-throated dipper	T, D1	LC	-	1	36, 88, 122
Halobaena caerulea	Blue petrel	SST, SAM, D1, D2, B, SAM	LC	-	4	37, 88, 123
Thalassarche melanophris	Black-browed albatross	SST winter, SST breeding season	LC	+	10.6	88,89, 124
Spheniscus magellanicus	Magellanic penguin	T, P, SST	LC	-	2.8	32, 88, 125
Microcebus murinus	Gray mouse lemur	T, P, D1	LC	-	1	80, 126
Rangifer tarandus	Reindeer	ROS, D1	VU	-	1.9	88, 127
Vulpes lagopus	Arctic fox	T, B	LC	=	0.8	88, 66, 128

Rhabdomys pumilio	Striped mouse	T, D1, B	LC	=	0.7	12, 103, 129
Marmota flaviventer	Yellow-bellied marmot	Q, D1	LC	=	2	99, 130
Suricata suricatta	Meerkat	T, P, D1	LC	=	1	5, 131
Giraffa camelopardalis	Masai giraffe	P, D1	VU	-	6	81, 132
Protea repens	Sugarbush	T, P	LC	=	4	111, 112, 133
Fagus sylvatica	Beech	T, P	LC	?	40	40, 134, 135
Quercus faginea	Honeydew oak	T, P	LC	?	30	40, 136, 137
Quercus ilex	Holly oak	T, P	LC	=	30	40, 136, 138
Quercus robur	Common oak	T, P	LC	-	30	40, 136, 140
Pinus nigra	Black pine	T, P	LC	=	27.5	40, 141, 142
Pinus pinea	Stone pine	T, P	LC	=	22.5	40, 142, 143
Quercus suber	Cork oak	T, P	LC	-	30	40, 136, 144
Pinus uncinata	Mountain pine	T, P	LC	=	15	40, 145, 146
Pinus halepensis	Aleppo pine	T, P	LC	=	5	40, 142, 147
Pinus pinaster	Maritime pine	T, P	LC	+	12.5	40, 142, 148
Pinus sylvestris	Scots pine	T, P	LC	=	27.5	40, 142, 149
Drosophyllum lusitanicum	Dewy pine	T, D1	?	-	2	106
Halimium halimifolium	Yellow sun rose	P, D1, D2	?	-	5.8	62
Cistus libanotis	Rockrose	P, D1, D2	LC	-	4.6	62, 150
Opuntia imbricata	Devil's rope pear	T (multiple)	LC	+	9	109, 110, 151
Dracocephalum austriacum	Austrian dragonhead	T, P, PET	DD	-	2	105, 152
Branta leucopsis	Barnacle goose	T, P, D1, B	LC	=	2	153
Perisoreus infaustus	Siberian jay	P,T,D1	LC	=	1	154
Oryctolagus cuniculus	European rabbit	T,D1,B	EN	-	0.33	63
Lavandula stoechas	Lavender	T, P	?	?	3	155

Aptenodytes forsteri	Emperor penguin	SIC (multiple)	NT	-	3	156

Birds

Blue Petrel (*Halobaena caerulea*). The population of blue petrels was studied on Mayes Island in the Southern Ocean where they breed during the austral summer (37). This species is long-lived and reaches sexual maturity at the age of four years (37). The climate and population size information were obtained from the a GitHub repository (https://github.com/maudqueroue/MultispeciesIPM_SkuaPetrel) and the code for the vital rate models and the population model, as well as the regression coefficients were provided by the corresponding author of (37). The authors built a multispecies integrated population model where the covariates in the vital-rate models for the petrel were: the Southern annular mode, sea surface temperature anomalies (SSTA), chlorophyll *a* concentration, and intra- and interspecific density. We classified SSTA as the only climatic driver "temperature". We calculated λ by projecting the population for 20 years, discarding the first ten years to account for transient dynamics. λ was then determined by calculating the changes in abundance per year using the formula and averaging it. We calculated uncertainties around λ for each perturbation scenario by resampling regression coefficients from the MCMC posteriors 10 times (instead of 50 or 100 due to the limits of computational power); and then recalculating λ.

The White-throated Dipper (*Cinclus cinclus*). The study population of the white-throated dipper is located in the river system of Lyngdalselva in southern Norway (36). The dipper is a small short-lived passerine bird with the average age at sexual maturity of one year (87, 88). The climate data and population size information were obtained from the corresponding author of (36). The structure of the vital-rate models (survival and recruitment rates of the age classes 1-4) and the regression coefficients were obtained from the paper's supplementary materials Table S1 and Table S2, respectively (36). The study integrated the recorded number of occupied nests, capture-recapture data of females, and data on reproductive success into a Bayesian integrated population model (36). We built the matrix population model based on the life cycle illustrated in Figure 1 of the study (36). Noticeably, the immigration rate was added as apparent recruitment per capita to age class 1 in our population model, after discussing it with the corresponding author. The covariates used in the vital-rate models were standardized mean winter temperature and density. We classified the former as "temperature" for our GLMMs. We calculated λ as the

dominant eigenvalue of the matrix model for each perturbation scenario. We calculated uncertainties around λ for each perturbation scenario by resampling regression coefficients 100 times using a gaussian distribution with SE given by the study; and then recalculating λ. The authors of (36) performed standard sensitivity analyses of population growth rate, λ, and our results on the strength of different drivers on population dynamics are in line with these previous analyses (see Fig. S13).

The Magellanic Penguin (*Spheniscus magellanicus*). The study population of the Magellanic penguins is located at the Punta Tombo colony in Argentina (32). They typically reach sexual maturity at the age of 2.8 years (32). The data and R code were obtained from the corresponding author's GitHub repository (https://github.com/teejclark/Press_Pulse) (32). Clark-Wolf and colleagues built a pre-breeding, three-stage, female-only integrated population model (32). The covariates used in the vital-rate models were the total precipitation between October 15 and December 15, temperature as the % of days per breeding season when maximum air temperature was higher than 25 °C, and sea surface temperature anomalies during breeding and migration season and their lagged versions. We classified precipitation as "rain" and the rest as "temperature" for our GLMMs. We calculated λ by running the model for 38 years, calculating λ = (N_{t+1}/N_t), and averaging it across all years. We only used the last 20 λ, discarding the first 18 years to account for transient dynamics. We calculated uncertainties around λ for each perturbation scenario by resampling regression coefficients from the MCMC posteriors that we obtained from the IPM 100 times; and then recalculating λ.

Swiss Birds. The study included eight Swiss breeding bird populations: Eurasian bullfinch (*Pyrrhula pyrrhula*), European crested tit (*Lophophanes cristatus*), Eurasian treecreeper (*Certhia familiaris*), Eurasian nuthatch (*Sitta europaea*), dunnock (*Prunella modularis*), common linnet (*Linaria cannabina*), ring ouzel (*Turdus torquatus*), and alpine accentor (*Prunella collaris*). The authors of the study chose bird species with age at maturity of one year that share common traits (39). The climate data and the code were obtained from the author's GitHub repository: https://github.com/UP-macroecology/Malchow_DemogEnv_2022, and the regression coefficients for the models were provided directly by the authors (39). A female-only, two-stage matrix population model with three vital rates was built for each species (39). In the vital-rate models, five climatic covariates were used: mean temperature and total precipitation during the breeding season, mean temperature in fall, and total precipitation and minimum temperature during winter. Temperature-related covariates were categorized as "temperature", and precipitation-related ones as "rain". We calculated λ as the dominant eigenvalue of the matrix model for each perturbation

scenario and for each species separately. We calculated uncertainties around λ for each perturbation scenario by resampling regression coefficients from the MCMC posteriors 100 times, and then recalculating λ. We conducted the analyses for each species separately, but they all followed the same workflow. Although the individual-based models were spatially explicit (covering 2585 sites across Switzerland) (39), we adopted the matrix model and simulated mean λ and mean |S| for each species across sites.

The Black-browed Albatross (*Thalassarche melanophris*). The study population is located at Kerguelen Island, in the colony of Cañon des Sourcils Noirs (89). The black-browed albatross is a long-lived seabird, reaching sexual maturity at the age of 10.6 years (88, 90). The climate data and code, including the model parameters, were provided by the corresponding author (89). The authors built a matrix population model comprised of 25 states. The covariates used in the vital-rate models were standardized sea surface temperature (SST) in the juvenile sector during the wintering season (May to August), SST in the wintering sector of adults (July to September), and SST in the breeding sector (October of year t to March of year t+1) (89). We classified all the climatic covariates as “temperature”. We calculated λ as the dominant eigenvalue of the matrix model for each perturbation scenario. We calculated uncertainties around λ for each perturbation scenario by resampling regression coefficients 100 times using a gaussian distribution with SE extracted from Table S2.4b of a previous study (91); and then recalculating λ.

The Barnacle Goose (*Branta leucopsis*). The study population was monitored in northwestern Svalbard where it breeds (63). The Svalbard barnacle goose population overwinters at Solway Firth, Scotland, before flying to Svalbard for breeding in summer. The barnacle goose reaches sexual maturity at the age of 2 years (92). The climate data and code, including the model parameters, were provided by the corresponding author (63). The authors built a matrix population model comprised of 2 states, fledglings and adults. The covariates used in the vital-rate models were mean daily minimum temperatures October-March in Scotland and in April-May in Helgeland, mean precipitation in April-May in Helgeland, the flyway population size at the wintering grounds in Scotland, spring onset, adult numbers in Svalbard, and fox predation. We classified all the climatic covariates as “temperature” or “rainfall”. We calculated λ as the dominant eigenvalue of the matrix model for each perturbation scenario. We calculated uncertainties around λ for each perturbation scenario by resampling regression coefficients 100 times using a multivariate Normal distribution based on the parameter covariance matrix. The

authors of (63) performed standard sensitivity analyses of population growth rate, λ, and our results on the strength of different drivers on population dynamics are in line with these previous analyses (see Fig. S24).

The Siberian Jay (*Perisoreus infaustus*). Siberian jay individuals have been observed long-term near Arvidsjaur, northern Sweden. For this study, we had 15 years of data on 4341 sightings from 1166 individuals (93). We used population models representing two populations: managed (in the southern area where scots pine and Norway spruce are thinned, harvested, and re-planted in 80–120 year cycles) and natural (northern area of the study site that has not been managed for at least 200 years). The jays reach sexual maturity at the age of 1 year. The climate data and code, including the model parameters, were provided by the corresponding author (93). The authors built a periodic matrix population model that described transitions among juvenile, non-breeding, and breeding stages across winter and summer seasons. The covariates used in the vital-rate models were mean winter snow depth (December–March), average temperature during the breeding season (April–May), and population density. We classified all the climatic covariates as "temperature" or "precipitation". We calculated λ as the dominant eigenvalue of the annual product of the periodic matrix model for each perturbation scenario. We calculated uncertainties around λ for each perturbation scenario by resampling regression coefficients 100 times using a multivariate Normal distribution based on the parameter covariance matrix. We calculated sensitivities for the natural and managed population separately, and the averaged them across the two populations for the global analysis. The authors of (93) performed standard sensitivity analyses of population growth rate, λ, and our results on the strength of different drivers on population dynamics are in line with these previous analyses (see Fig. S23).

The emperor penguin (*Aptenodytes forsteri*). For this work, we used a long-term dataset on breeding emperor penguins at Dumont D'Urville, Terre Adélie, in Antarctica. The colony has been monitored every year, during the breeding season (March–December), from 1962 onwards. We used the demographic model in (94) constructed from capture histories from 1962-2005. The authors constructed a sex- (males and females) and stage-structured (pre-breeders, breeding pairs, non-breeder) periodic (seasonal) matrix population model following (95). The climatic covariates in vital-rate models were proportional anomalies in sea-ice concentration (SIC), relative to the mean from 1979 to 2007 in the pre-breeding, laying, incubating, and rearing seasons. We categorized the SIC as "temperature" in our GLMMs. All data and code to construct and perturb

the population model were made available by Jenouvrier and coauthors and can be found at: https://gitfront.io/r/fledge-whoi/zUbHbQtJq2XV/emperor-penguin-IUCN/ (CMR code). We calculated λ as the dominant eigenvalue of the annual product of the seasonal matrix population models for each perturbation scenario, after projecting population dynamics for 1000 years and letting the population vector converge to a stable distribution (projections were necessary because female/male ratios were used to model breeding and were generated within the model). We obtained the uncertainties around λ for each perturbation scenario using parametric bootstrapping to obtain variation in vital rate parameters (following the original study; (94)).

Mammals

The Masai Giraffe (*Giraffa camelopardalis tippelskirchi or G. tippelskirchi*). The metapopulation of female Masai giraffes studied here is located in northern Tanzania (96-98). They reach sexual maturity at the age of 6 years (81). The data and code were provided by the authors of (81) and can be found at https://github.com/MariaPaniw/Masai_giraffe_ibm. The study used long-term demographic data to develop a stochastic, socially structured individual-based model (IBM) (81). The two covariates used in the model were population density and rainfall; the latter was classified as “rain” for our GLMMs. We ran the model for 150 seasons (4-month time steps), discarding the first 12 seasons to account for transient dynamics. λ was then determined by calculating the changes in abundance per year using the formula $\lambda = (N_{t+1}/N_t)$ and averaging it across all years. We ran the simulation 100 times to obtain the uncertainties around λ for each perturbation scenario.

The Yellow-bellied Marmot (*Marmota flaviventer*). The study population of these large rodents is located in the Upper East River Valley, Gothic, Colorado. They reach sexual maturity at the age of 2 years (99). The data and code of the study were provided by the author of the study (99) and can also be found at https://datadryad.org/stash/dataset/doi:10.5061/dryad.4j0zpc87c. The authors built seasonal stage-, mass- and environmental-specific integral projection models (IPM) that account for seasonal demographic covariation using a latent climatic variable (Q) that depicts a measure of environmental quality (99). We considered random year variation as a separate covariate, due to the way the demographic model was built. We considered Q as a climatic driver and composite of both rainfall and temperature (see (99)). We calculated λ as the dominant eigenvalue of the matrix of the IPM for each perturbation scenario. We calculated uncertainties around λ for each perturbation scenario by resampling regression coefficients 100 times from the

MCMC posteriors, and then recalculating λ. The authors of (99) performed standard sensitivity analyses of the stochastic population growth rate, λs, and our results on the strength of different drivers on population dynamics are in line with these previous analyses (see Fig. S26).

The Gray Mouse Lemur (*Microcebus murinus*). The study population of this small lemur is located in the Kirindy forest in Madagascar (100). They are a short-lived species, reaching sexual maturity at the age of one year (80, 101, 102). The climate and population-size data were provided by the corresponding author of (80). We obtained the structure of the vital-rate models, regression coefficients, and their standard errors from Table 1 of their paper. We then rebuilt the MPM based on the annual life cycle illustrated in Figure 6 of their paper (80). The model is a two-stage and two-sex matrix population model (80). The covariates used in the vital-rate models were monthly mean maximum temperature, monthly total rainfall, and population density. We classified mean maximum temperature as "temperature" and total rainfall as "rain" for our GLMMs. We calculated λ as the dominant eigenvalue of the matrix model for each perturbation scenario. We calculated uncertainties around λ for each perturbation scenario by resampling regression coefficients 100 times using a gaussian distribution with SE given by the study; and then recalculating λ.

The Svalbard Reindeer (*Rangifer tarandus*). The study population of the wild Svalbard reindeer is located in central Spitsbergen, Svalbard, Norway (7). They reach sexual maturity at the age of 1.9 years (88). The climate data, population size information, and posterior samples were obtained from the authors (7). The model used was an integrated population model with six female age classes. The covariates in the vital rate models were rain-on-snow (ROS), population density, and winter length. We did not include sensitivities to winter length since it was not related to temperature or rain. ROS was classified as "rain" for our GLMMs. We calculated λ as the dominant eigenvalue of the matrix model for each perturbation scenario. We calculated uncertainties around λ for each perturbation scenario by resampling regression coefficients 100 times from the MCMC posteriors, and then recalculating λ.

The African Striped Mouse (*Rhabdomys pumilio*). The short-lived African striped mouse lives in the dry regions of South Africa and reaches sexual maturity within the first year; for our study, we

set this parameter to 0.7 years (12, 103). The climate and population-size data, as well as the full code of the population model was provided by the corresponding author (12). The model built in the study was a female-only stage-structured matrix population model (12). The covariates utilized in the vital-rate models included monthly mean temperature, food availability, and population density. Monthly mean temperature was categorized as “temperature” in the analyses. We calculated λ as the dominant eigenvalue of the matrix model (which described the population dynamics over one month) for each perturbation scenario. We calculated uncertainties around λ for each perturbation scenario by resampling regression coefficients 100 times from the MCMC posteriors, and then recalculating λ. This was the only species where λ could not be calculated on an annual scale. We tested whether this affected our results by repeating the global GLMM excluding this species. Doing so did not change our results (see the R script on GitHub named MainAnalysis_without_RhabdomysPumilio.R). The authors of (12) performed standard sensitivity analyses of the population growth rate, λ, and our results on the strength of different drivers on population dynamics are in line with these previous analyses (see Fig. S29).

The Meerkat (*Suricata suricatta*). The study population of these small social mammals is located in the Kuruman River Reserve in South Africa (5). They reach sexual maturity at the age of one year[5]. The data and code were provided by the corresponding author of (5). The model used was a mass-stage-classified integral projection model[5]. The covariates in the vital-rate models included population density, interannual rainfall, and temperature deviations (from seasonal means). We classified rainfall as “rain” and temperature deviations as “temperature” for our GLMMs. We calculated λ as the dominant eigenvalue of the annual integral projection model for each perturbation scenario. We obtained the uncertainties around λ for each perturbation scenario using non-parametric bootstrapping to obtain variation in vital rate parameters (following the original study (5)). The authors of (5) performed standard sensitivity analyses of the population growth rate, λ, and our results on the strength of different vital rates on population dynamics are in line with these previous analyses (see Fig. S30).

The Arctic Fox (*Vulpes lagopus*). The study population of these abundant generalists and apex predators is located in Svalbard, Norway (66). They reach sexual maturity at the age of 0.8 years (66). The climate and population data, as well as the full code on the model was provided by the corresponding author of study (66). The authors of the study built a Bayesian integrated population model (66). The covariates used in the vital-rate models were sea ice extent, availability of reindeer carcasses, and goose population size. We classified sea ice extent as

"temperature" and the others as biotic factors. We calculated λ as the dominant eigenvalue of the matrix model for each perturbation scenario under two hunting scenarios (low vs high pressure), and then averaged the results. We calculated uncertainties around λ for each perturbation scenario by resampling regression coefficients 100 times from the MCMC posteriors, and then recalculating λ. The authors of (66) performed standard sensitivity analyses of the population growth rate, λ, and our results on the strength of different vital rates on population dynamics are in line with these previous analyses (see Fig. S31).

The European rabbit (*Oryctolagus cuniculus*). Parameters to run an individual-based model of rabbit population dynamics in Doñana Protected Area (southwestern Spain) were obtained from Tablado and co-authors (104). Rabbits are native to the Iberian Peninsula but their abundances have declined, including in Doñana, due to a combination of climate and land-use change and diseases. Rabbits are fast-lived and reach sexual maturity at around 4 months. The study by Tablado and co-authors complied demographic parameters from previous studies to develop a stochastic individual-based model (IBM). The main climatic variable in the model was mean monthly temperature, from which we obtained measures of food (or green pasture) availability and breeding season length. Population density was also considered as a covariate in vital-rate models. We ran the IBM for 16 years (1-month time steps), discarding the first 5 years to account for transient dynamics. λ was then determined by calculating the changes in abundance per year using the formula $\lambda = \text{mean}((N_{t+1}/N_t))$, where t = abundance in June (end of the breeding season). We ran the simulations 100 times to obtain the uncertainties around λ for each perturbation scenario.

Plants

Shrubs (*Cistus libanotis, Halimium halimifolium, Lavandula stoechas*). These three common shrub species are located in Doñana National Park in Spain. *C. libanotis* reaches sexual maturity (i.e., mean age at first flowering in plants) at the age of 4.6 years, *H. halimifolium* at the age of 5.8 years, and *L. stoechas* at the age of 3 years (62). The climate and population-size data, posterior samples, and code were provided by the authors, and the data and code for the population model for *Cistus libanotis* and *Halimium halimifolium*, can be found on the author's GitHub repository: https://github.com/MariaPaniw/shrub_forecast (62). The demography of the latter two shrub species was described by a three-stage life cycle, from which a matrix population

model was built (62). The covariates used in the vital-rate models were rainfall, inter- and intraspecific densities. For *Lavandula stoechas*, we expanded the population model develop in (62) using individual-based data collected 2019-2023. We fit an integral projection model for this species, with vital rates were parameterized as functions of seasonal temperature and rainfall (adult plant density was used as on offset in recruitment models only). We classified seasonal temperature and rainfall as "temperature" "rain" for our GLMMs, respectively. We calculated λ as the dominant eigenvalue of the matrix model or integral projection model for each perturbation scenario. We calculated uncertainties around λ for each perturbation scenario by resampling regression coefficients 100 times from the MCMC posteriors (or multivariate Normal distribution based on the parameter covariance matrix for *L. stoechas*), and then recalculating λ. The authors of (62) performed standard sensitivity analyses of the population growth rate, λ, and our results on the strength of different vital rates on population dynamics are in line with these previous analyses (see Figs. S33 and S35).

<u>The Pontic Dragonhead (*Dracocephalum austriacum*).</u> The four study populations are located in the Bohemian Karst in Central Europe (105). This study species reaches its sexual maturity at the age of two on average[105]. The data and code were provided by the corresponding author (Evers et al. in preparation). They built an integral projection model. The climatic covariates used in the vital-rate models were potential evapotranspiration (PET), precipitation, and temperature. We classified precipitation as a climatic driver "rain", and temperature as "temperature" for our GLMMs. We calculated λ as the dominant eigenvalue of the matrix model for each perturbation scenario. We obtained uncertainties around λ for each perturbation scenario by resampling coefficients 100 times from the functional linear models, which linked climate drivers to vital rates, and then recalculating λ.

<u>The Dewy Pine (*Drosophyllum lusitanicum*).</u> The eight study populations of the dewy pine, a carnivorous subshrub, are located in the heathlands of Southern Spain (94, 106). The dewy pine reaches sexual maturity at the age of two years (106). The data and code for the analysis was provided by the corresponding author (94). They built an individual-based model (IBM) and used these five covariates in the vital-rate models: temperature, rainfall, density, size, and time since last fire. The two climatic drivers, temperature and rainfall, were classified as such. We calculated λ by projecting the population for 50 years, discarding the first 25 years to account for transient dynamics. λ was then determined by calculating the changes in abundance per year using the

formula λ = (N_{t+1}/N_t) and averaging it over all the years. We calculated uncertainties around λ for each perturbation scenario by running the simulation 100 times, and then recalculating λ.

Spanish Trees. This study includes several tree species populations across SpaiN (40). These include *Fagus sylvatica*, *Pinus halepensis*, *Pinus nigra*, *Pinus pinaster*, *Pinus pinea*, *Pinus sylvestris*, *Pinus uncinata*, *Quercus faginea*, *Quercus ilex*, *Quercus robur/petraea*, and *Quercus suber*. The mean age at sexual maturity of each species can be found in Table S7. The climate data and the code for the model were obtained from the corresponding author's GitHub repository: https://github.com/garciacallejas/IPM_basic (40). A spatially explicit integral projection model was constructed for all tree species (40). The covariates in the vital-rate models include temperature, precipitation, and their anomalies. We classified temperature and precipitation as "temperature" and "rain" for our analyses. We calculated λ by running the model for 90 years (10-year time steps), discarding the first 50 years to account for transient dynamics. λ was then determined by calculating the changes in abundance per year using the formula λ = (N_{t+1}/N_t) and averaging it. We calculated uncertainties around λ for each perturbation scenario by running the simulation five times (due to computational demands), and then recalculating λ. In the original study, the tree species were spread across the continental territory of Spain in a 1 km x 1 km grid system (40). For our study, we first calculated the scaled sensitivities and then averaged across the area.

The Tree Cholla Cactus (*Opuntia imbricata*). The study population is located at the Sevilleta National Wildlife Refuge in New Mexico, USA (107, 108). This species reaches sexual maturity at the age of nine years (109). The integral projection model is based on the R script from Aldo Compagnoni (110). Further, two vital-rate models are from the analysis of Sanne Evers (109). The data and code were provided by the corresponding author of (109). The covariates in the vital-rate models were climate anomalies of the values instead of absolute values. The variables were mean average daily temperatures of two different time windows and mean minimum daily temperature (109). We classified the covariates in our analyses as "temperature". We calculated λ as the dominant eigenvalue of the matrix for each perturbation scenario. We calculated uncertainties around λ for each perturbation scenario by resampling regression coefficients 100 times from the posterior distributions, and then recalculating λ.

The Common Sugarbush (*Protea repens*). This species of shrub is found throughout the Mediterranean climate of the Cape Floristic Region in South Africa (111). It reaches sexual maturity at the age of four years (112). All data and code were accessible online (111). However, to obtain posterior samples of regression coefficients, we rebuilt and executed the regression models in JAGS (113). The population model built is an integral projection model. The covariates used in the vital-rate models that we perturbed were minimum July temperature and mean annual precipitation, respectively classified as “temperature” and “rain” in the meta-regressions. We calculated λ as the dominant eigenvalue of the matrix model for each perturbation scenario. We calculated uncertainties around λ for each perturbation scenario by resampling regression coefficients 100 times from the posterior distributions, and then recalculating λ.

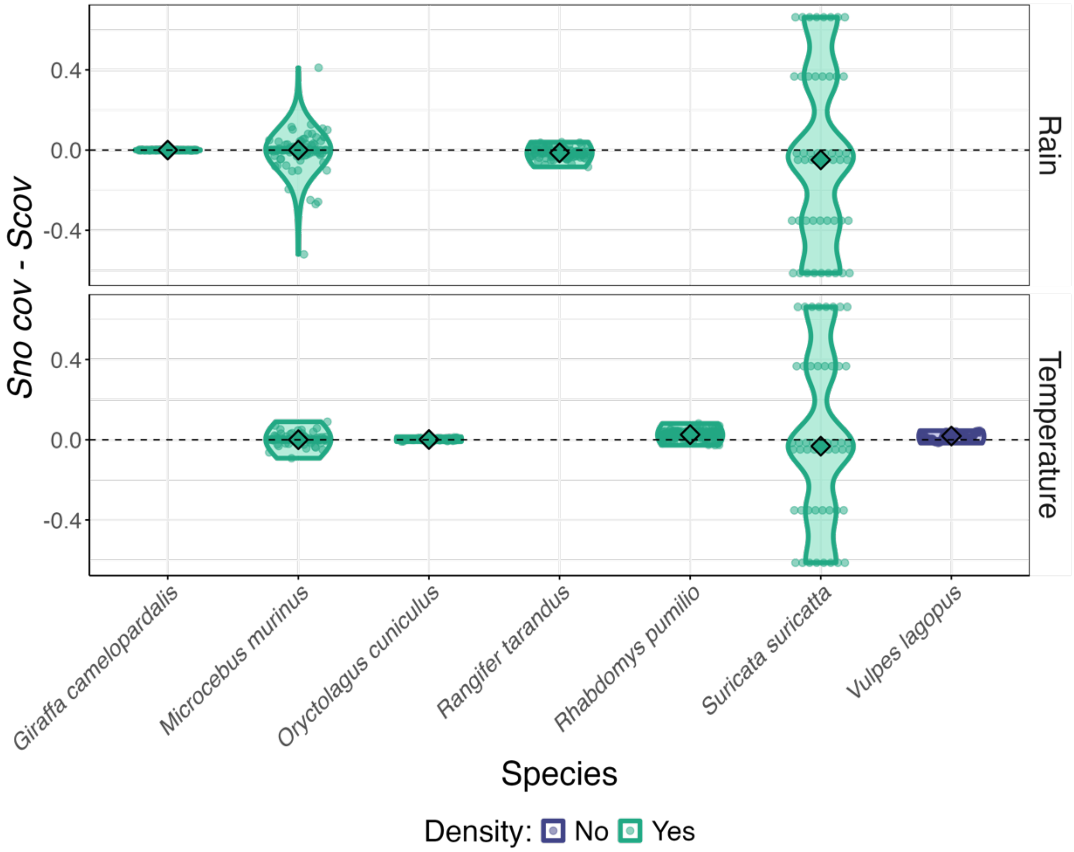

Figure S9. Differences in scaled sensitivities |S| of population growth rates of mammalian species to (A) rain and (B) temperature, without and with covarying drivers ($S_{no\ cov} - S_{cov}$). Different colors indicate models where density effects were included or not. A positive difference indicates that the sensitivities with covariation are lower than those without covariation, implying that there are dampening effects of covariation on the sensitivity of a species. The diamond symbols display the median sensitivities, while the points represent all calculated sensitivities from 100 resamplings per species ($n_{resamplings}$ = 100).

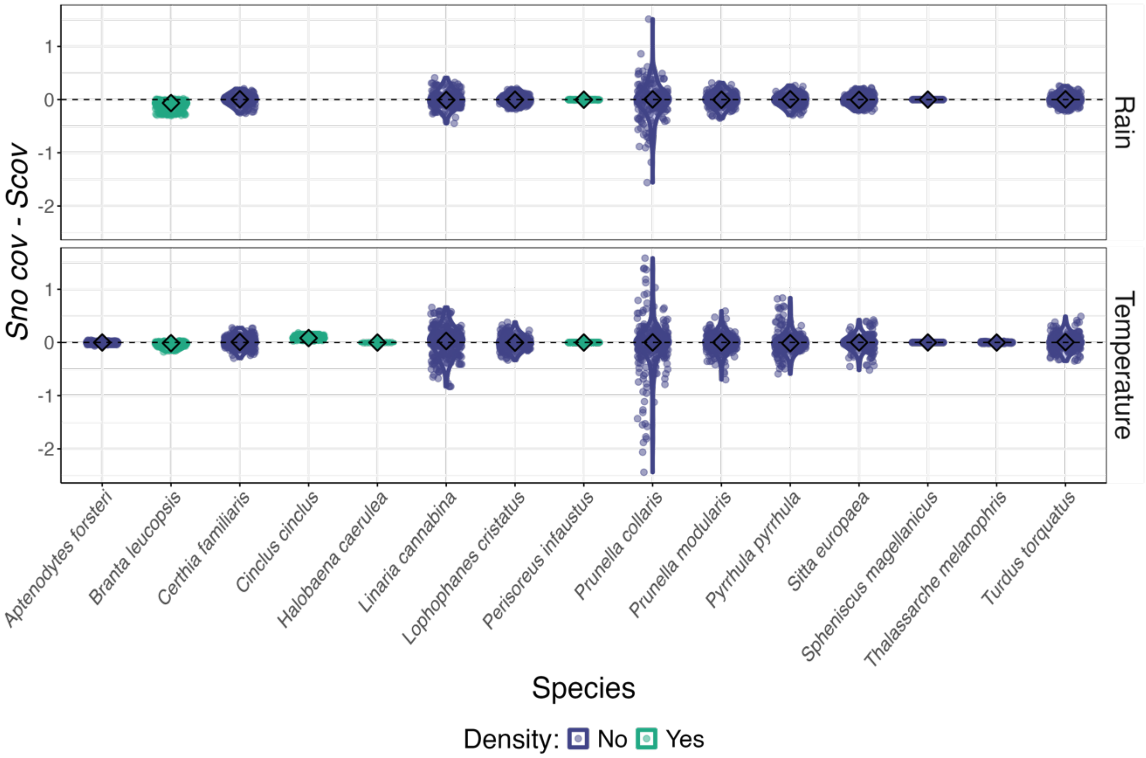

Figure S10. Differences in scaled sensitivities |S| of population growth rates of bird species to (A) rain and (B) temperature, without and with covarying drivers ($S_{no\ cov} – S_{cov}$). Different colors indicate models where density effects were included or not. A positive difference indicates that the sensitivities with covariation are lower than those without covariation, implying that there are dampening effects of covariation on the sensitivity of a species. The diamond symbols display the median sensitivities, while the points represent all calculated sensitivities from 100 resamplings per species ($n_{resamplings} = 100$, $n_{resamplings\ for\ Halobaena\ caerulea} = 50$).

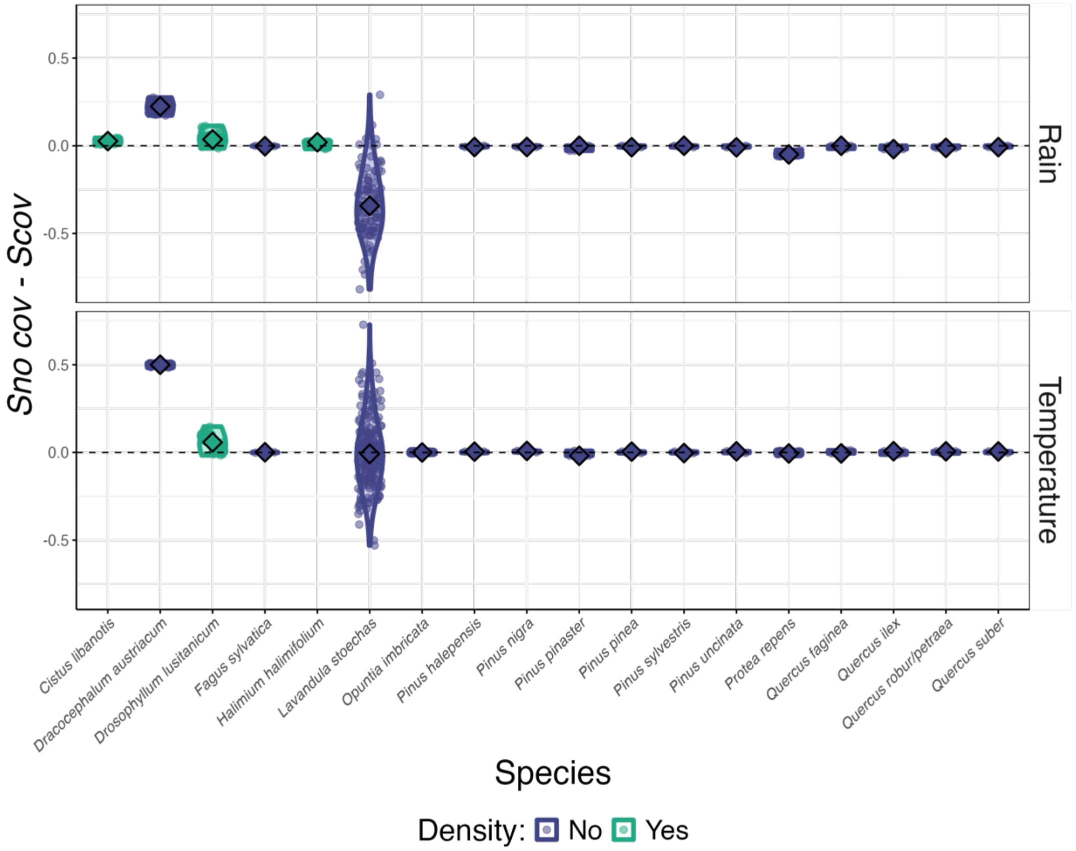

Figure S11. Differences in scaled sensitivities |S| of population growth rates of plant species to (A) rain and (B) temperature, without and with covarying drivers ($S_{no\ cov} - S_{cov}$). Different colors indicate models where density effects were included or not. A positive difference indicates that the sensitivities with covariation are lower than those without covariation, implying that there are dampening effects of covariation on the sensitivity of a species. The diamond symbols display the median sensitivities, while the points represent all calculated sensitivities from 100 resamplings per species ($n_{resamplings}$ = 100, $n_{resamplings\ for\ Spanish\ Trees}$ = 5).

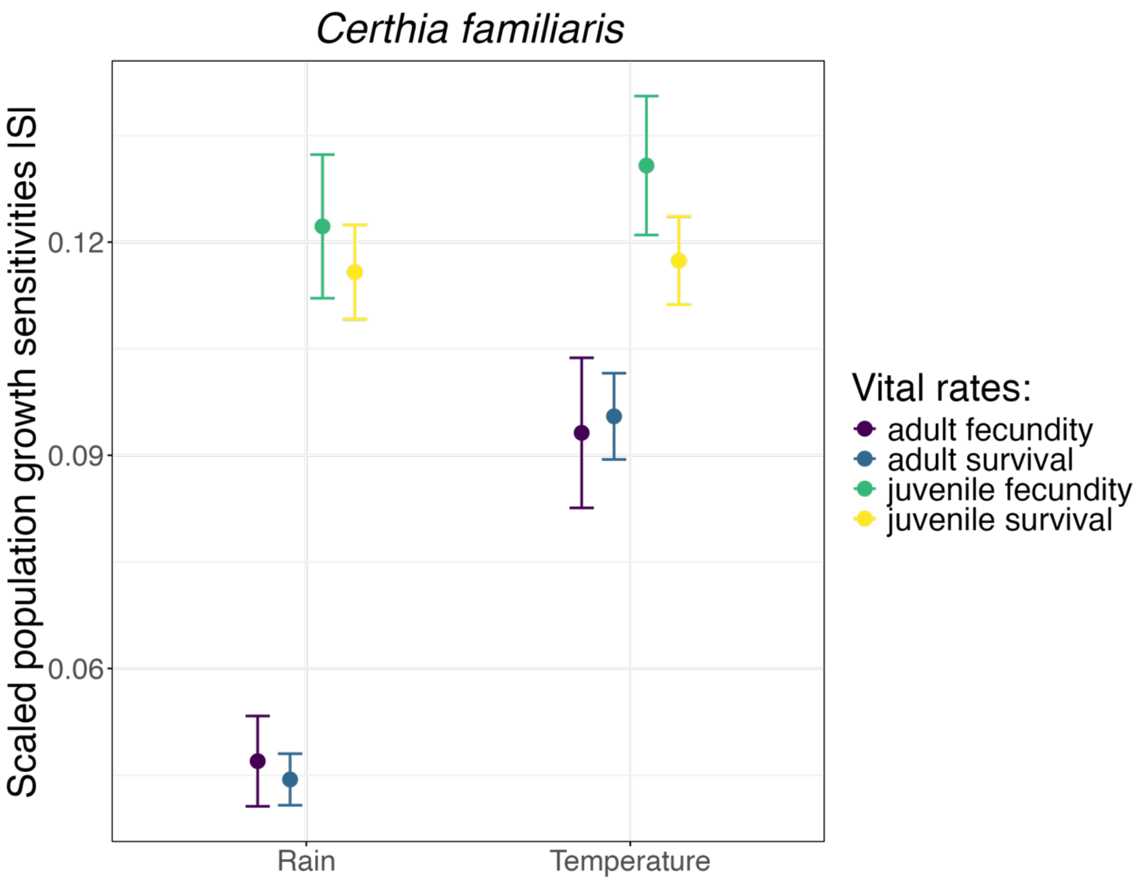

Figure S12. Scaled sensitivities of population growth rates per vital rate to different drivers for *Certhia familiaris*. The dots represent the mean scaled sensitivities across the calculated resamplings per driver and vital rate combination ($n_{resamplings}$ = 100) and the error bars display the standard errors. The climatic drivers here are rain and temperature.

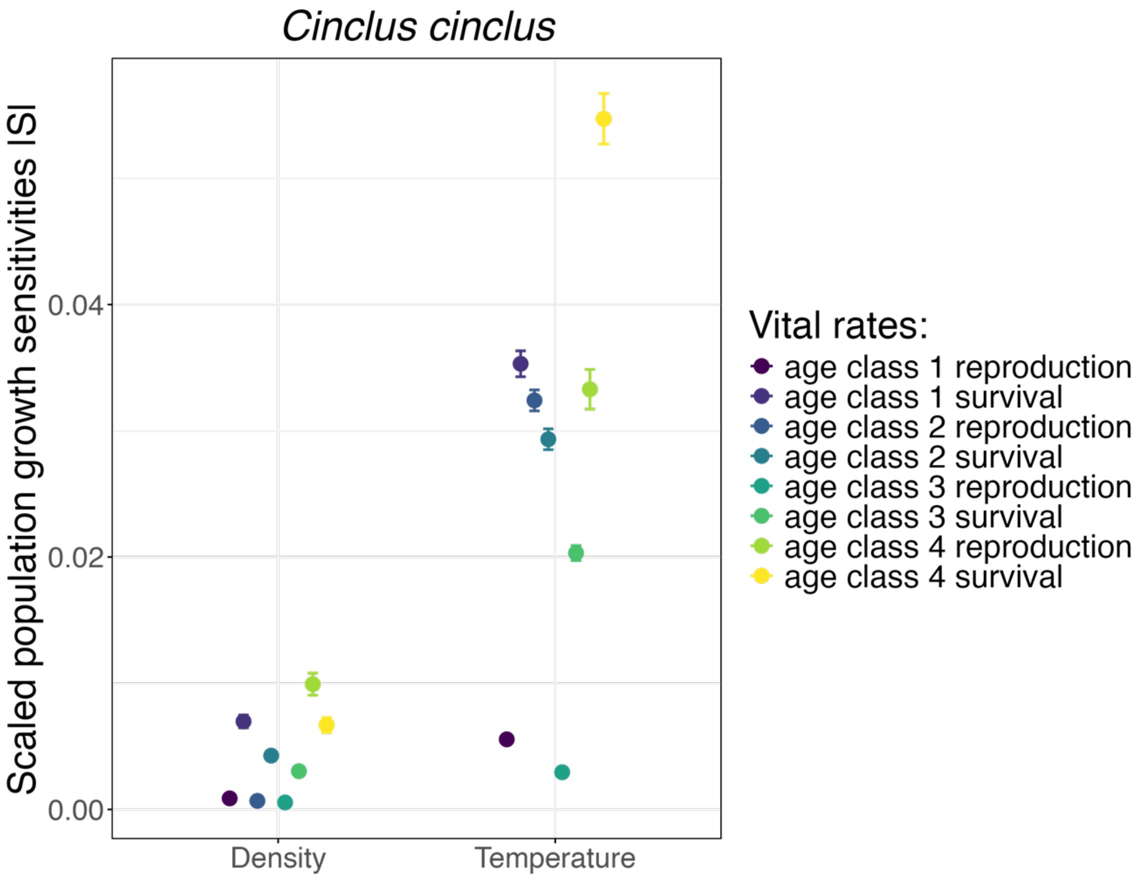

Figure S13. Scaled sensitivities of population growth rates per vital rate to different drivers for *Cinclus* cinclus. The dots represent the mean scaled sensitivities across the calculated resamplings per driver and vital rate combination ($n_{resamplings}$ = 100) and the error bars display the standard errors. The climatic driver here is temperature, and the density driver refers to intraspecific density. In (36), sensitivities of λ to temperature and density were calculated at equilibrium population density and also show that sensitivities to temperatures are more variable than to density (see Fig. 3 in (36))

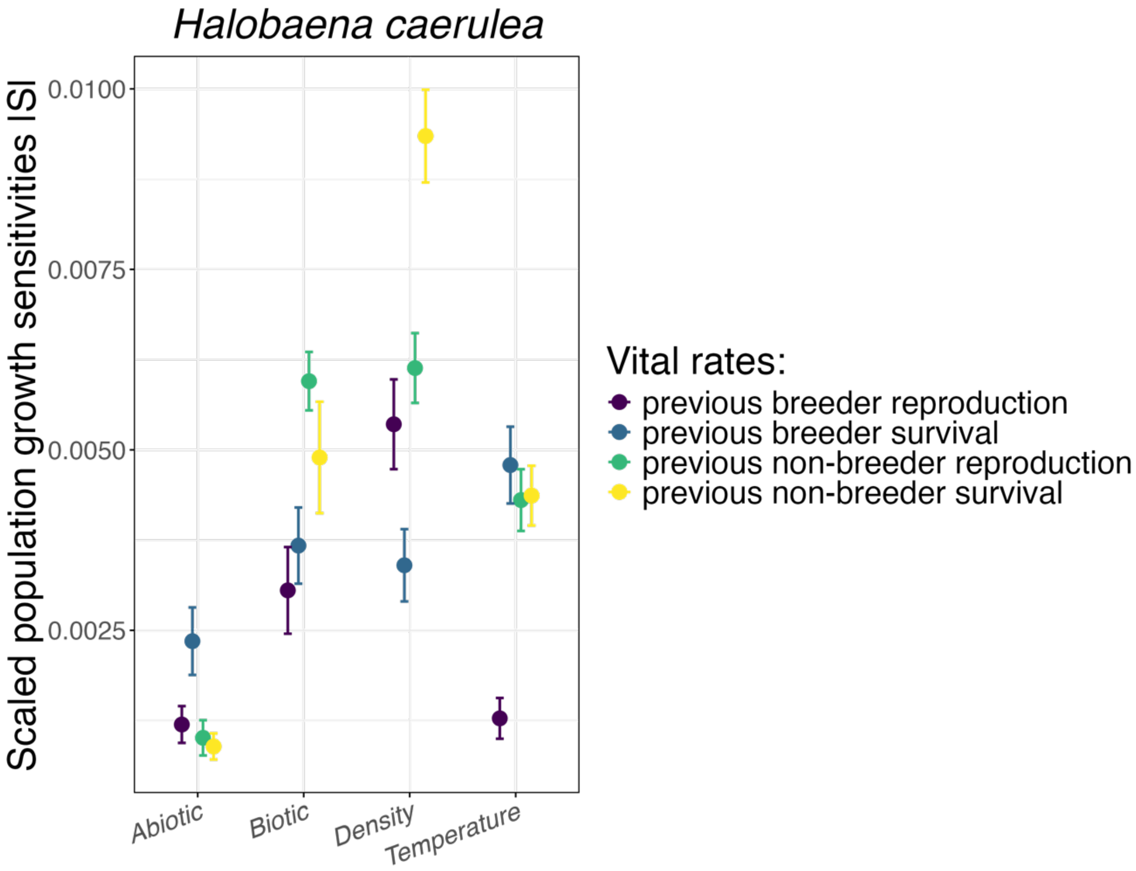

Figure S14. Scaled sensitivities of population growth rates per vital rate to different drivers for *Halobaena caerulea*. The dots represent the mean scaled sensitivities across the calculated resamplings per driver and vital rate combination ($n_{resamplings}$ = 50) and the error bars display the standard errors. The abiotic driver here is the Southern Annular Mode, the biotic driver is interspecific density, the density driver refers to intraspecific density, and the climatic driver is sea surface temperature.

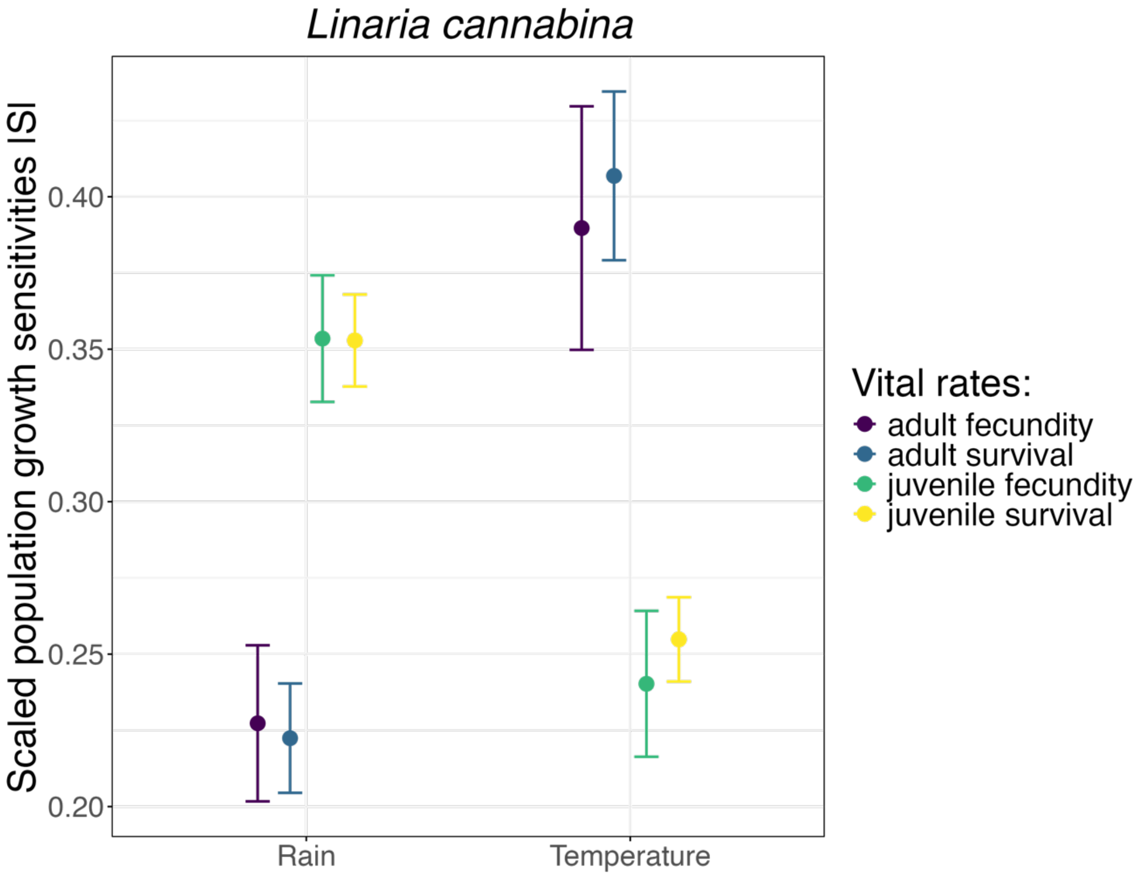

Figure S15. Scaled sensitivities of population growth rates per vital rate to different drivers for *Linaria cannabina*. The dots represent the mean scaled sensitivities across the calculated resamplings per driver and vital rate combination ($n_{resamplings}$ = 100) and the error bars display the standard errors. The climatic drivers here are rain and temperature.

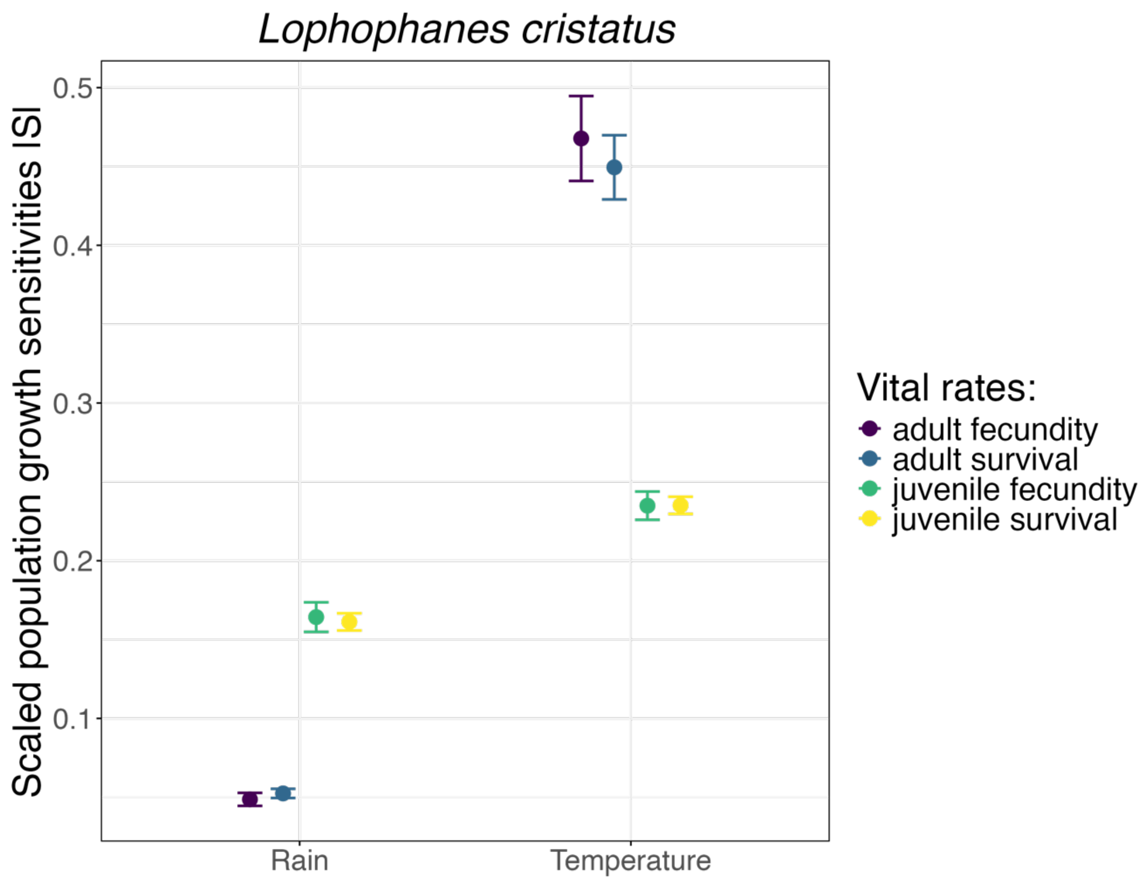

Figure S16. Scaled sensitivities of population growth rates per vital rate to different drivers for *Lophophanes cristatus*. The dots represent the mean scaled sensitivities across the calculated resamplings per driver and vital rate combination ($n_{resamplings}$ = 100) and the error bars display the standard errors. The climatic drivers here are rain and temperature.

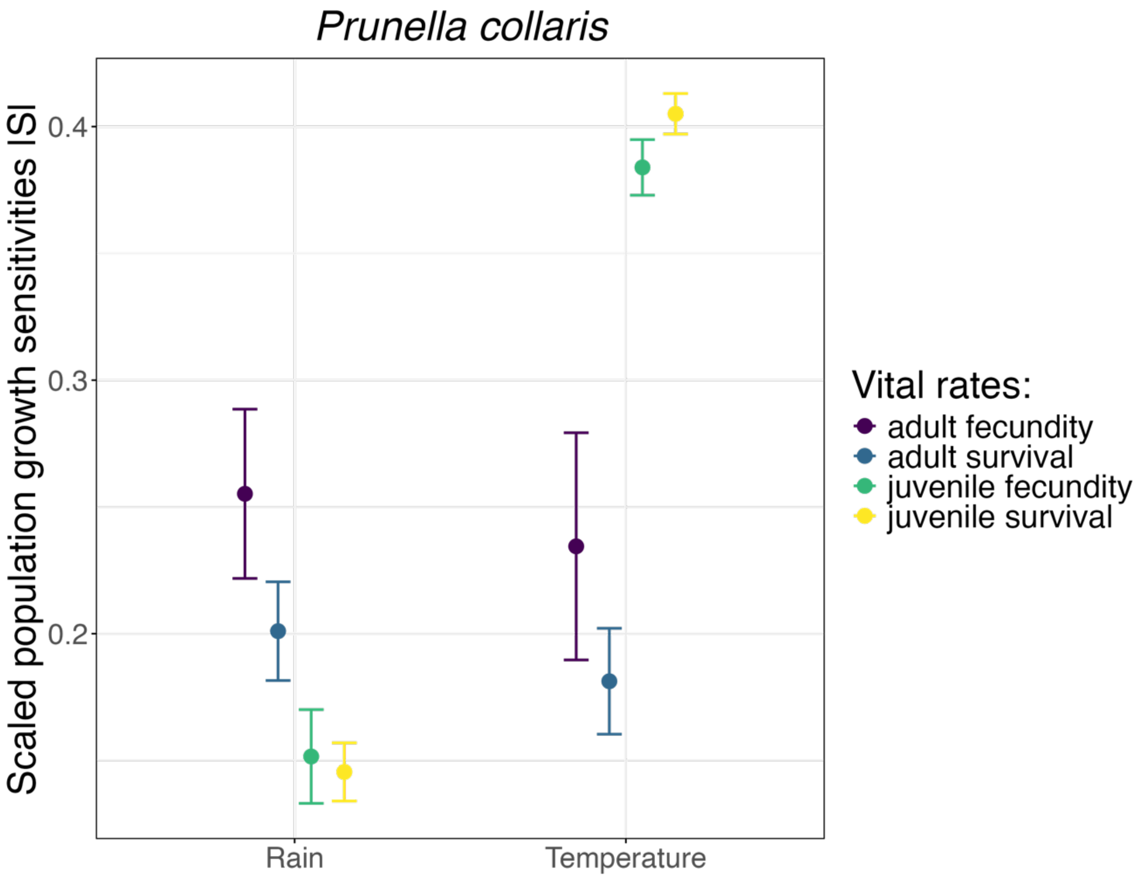

Figure S17. Scaled sensitivities of population growth rates per vital rate to different drivers for *Prunella collaris*. The dots represent the mean scaled sensitivities across the calculated resamplings per driver and vital rate combination ($n_{resamplings}$ = 100) and the error bars display the standard errors. The climatic drivers here are rain and temperature.

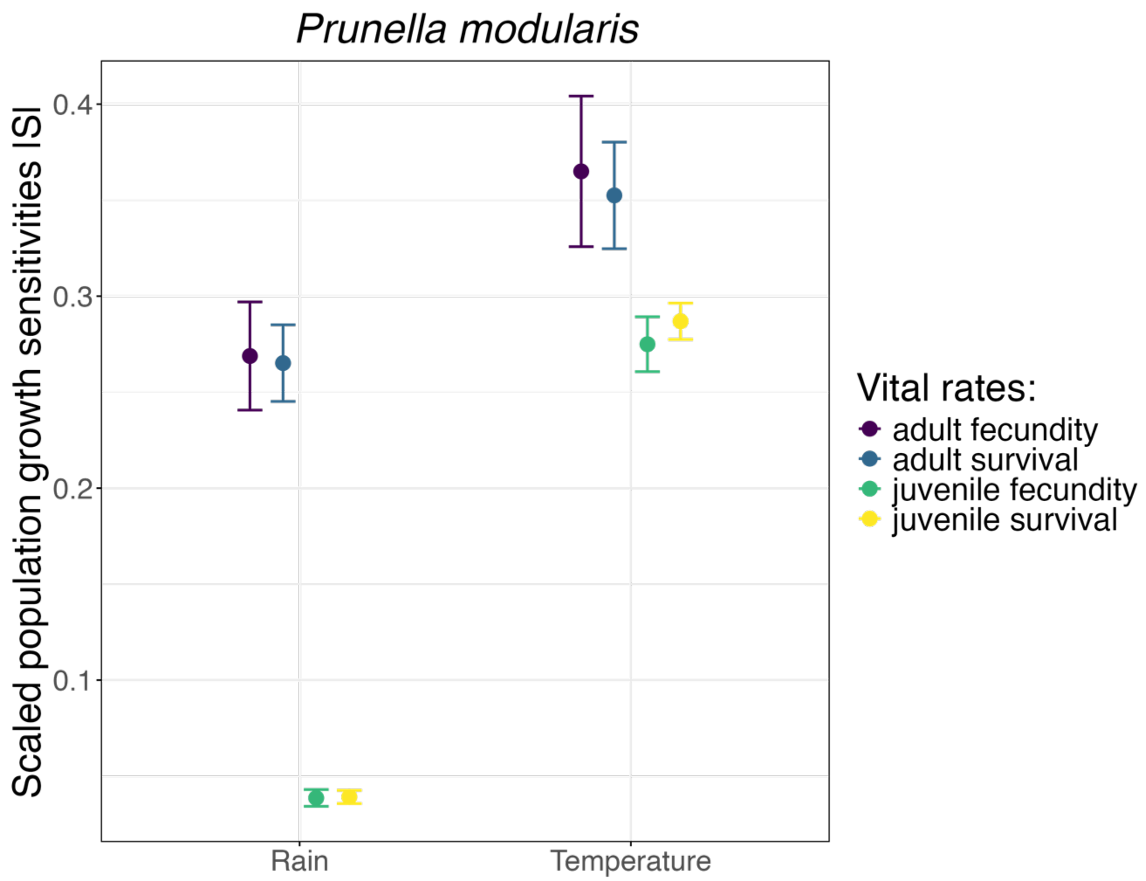

Figure S18. Scaled sensitivities of population growth rates per vital rate to different drivers for *Prunella modularis*. The dots represent the mean scaled sensitivities across the calculated resamplings per driver and vital rate combination ($n_{resamplings}$ = 100) and the error bars display the standard errors. The climatic drivers here are rain and temperature.

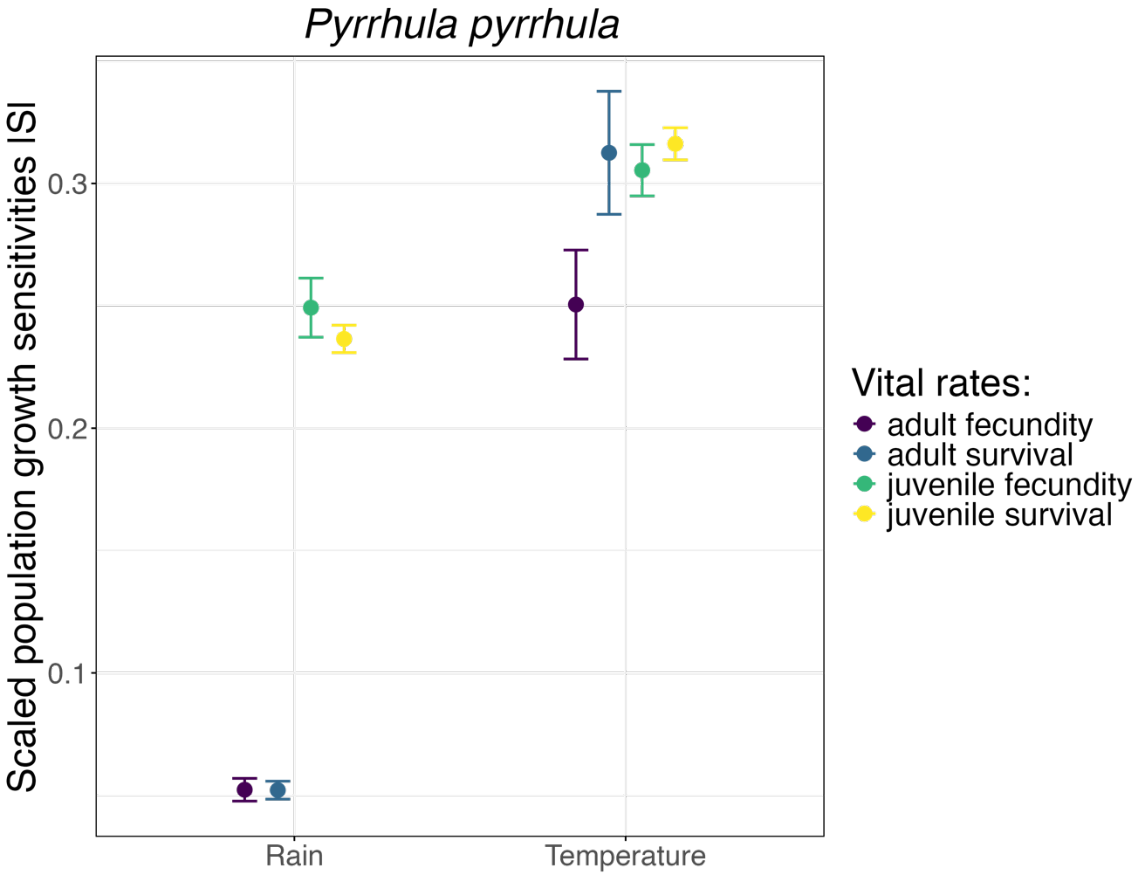

Figure S19. Scaled sensitivities of population growth rates per vital rate to different drivers for *Pyrrhula pyrrhula*. The dots represent the mean scaled sensitivities across the calculated resamplings per driver and vital rate combination ($n_{resamplings}$ = 100) and the error bars display the standard errors. The climatic drivers here are rain and temperature.

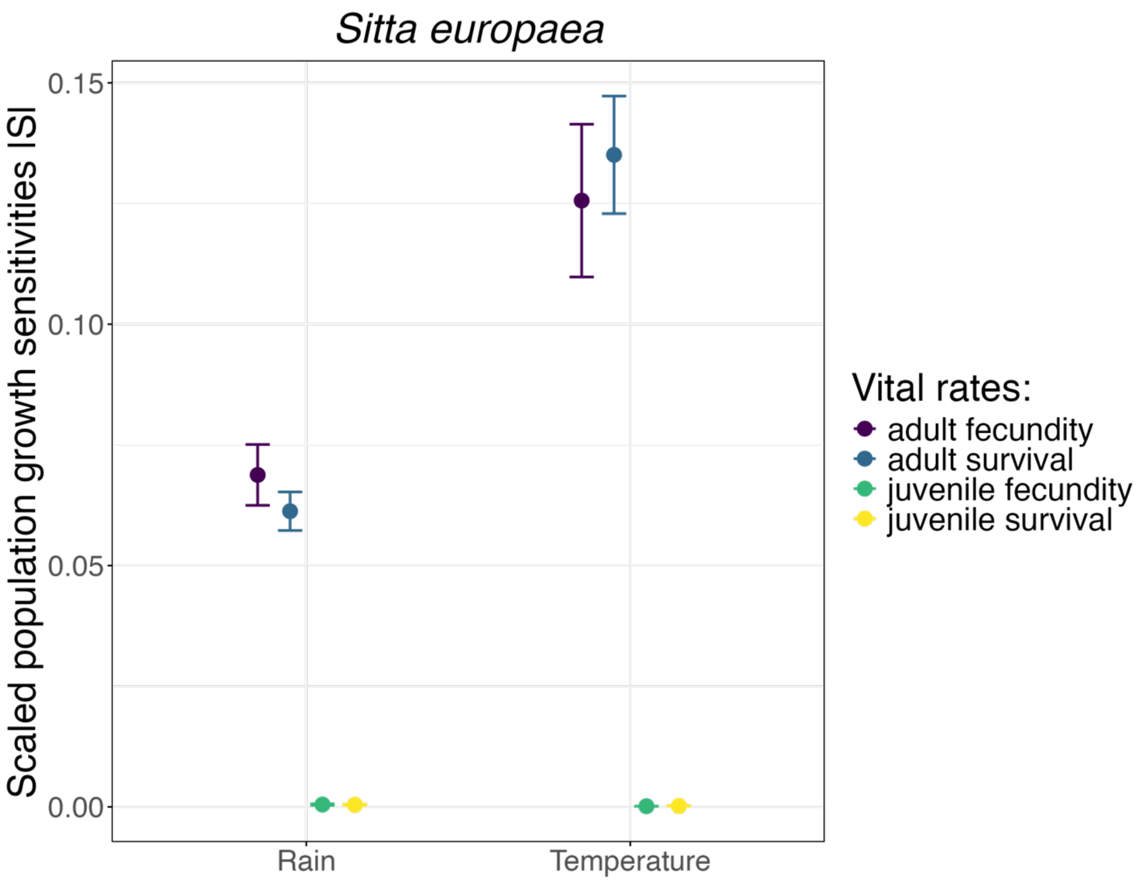

Figure S20. Scaled sensitivities of population growth rates per vital rate to different drivers for *Sitta europaea*. The dots represent the mean scaled sensitivities across the calculated resamplings per driver and vital rate combination ($n_{resamplings}$ = 100) and the error bars display the standard errors. The climatic drivers here are rain and temperature.

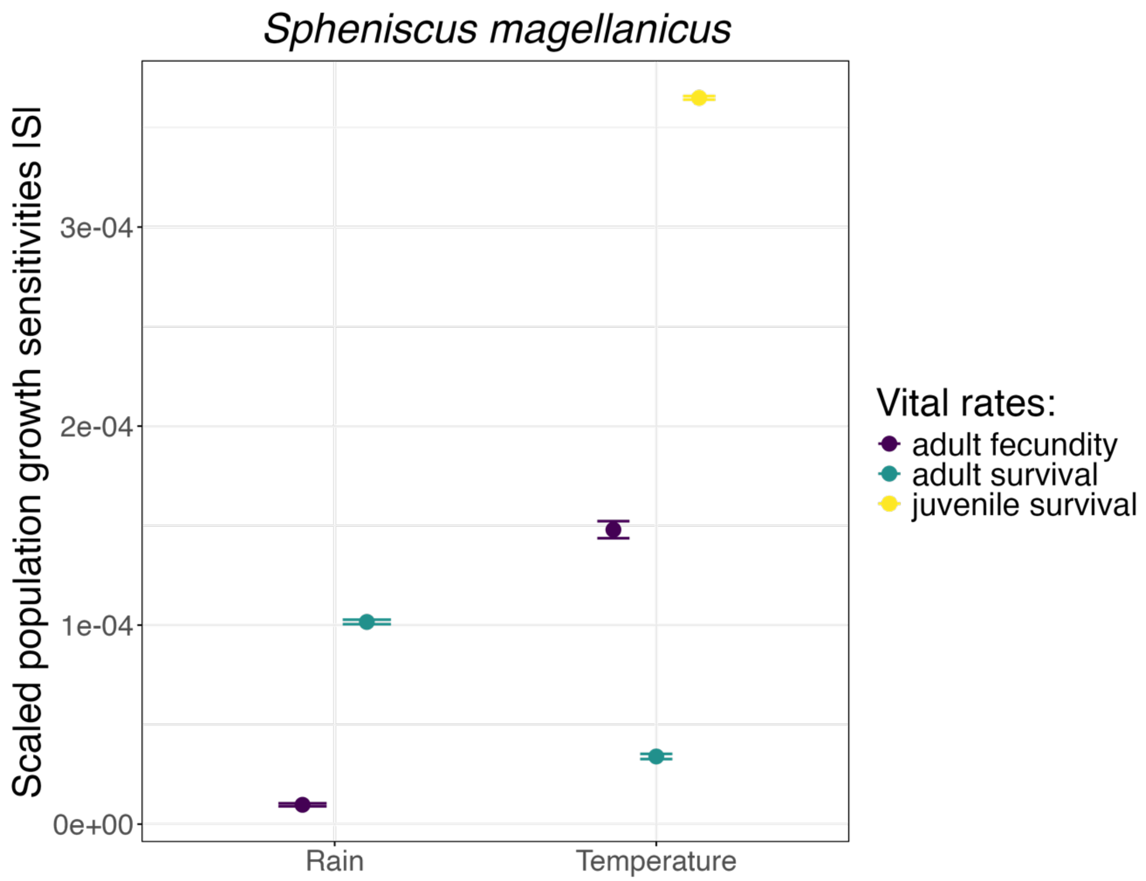

Figure S21. Scaled sensitivities of population growth rates per vital rate to different drivers for *Spheniscus magellanicus*. The dots represent the mean scaled sensitivities across the calculated resamplings per driver and vital rate combination ($n_{resamplings}$ = 100) and the error bars display the standard errors. The climatic drivers here are rain, temperature, and sea surface temperature anomalies (also classified as temperature).

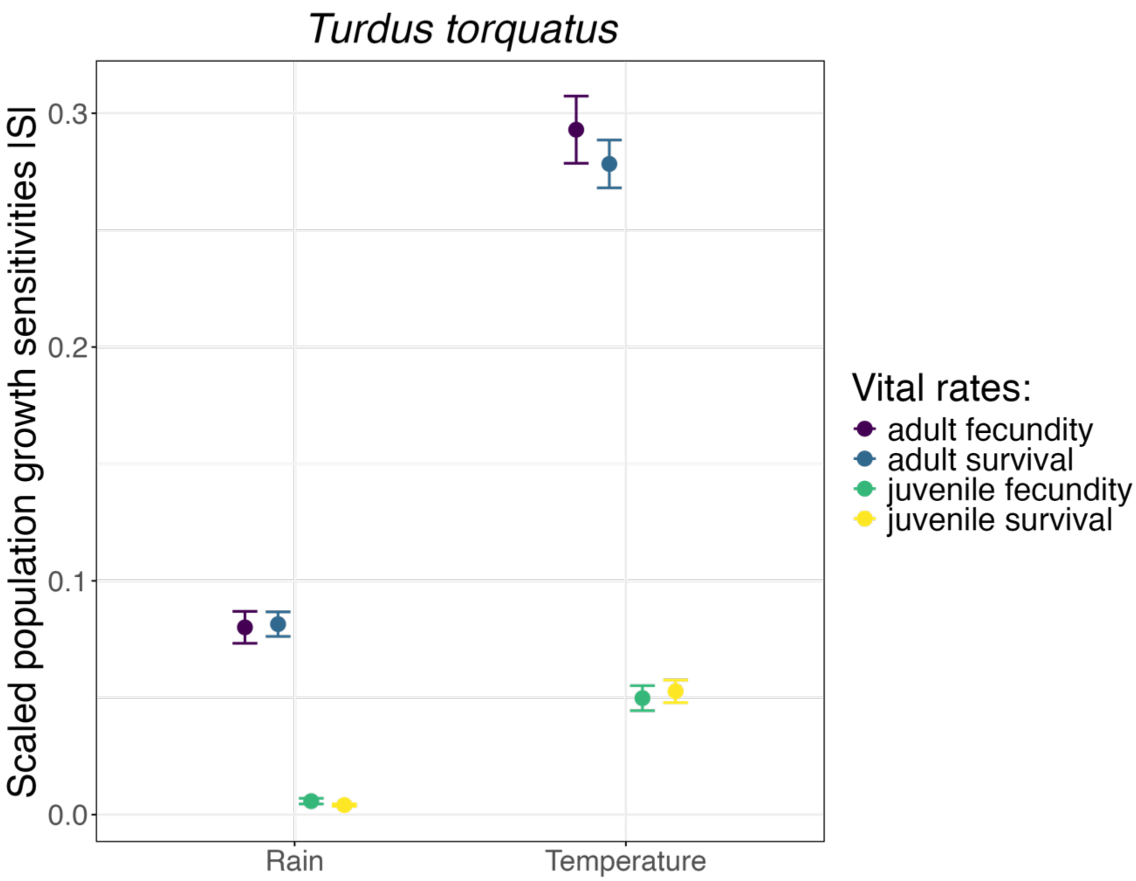

Figure S22. Scaled sensitivities of population growth rates per vital rate to different drivers for *Turdus torquatus*. The dots represent the mean scaled sensitivities across the calculated resamplings per driver and vital rate combination ($n_{resamplings}$ = 100) and the error bars display the standard errors. The climatic drivers here are rain and temperature.

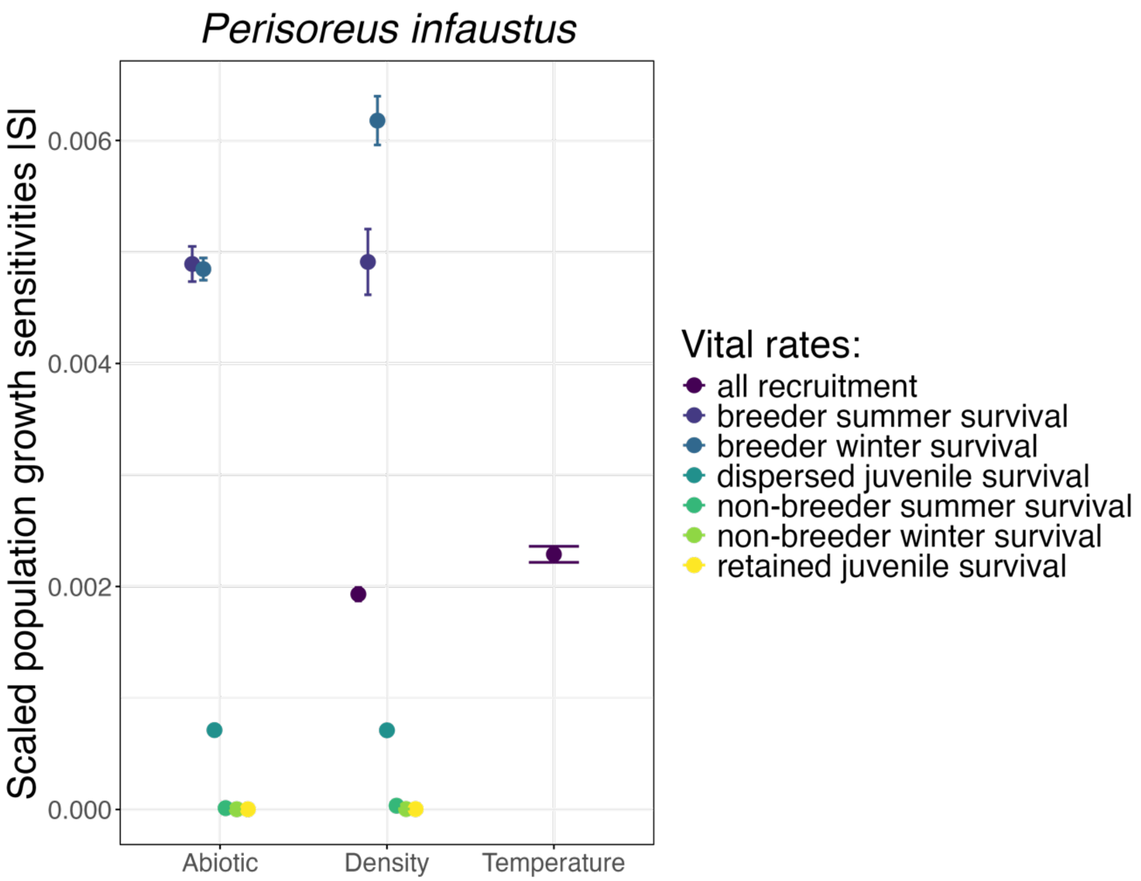

Figure S23. Scaled sensitivities of population growth rates per vital rate to different drivers for *Perisoreus infaustus*. The dots represent the mean scaled sensitivities across the calculated resamplings per driver and vital rate combination ($n_{resamplings}$ = 100) and the error bars display the standard errors. The climatic drivers here are rain and temperature. In (93), relative sensitivities of λ to different vital rates were calculated at equilibrium population density and also show that λ is most sensitive to breeder summer and winter survival (see Fig. 3 in (93)). In addition, our analyses are in line with results from (93) showing that recruitment is strongly affected by both density and temperature (Fig. 4 in (93)).

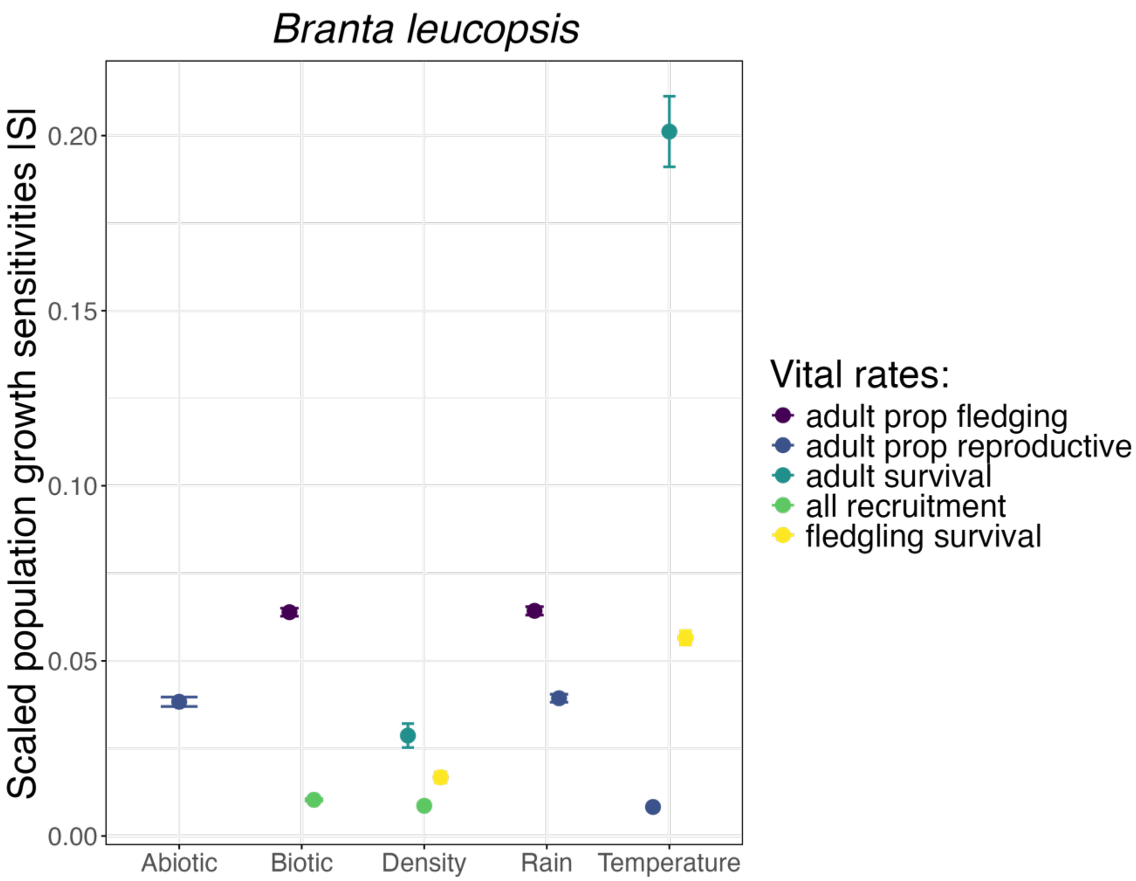

Figure S24. Scaled sensitivities of population growth rates per vital rate to different drivers for *Branta leucopsis*. The dots represent the mean scaled sensitivities across the calculated resamplings per driver and vital rate combination ($n_{resamplings}$ = 100) and the error bars display the standard errors. The climatic drivers here are rain and temperature. In (63), sensitivities of λ to different environmental variables affecting different vital rates were calculated at equilibrium population density and show that temperature and rainfall more strongly affect λ than intraspecific density and that these affects were strongest when adult survival and reproduction was perturbed (see Fig. 6 in (63)).

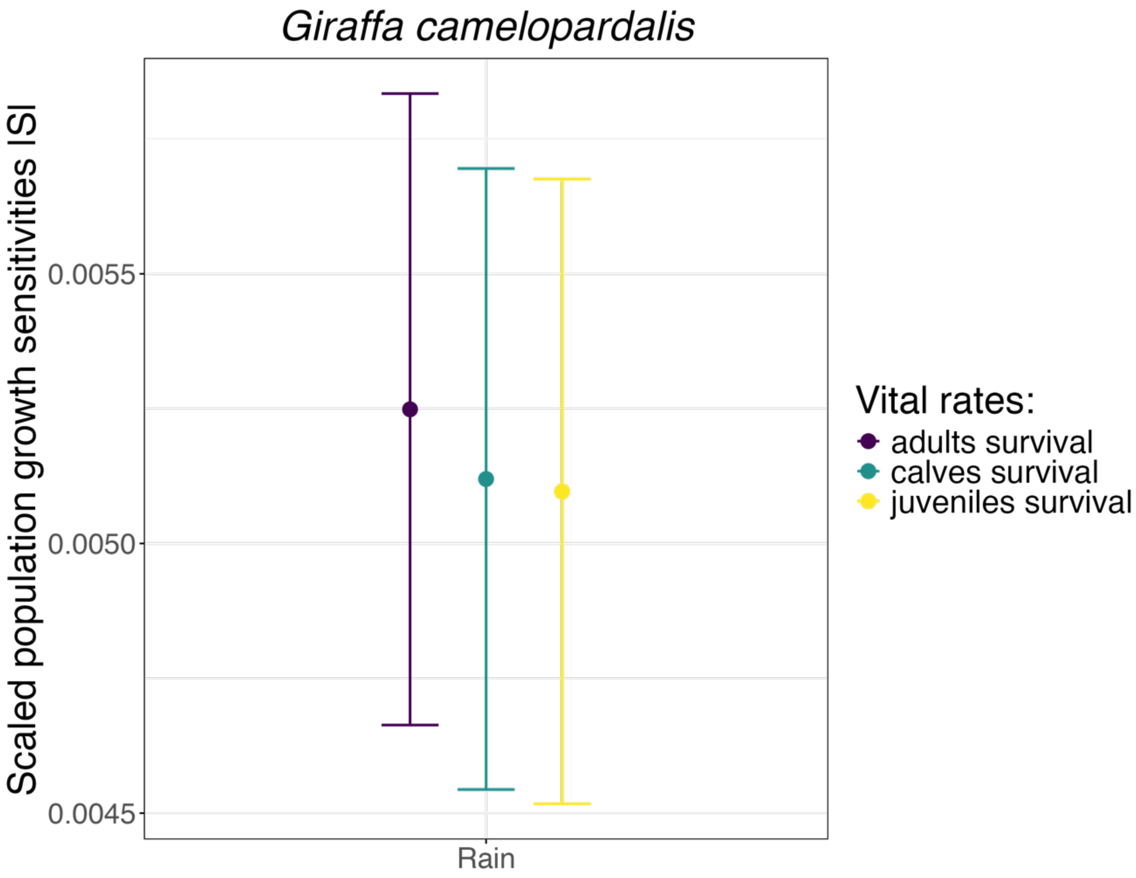

Figure S25. Scaled sensitivities of population growth rates per vital rate to different drivers for *Giraffa camelopardalis*. The dots represent the mean scaled sensitivities across the calculated resamplings per driver and vital rate combination ($n_{resamplings}$ = 100) and the error bars display the standard errors. The climatic driver here is rain.

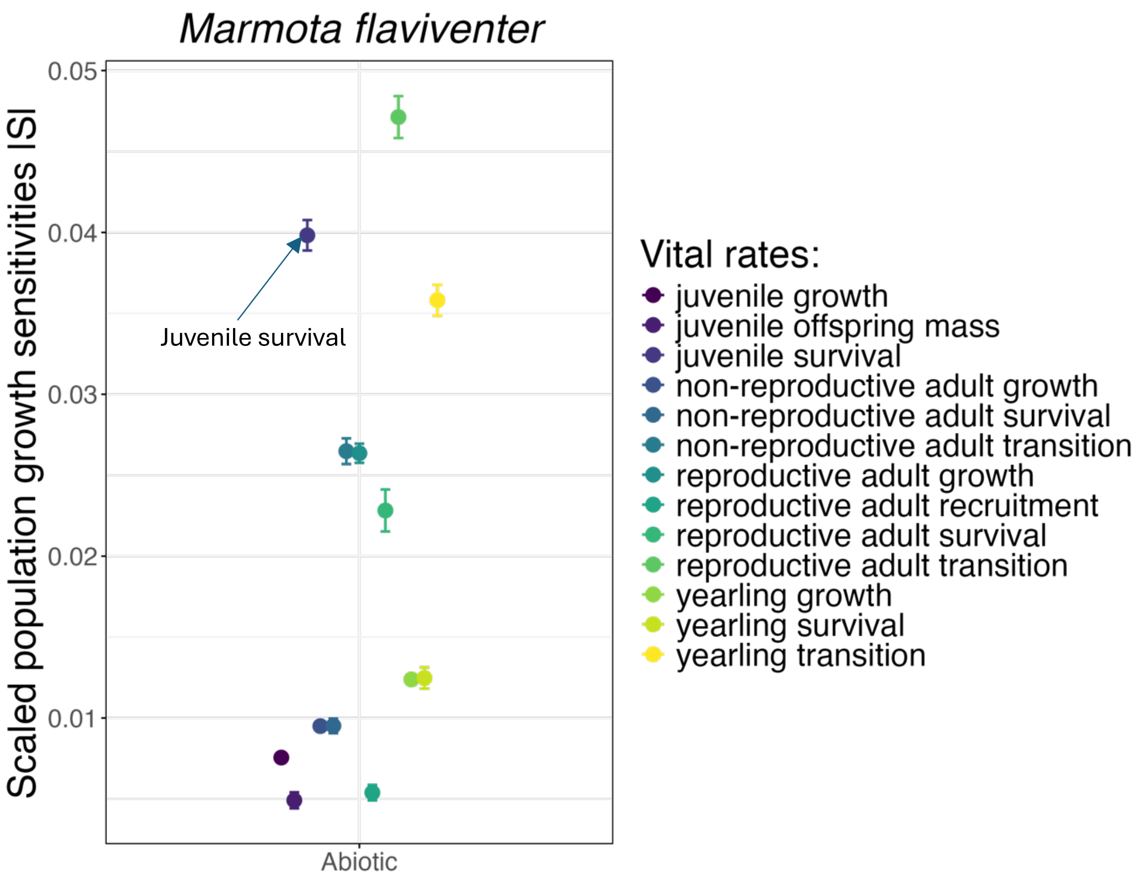

Figure S26. Scaled sensitivities of population growth rates per vital rate to different drivers for *Marmota flaviventer*. The dots represent the mean scaled sensitivities across the calculated resamplings per driver and vital rate combination ($n_{resamplings}$ = 100) and the error bars display the standard errors. The abiotic driver here is Q, which is a composite measure, including climate, representing environmental quality. In (99), sensitivities of stochastic λ to Q affecting different vital rates were calculated and show that Q most strongly impacts stochastic λ through its effect on reproduction, i.e., transition to reproductive here, and juvenile survival (see Fig. 2 in (99)).

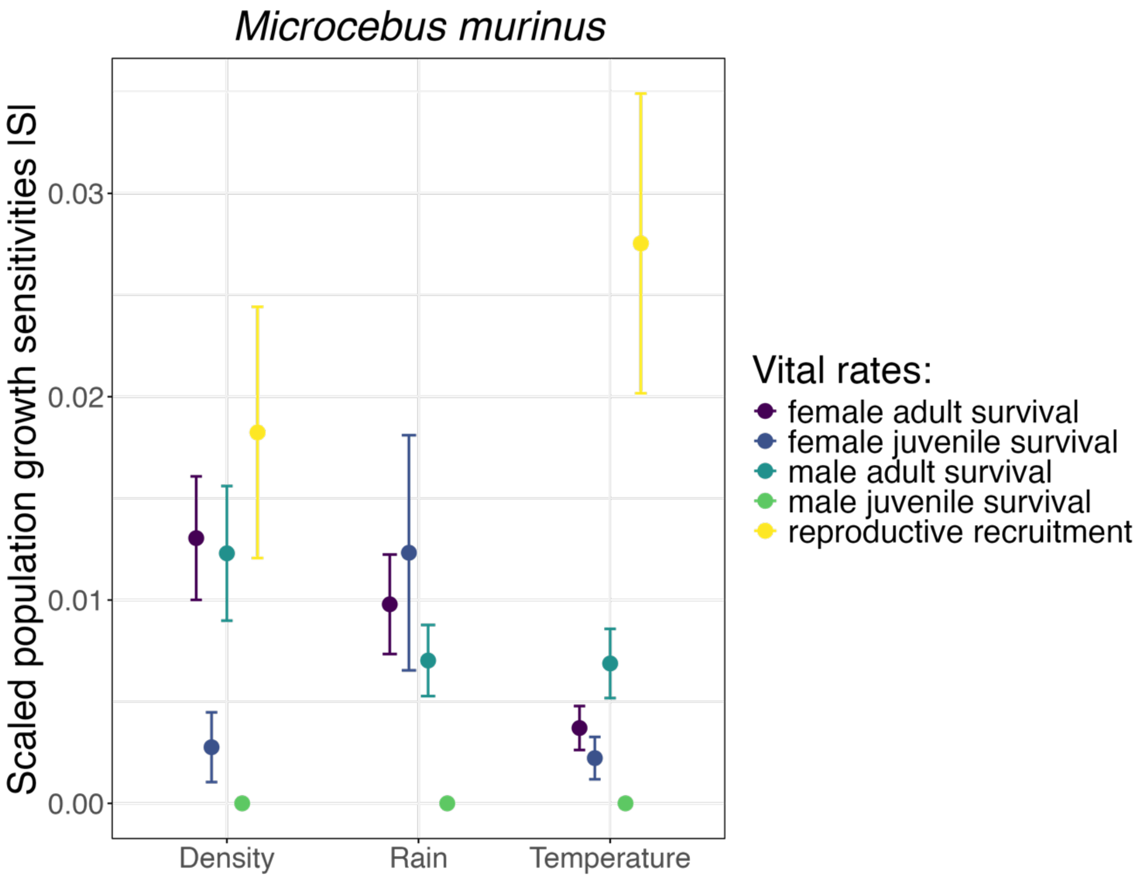

Figure S27. Scaled sensitivities of population growth rates per vital rate to different drivers for *Microcebus murinus*. The dots represent the mean scaled sensitivities across the calculated resamplings per driver and vital rate combination ($n_{resamplings}$ = 100) and the error bars display the standard errors. The density driver refers to intraspecific density, and the climatic drivers are rain and temperature.

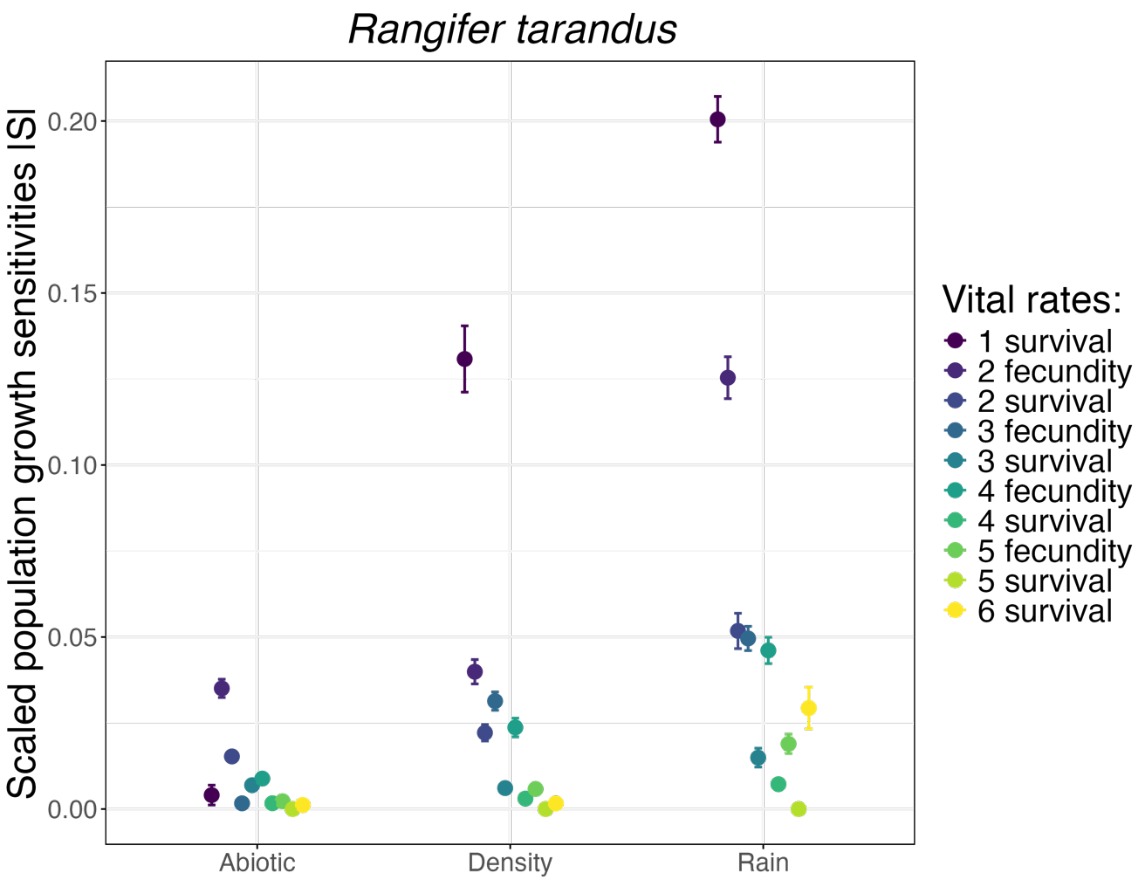

Figure S28. Scaled sensitivities of population growth rates per vital rate to different drivers for *Rangifer tarandus*. The dots represent the mean scaled sensitivities across the calculated resamplings per driver and vital rate combination ($n_{resamplings}$ = 100) and the error bars display the standard errors. The abiotic driver here is winter length, the density driver refers to intraspecific density, and the climatic driver is rain-on-snow, classified as rain.

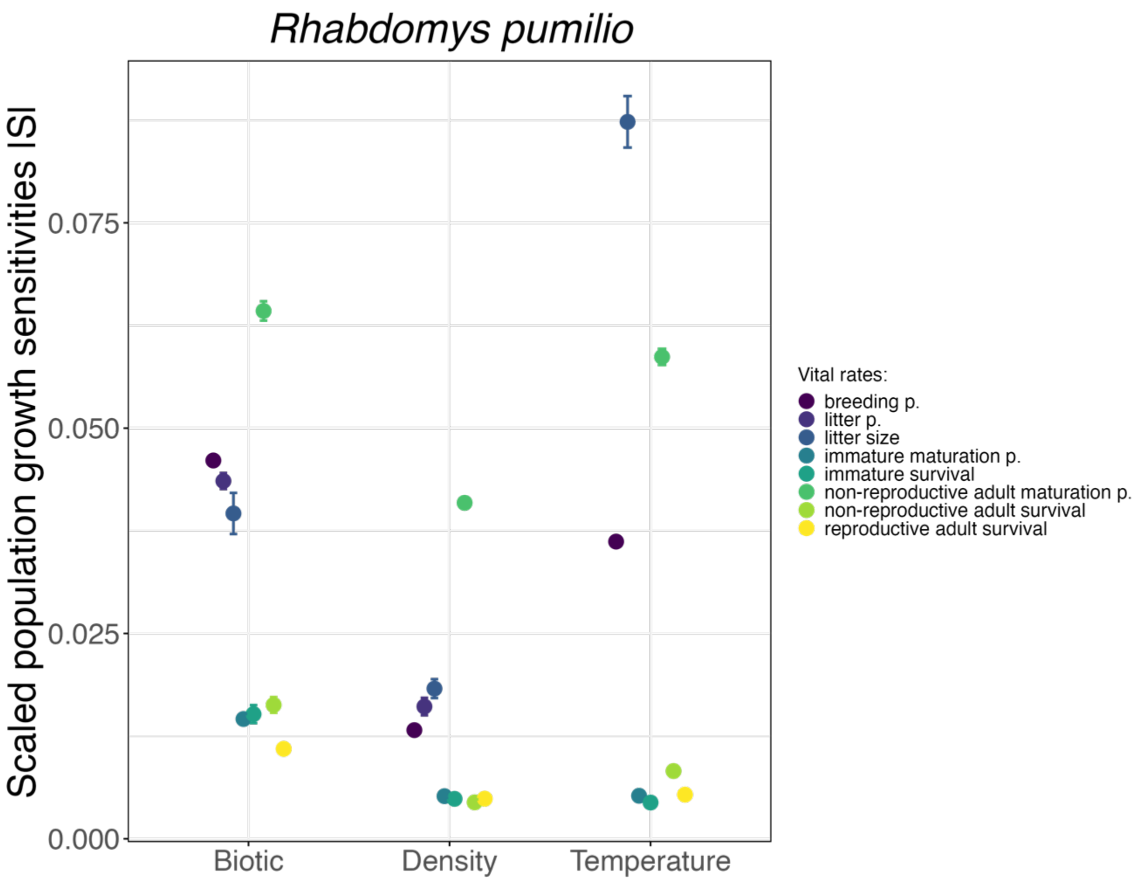

Figure S29. Scaled sensitivities of population growth rates per vital rate to different drivers for *Rhabdomys pumilio*. The dots represent the mean scaled sensitivities across the calculated resamplings per driver and vital rate combination ($n_{resamplings}$ = 100) and the error bars display the standard errors. The biotic driver is food availability, the density here refers to intraspecific density, and the climatic driver is temperature. In (12), sensitivities of λ to temperature and food (biotic here) affecting different vital rates at different densities were calculated and show that temperature and food are both important drivers of population dynamics, as are densities, and that the effects on λ largely occur via changes in reproduction and maturation (see Fig. 5 in (12)).

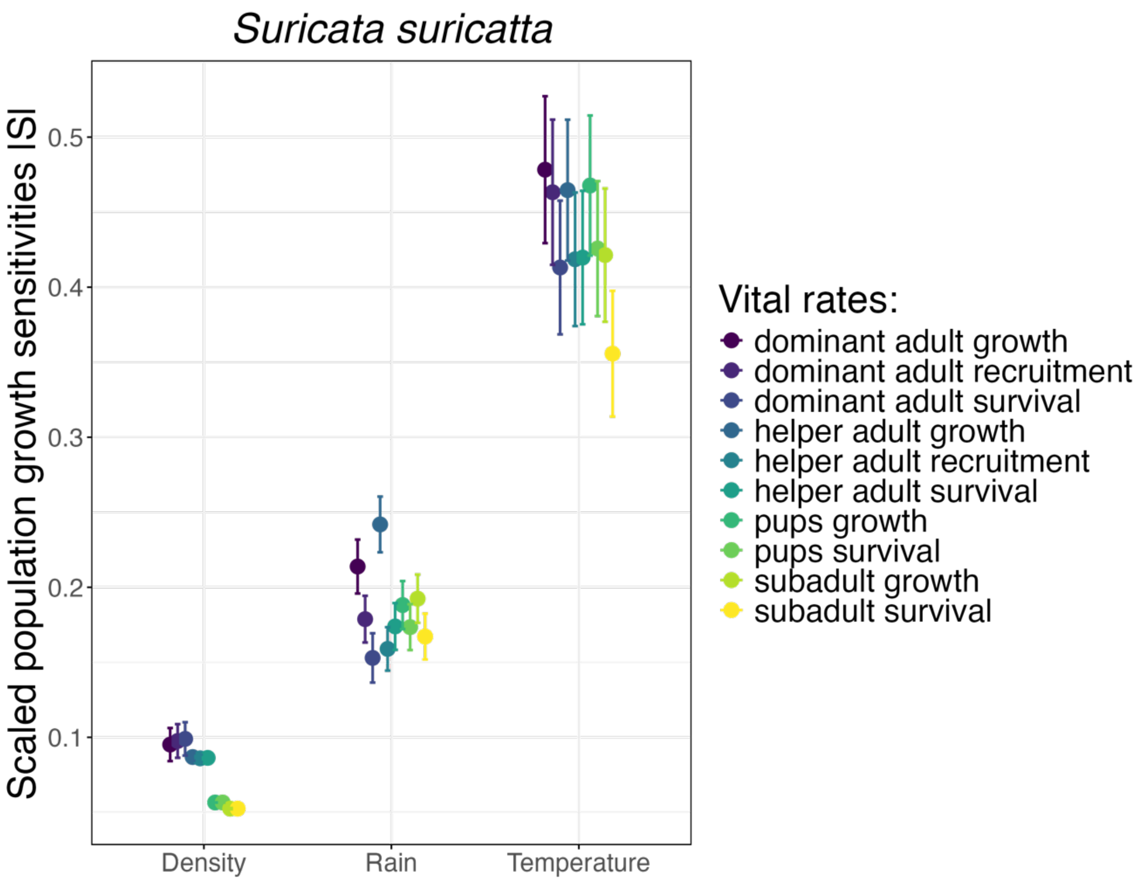

Figure S30. Scaled sensitivities of population growth rates per vital rate to different drivers for *Suricata suricatta*. The dots represent the mean scaled sensitivities across the calculated resamplings per driver and vital rate combination ($n_{resamplings} = 100$) and the error bars display the standard errors. The climatic drivers here are rain and temperature. In (5), sensitivities of λ to different life cycle stages at equilibrium densities were calculated and show that subadults and pups generally contribute little to population dynamics (see Fig. S12 in (5)).

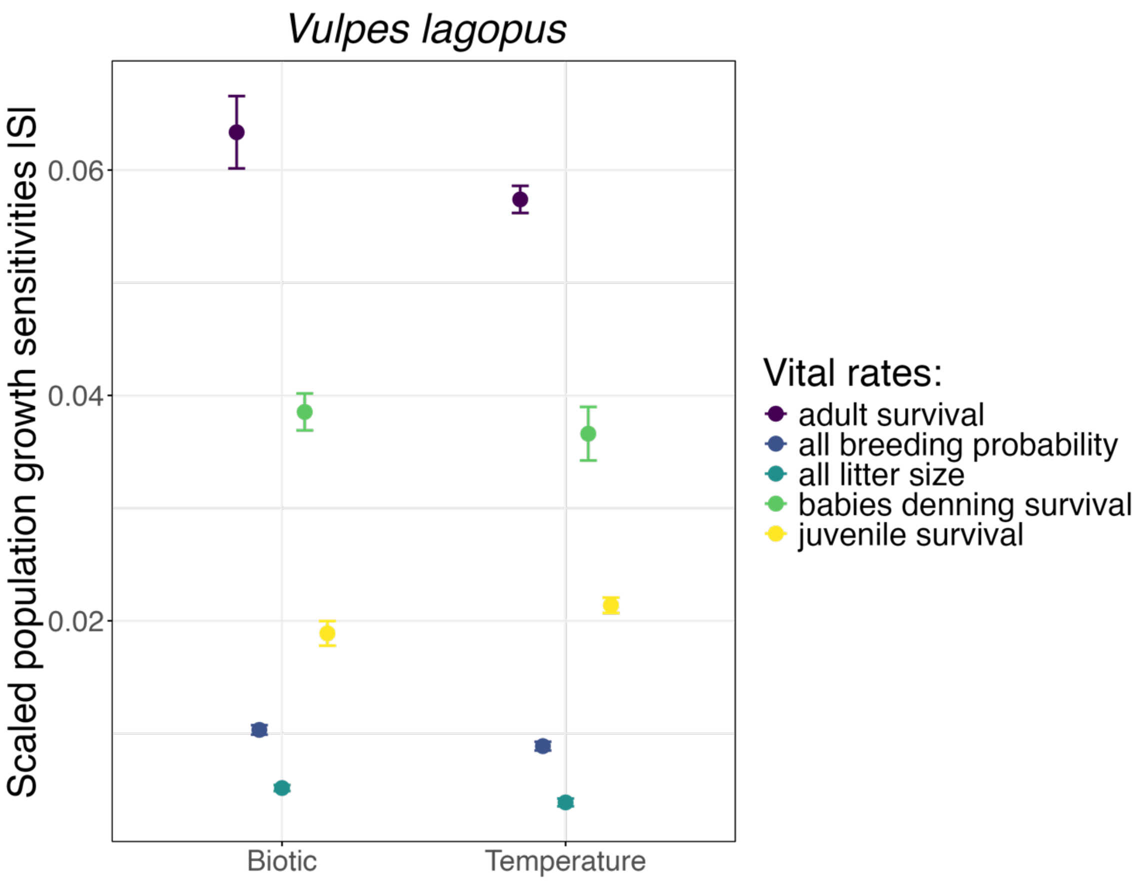

Figure S31. Scaled sensitivities of population growth rates per vital rate to different drivers for *Vulpes lagopus*. The dots represent the mean scaled sensitivities across the calculated resamplings per driver and vital rate combination ($n_{resamplings}$ = 100) and the error bars display the standard errors. The biotic driver here represents reindeer carcass availability and goose abundance, and the climatic driver is sea ice extent, also classified as sea ice extent. In (66), sensitivities of λ to different vital rates were calculated and show that annual adult survival contributes most to population dynamics (see Fig. 5 in (66)).

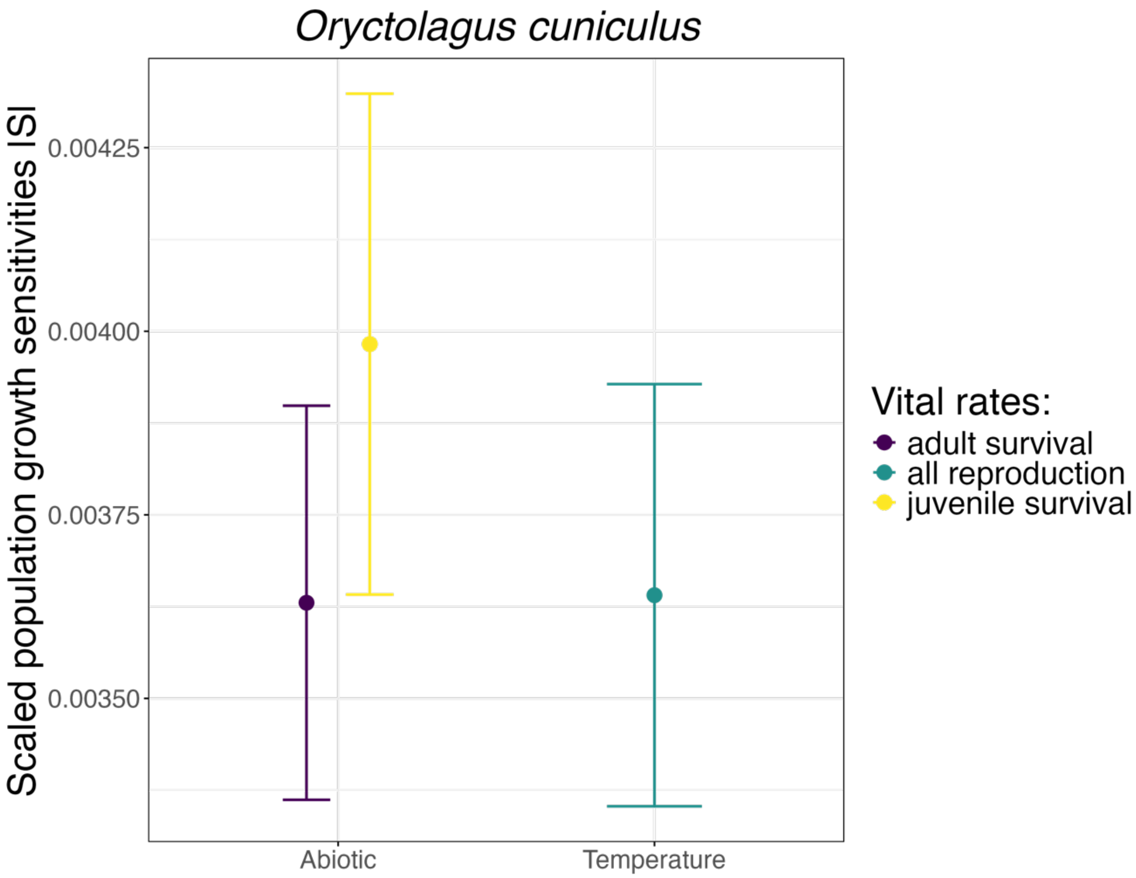

Figure S32. Scaled sensitivities of population growth rates per vital rate to different drivers for *Oryctolagus cuniculus*. The dots represent the mean scaled sensitivities across the calculated resamplings per driver and vital rate combination ($n_{resamplings}$ = 100) and the error bars display the standard errors. The biotic driver here represents reindeer carcass availability and goose abundance, and the climatic driver is sea ice extent, also classified as sea ice extent.

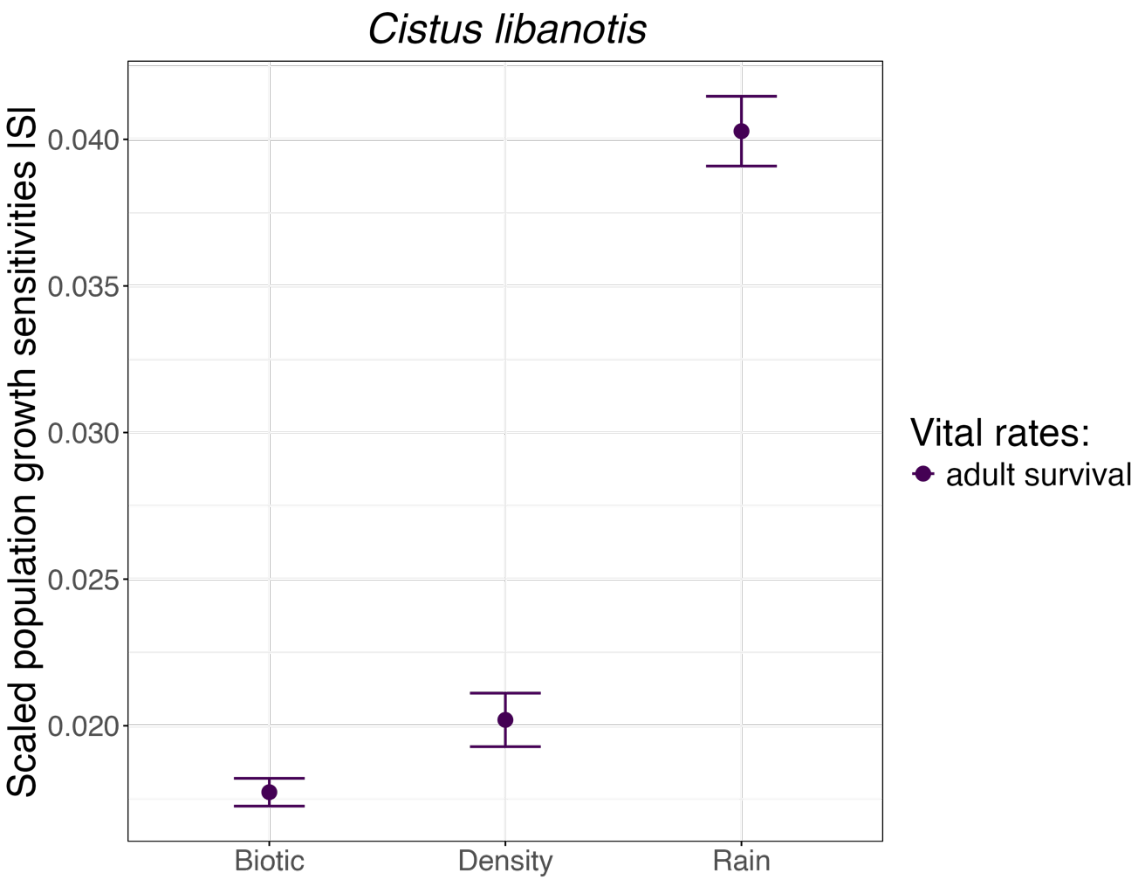

Figure S33. Scaled sensitivities of population growth rates per vital rate to different drivers for *Cistus libanotis*. The dots represent the mean scaled sensitivities across the calculated resamplings per driver and vital rate combination ($n_{resamplings}$ = 100) and the error bars display the standard errors. The biotic driver is interspecific density, the climatic driver is rain, and density represents intraspecific density. In (62), sensitivities of λ to rain, intraspecific density, and interspecific density (biotic here) affecting different vital rates at equilibrium densities were calculated and show that rain has a stronger effect on sensitivities than intraspecific densities (see Fig. 4 in (62)).

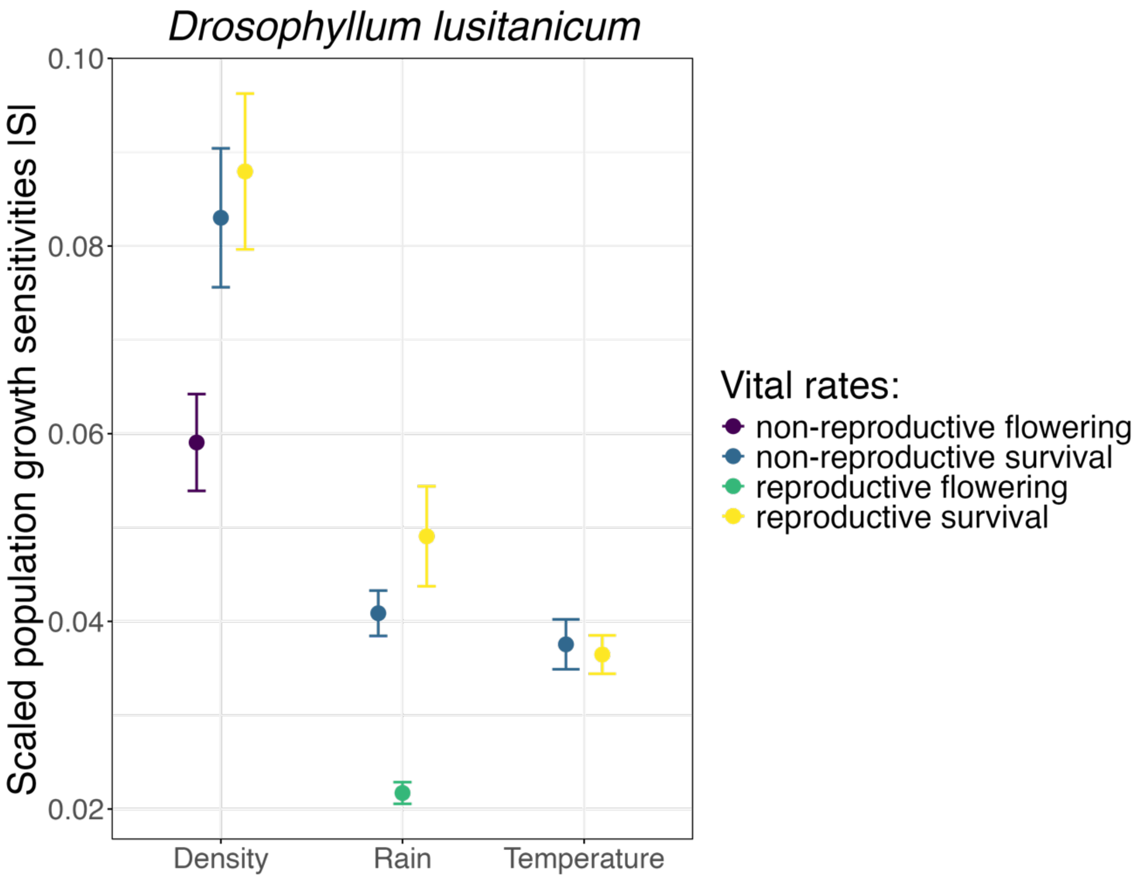

Figure S34. Scaled sensitivities of population growth rates per vital rate to different drivers for *Drosophyllum lusitanicum*. The dots represent the mean scaled sensitivities across the calculated resamplings per driver and vital rate combination ($n_{resamplings}$ = 100) and the error bars display the standard errors. The climatic drivers are rain and temperature, and density represents intraspecific density.

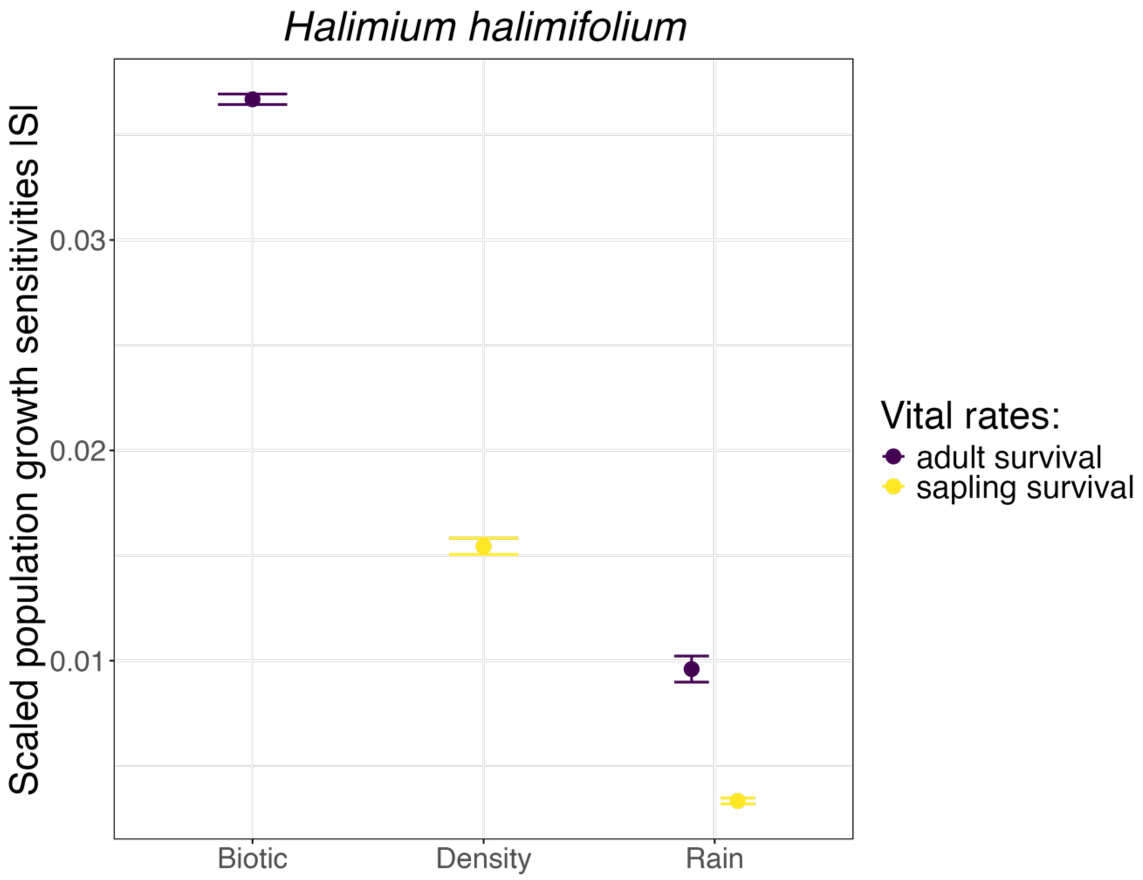

Figure S35. Scaled sensitivities of population growth rates per vital rate to different drivers for *Halimium halimifolium*. The dots represent the mean scaled sensitivities across the calculated resamplings per driver and vital rate combination ($n_{resamplings}$ = 100) and the error bars display the standard errors. The biotic driver is interspecific density, the climatic driver is rain, and density represents intraspecific density. In (62), sensitivities of λ to rain, intraspecific density, and interspecific density (biotic here) affecting different vital rates at equilibrium densities were calculated and show that changes in interspecific densities in adult survival have the strongest effect on sensitivities (see Fig. 4 in (62)).

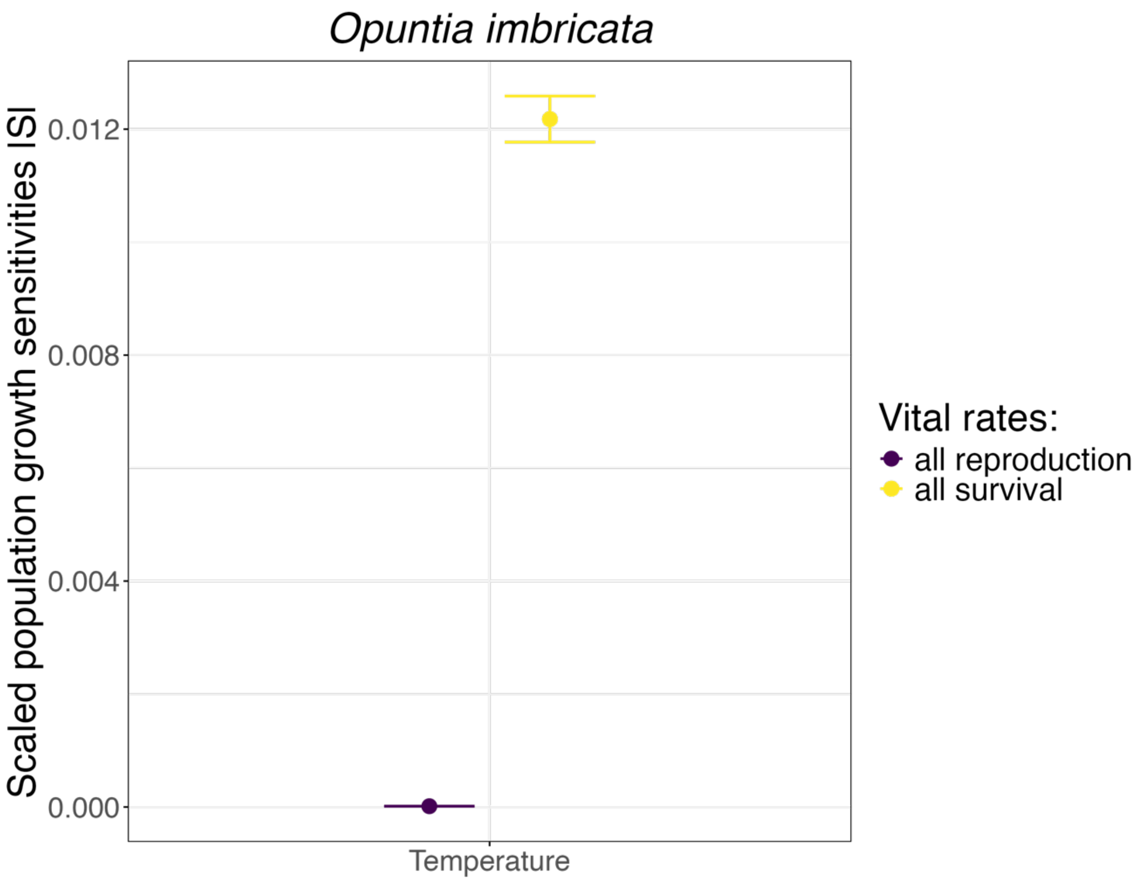

Figure S36. Scaled sensitivities of population growth rates per vital rate to different drivers for *Opuntia imbricata*. The dots represent the mean scaled sensitivities across the calculated resamplings per driver and vital rate combination ($n_{resamplings}$ = 100) and the error bars display the standard errors. The climatic driver temperature represents mean average daily temperatures of two different time windows and mean minimum daily temperature.

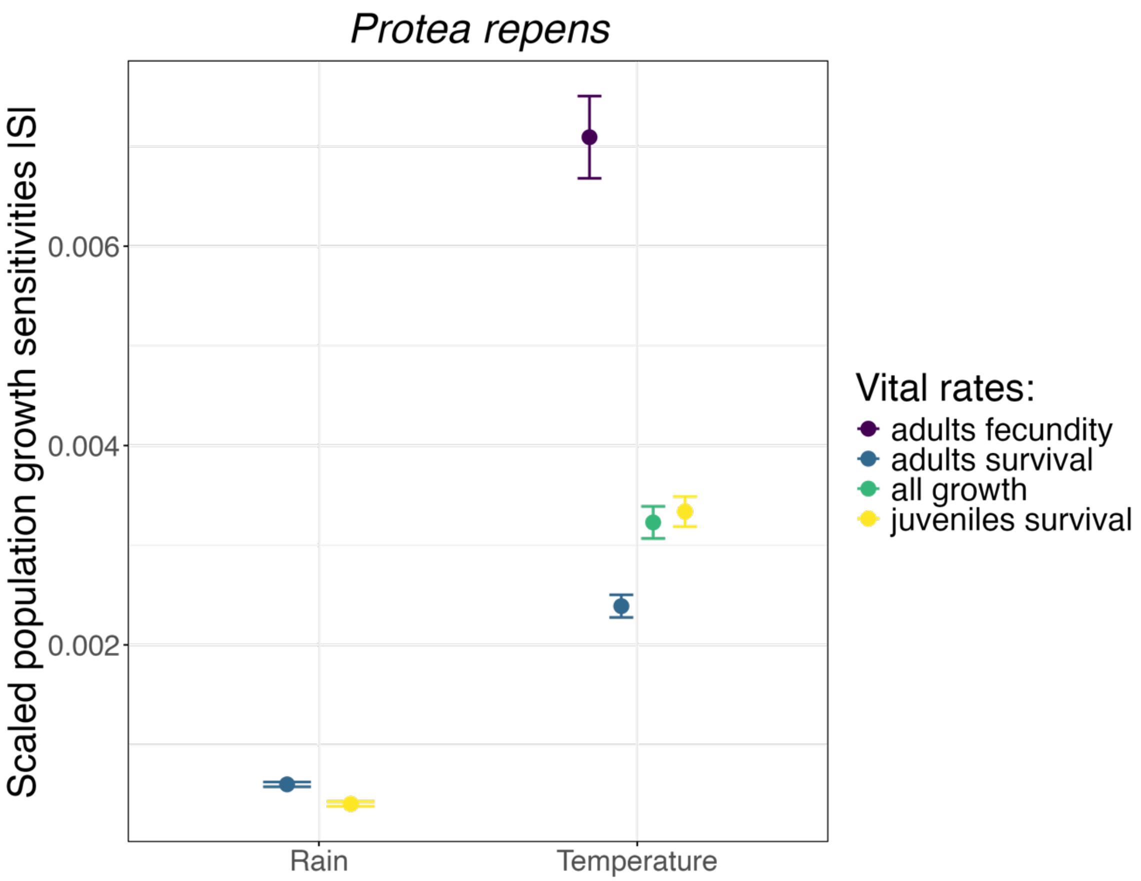

Figure S37. Scaled sensitivities of population growth rates per vital rate to different drivers for *Protea repens*. The dots represent the mean scaled sensitivities across the calculated resamplings per driver and vital rate combination ($n_{resamplings}$ = 100) and the error bars display the standard errors. The climatic drivers are rain and temperature.

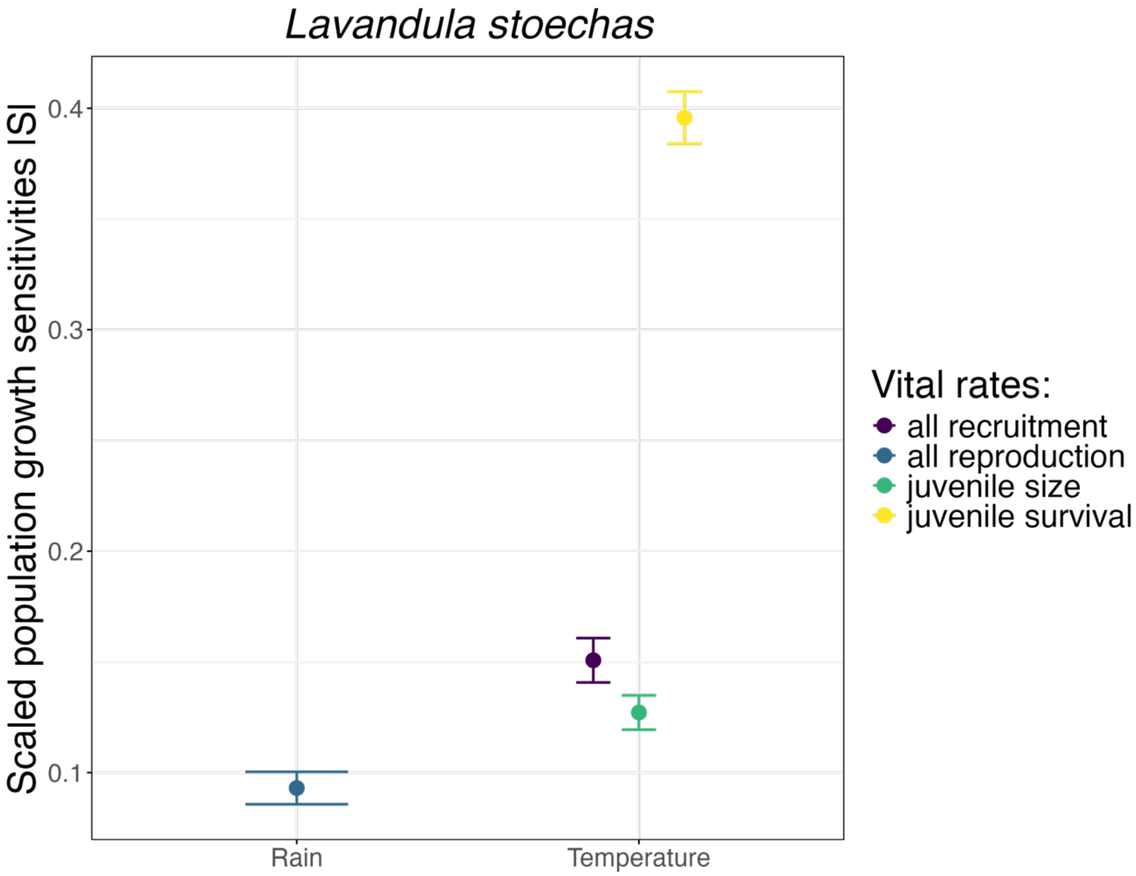

Figure S38. Scaled sensitivities of population growth rates per vital rate to different drivers for *Lavandula stoechas*. The dots represent the mean scaled sensitivities across the calculated resamplings per driver and vital rate combination ($n_{resamplings}$ = 100) and the error bars display the standard errors. The climatic drivers are rain and temperature.

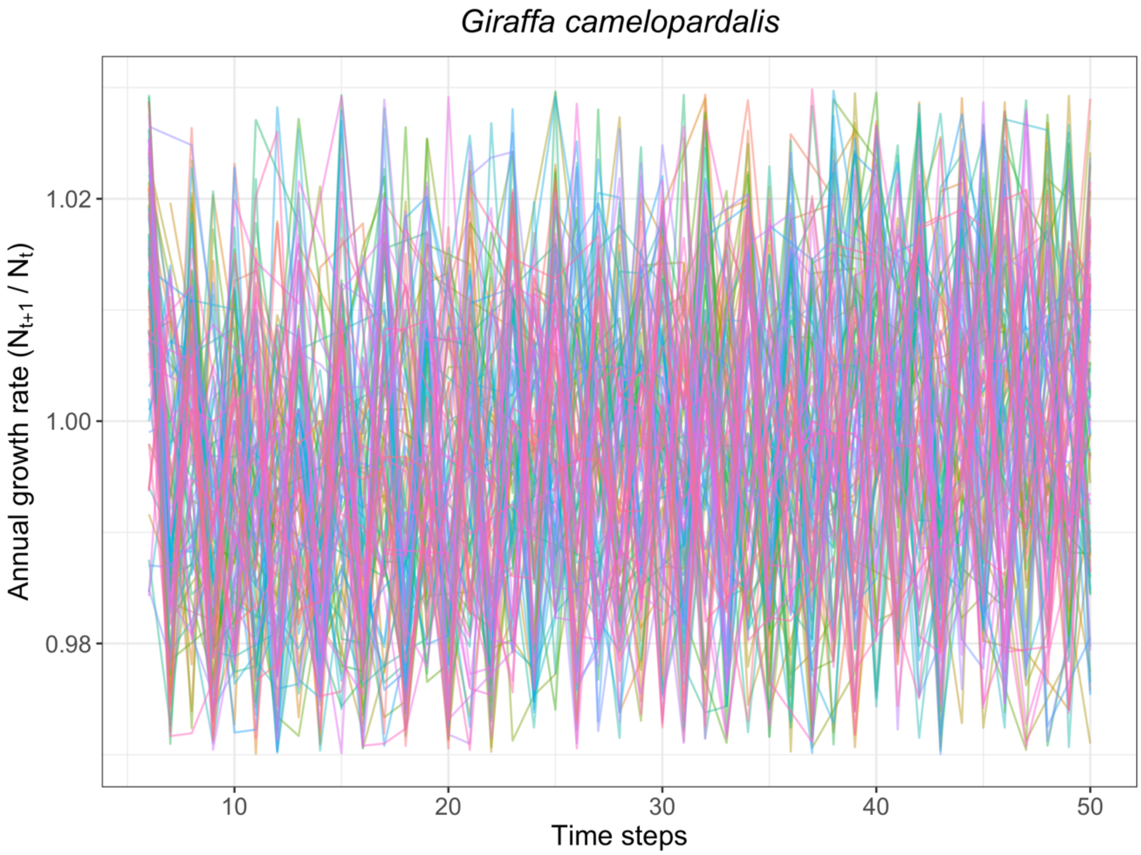

Figure S39. Time series of simulated annual growth rates (N_{t+1}/N_t) for *Giraffa camelopardalis*, which we averaged to calculate λ (after discarding transient dynamics). The colors represent the multiple simulations (n = 100).

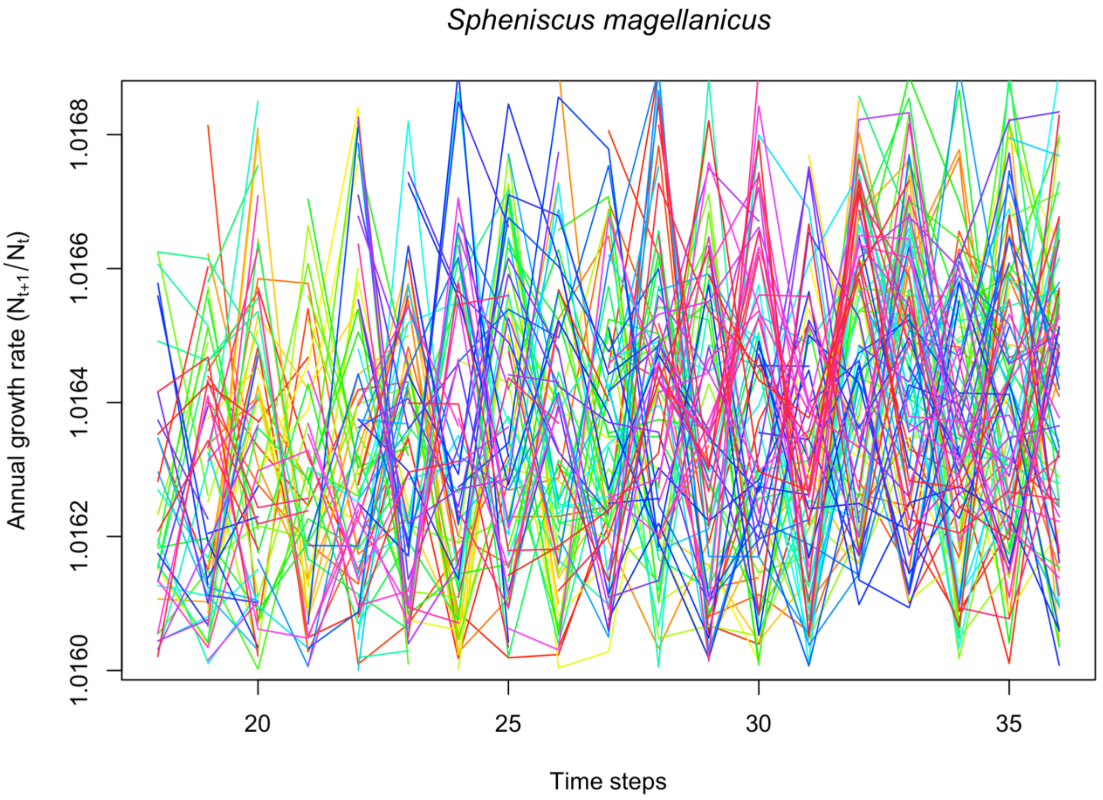

Figure S40. Time series of simulated annual growth rates (N_{t+1}/N_t) for *Spheniscus magellanicus*, which we averaged to calculate λ (after discarding transient dynamics). The colors represent the multiple simulations (n = 100).

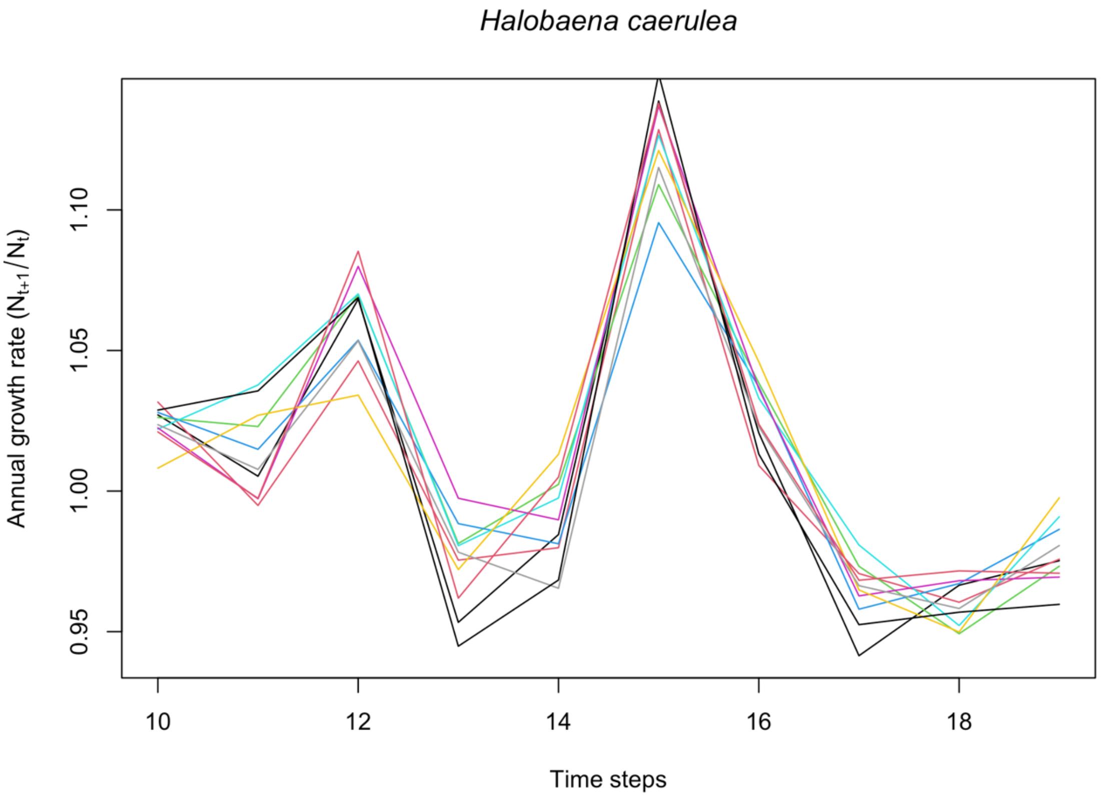

Figure S41. Time series of simulated annual growth rates (N_{t+1}/N_t) for *Halobaena caerulea*, which we averaged to calculate λ (after discarding transient dynamics). The colors represent the multiple simulations (n = 10).

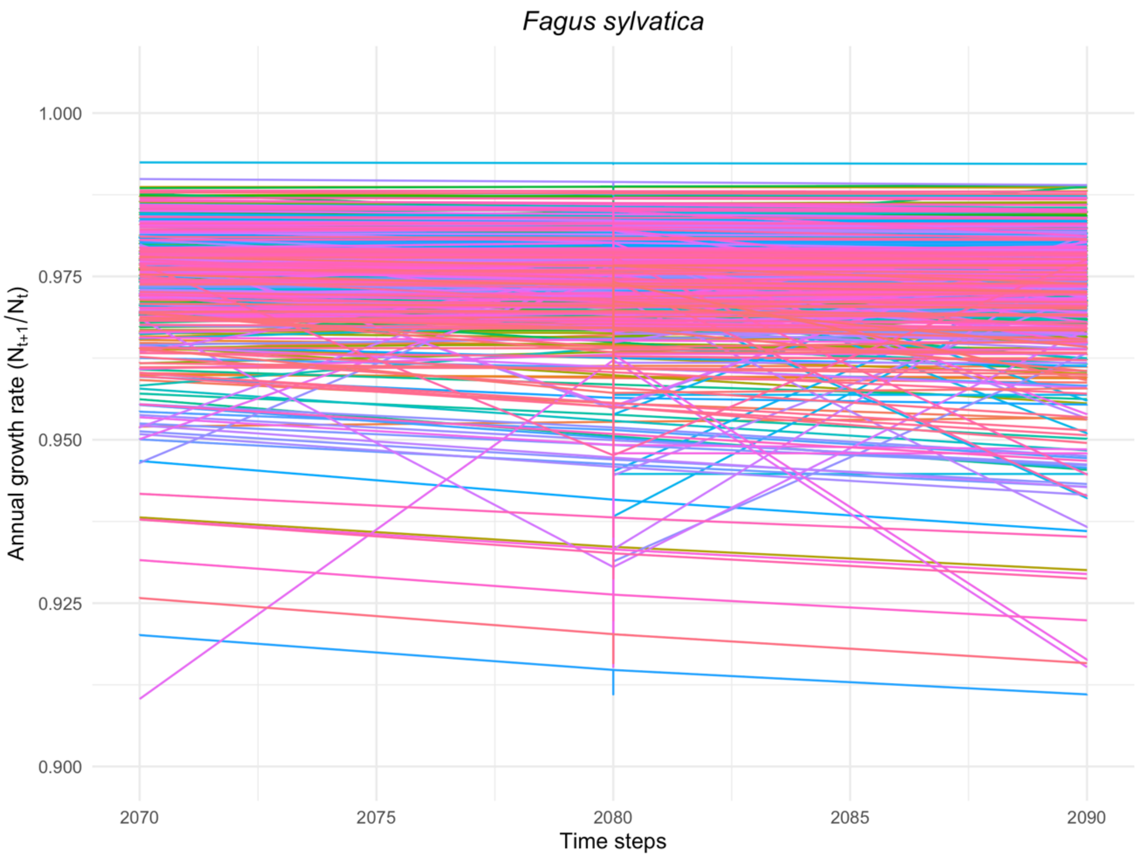

Figure S42. Time series of simulated annual growth rates (N_{t+1}/N_t) for *Fagus sylvatica*, which we averaged to calculate λ (after discarding transient dynamics). The colors represent the multiple simulations and sites (see species-specific details in SI). The variation in λ is largely attributed to variation among sites as we ensured to remove sites from analyses where λ values changed direction in simulations.

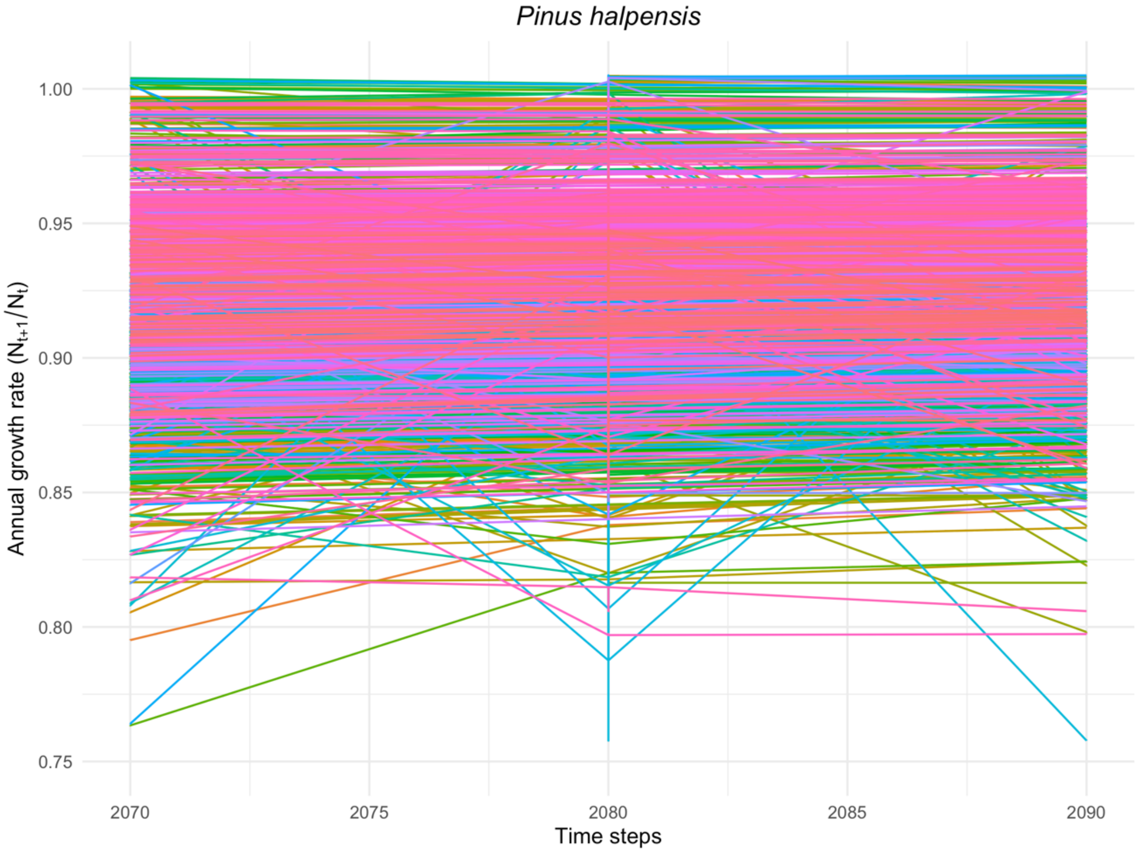

Figure S43. Time series of simulated annual growth rates (N_{t+1}/N_t) for *Pinus halepensis*, which we averaged to calculate λ (after discarding transient dynamics). The colors represent the multiple simulations and sites (see species-specific details in SI). The variation in λ is largely attributed to variation among sites as we ensured to remove sites from analyses where λ values changed direction in simulations.

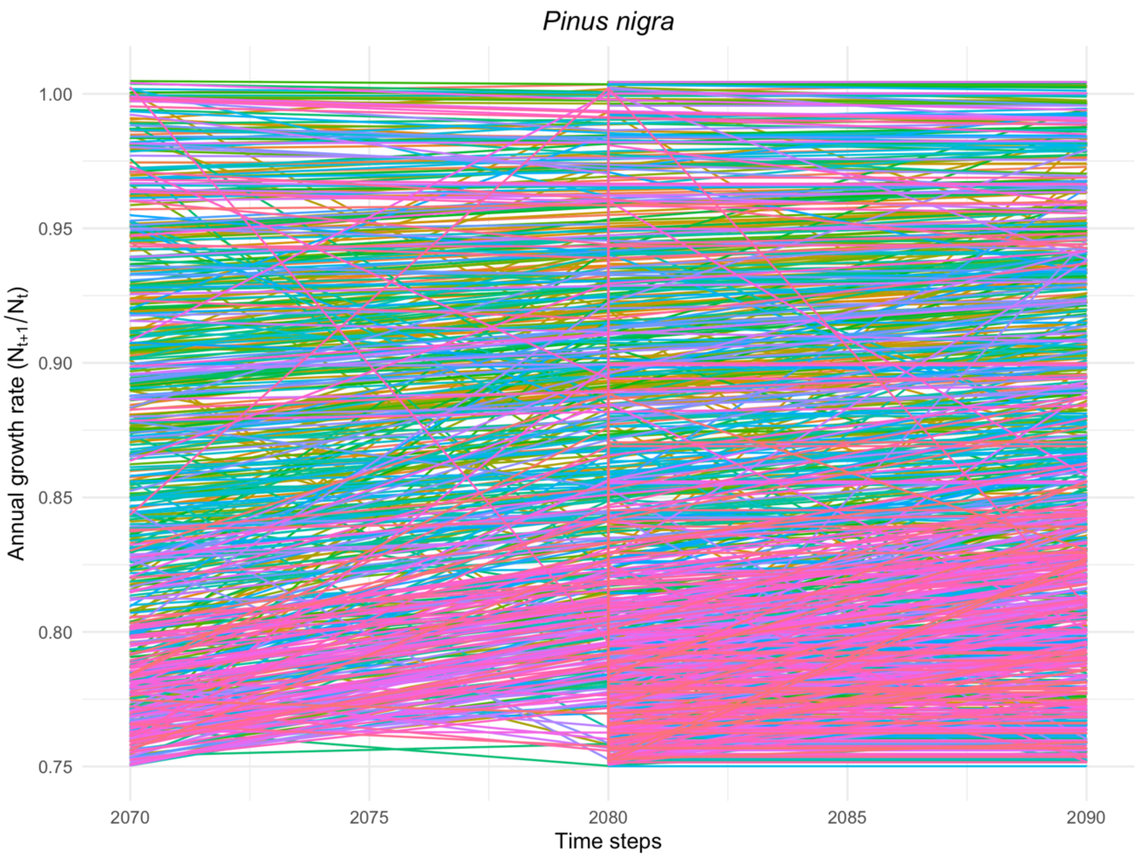

Figure S44. Time series of simulated annual growth rates (N_{t+1}/N_t) for *Pinus nigra*, which we averaged to calculate λ (after discarding transient dynamics). The colors represent the multiple simulations and sites (see species-specific details in SI). The variation in λ is largely attributed to variation among sites as we ensured to remove sites from analyses where λ values changed direction in simulations.

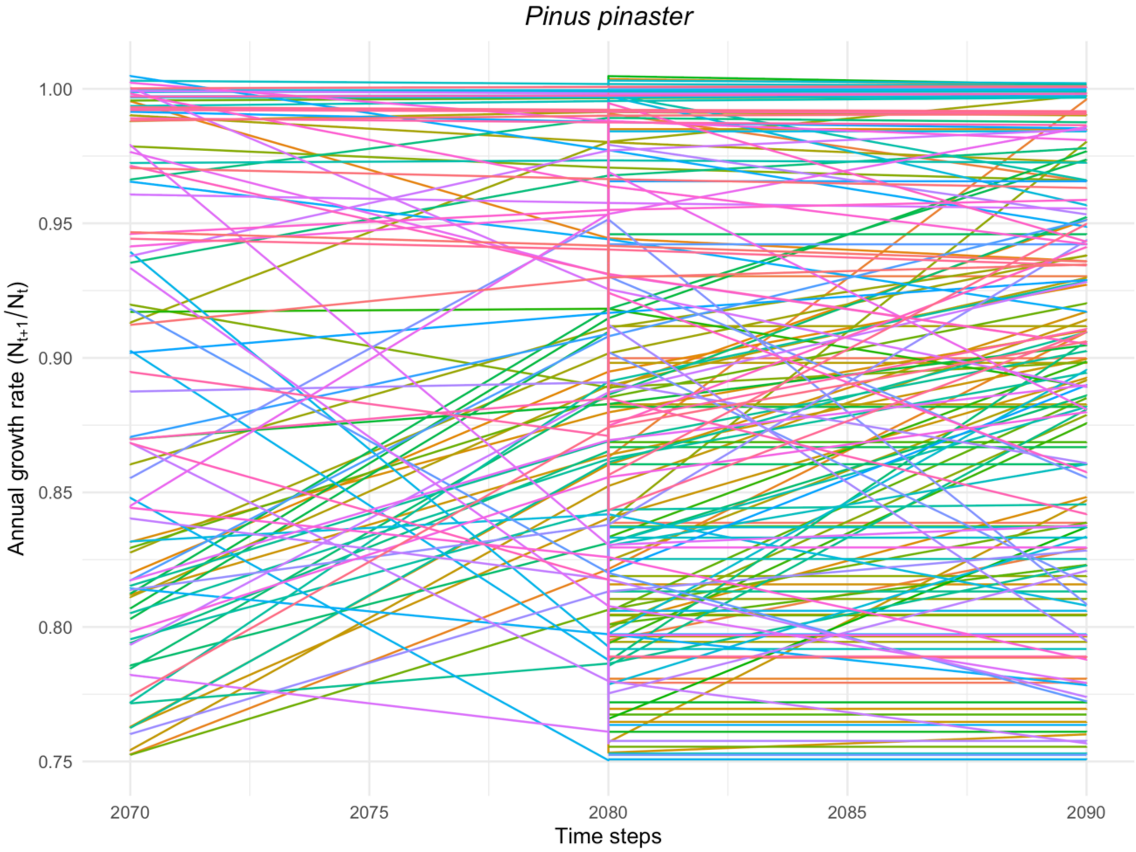

Figure S45. Time series of simulated annual growth rates (N_{t+1}/N_t) for *Pinus pinaster*, which we averaged to calculate λ (after discarding transient dynamics). The colors represent the multiple simulations and sites (see species-specific details in SI). The variation in λ is largely attributed to variation among sites as we ensured to remove sites from analyses where λ values changed direction in simulations.

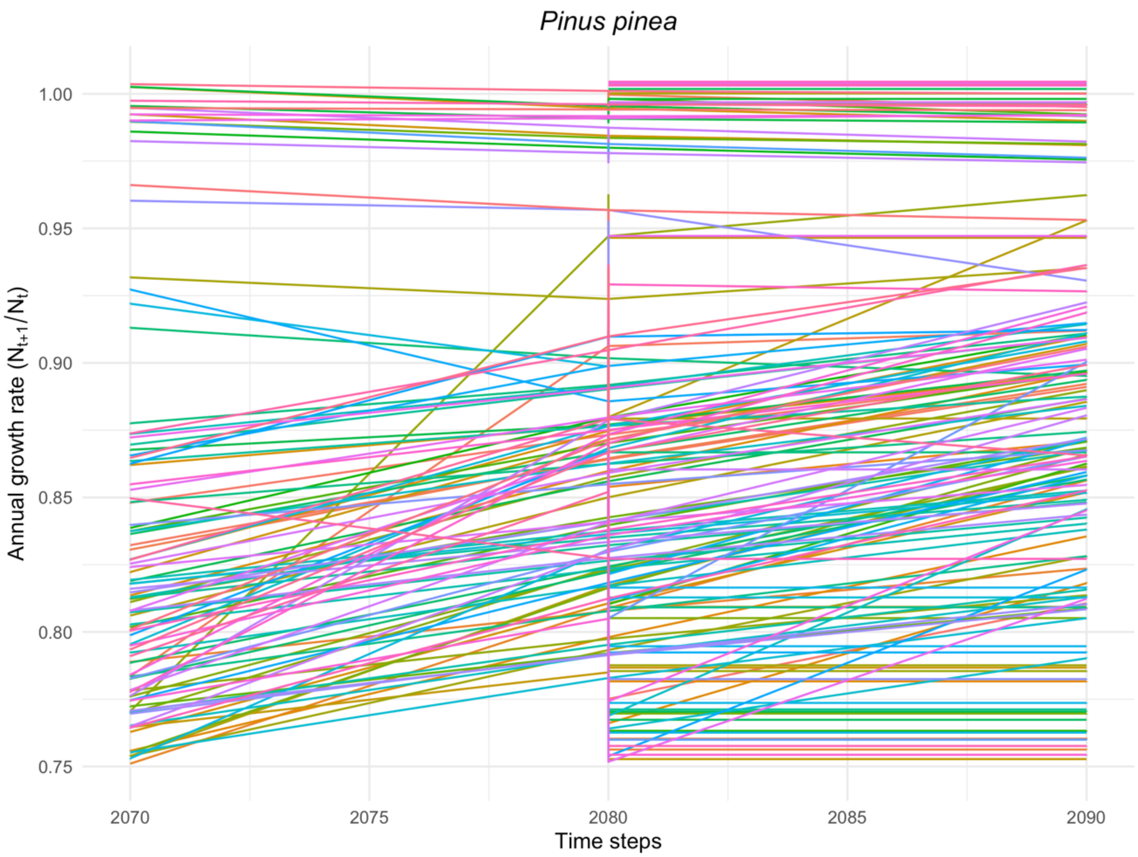

Figure S46. Time series of simulated annual growth rates (N_{t+1}/N_t) for *Pinus pinea*, which we averaged to calculate λ (after discarding transient dynamics). The colors represent the multiple simulations and sites (see species-specific details in SI). The variation in λ is largely attributed to variation among sites as we ensured to remove sites from analyses where λ values changed direction in simulations.

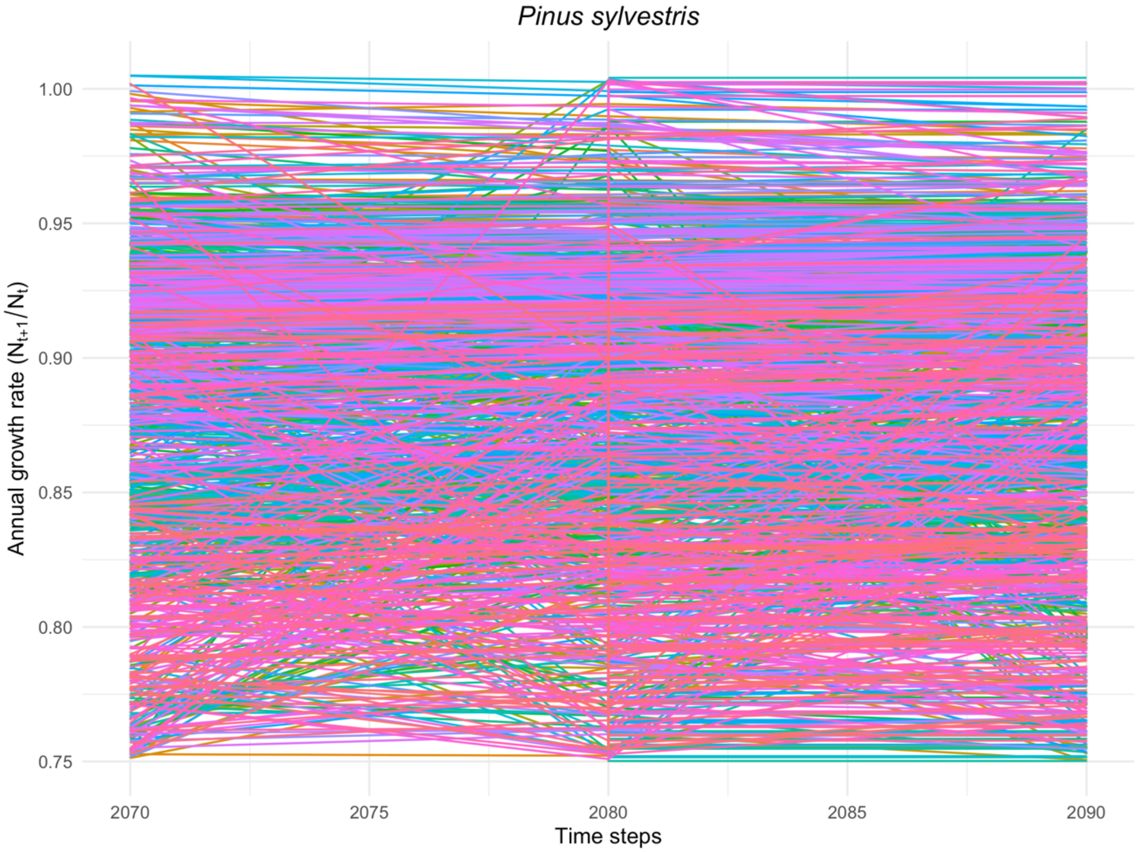

Figure S47. Time series of simulated annual growth rates (N_{t+1}/N_t) for *Pinus sylvestris*, which we averaged to calculate λ (after discarding transient dynamics). The colors represent the multiple simulations and sites (see species-specific details in SI). The variation in λ is largely attributed to variation among sites as we ensured to remove sites from analyses where λ values changed direction in simulations.

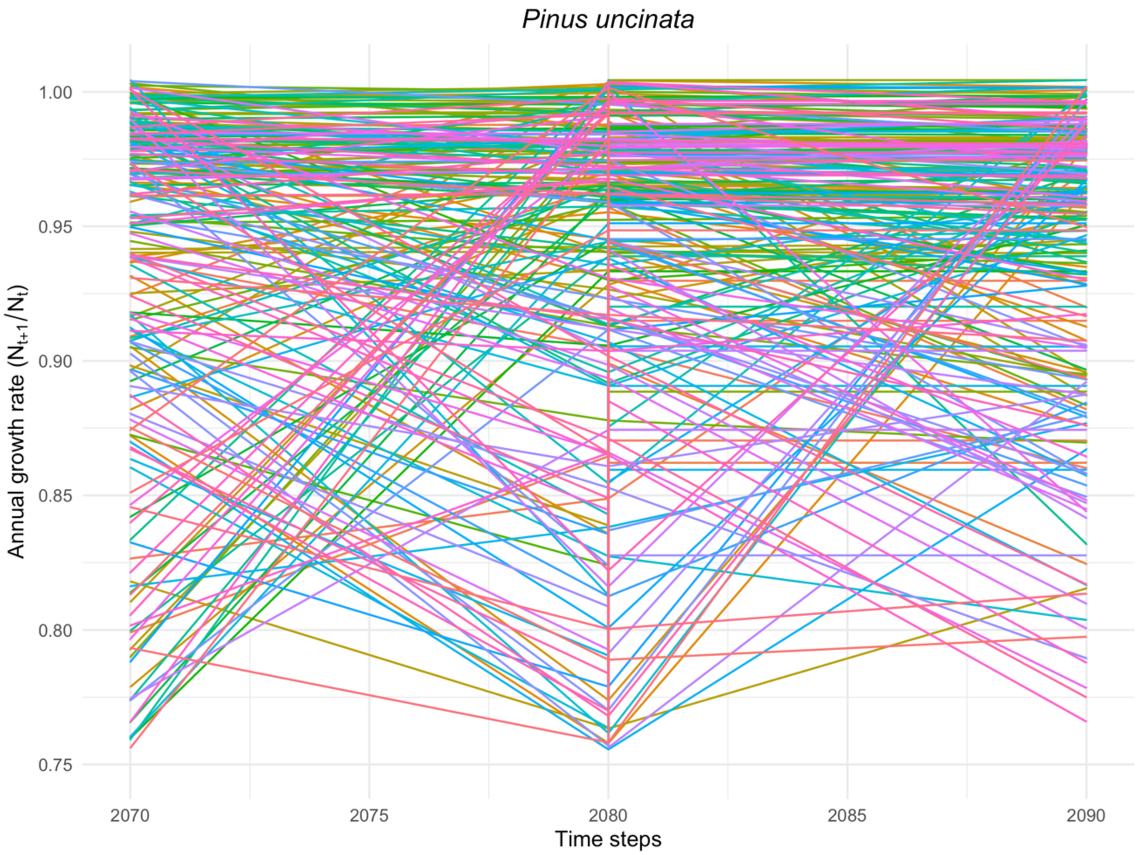

Figure S48. Time series of simulated annual growth rates (N_{t+1}/N_t) for *Pinus uncinata*, which we averaged to calculate λ (after discarding transient dynamics). The colors represent the multiple simulations and sites (see species-specific details in SI). The variation in λ is largely attributed to variation among sites as we ensured to remove sites from analyses where λ values changed direction in simulations.

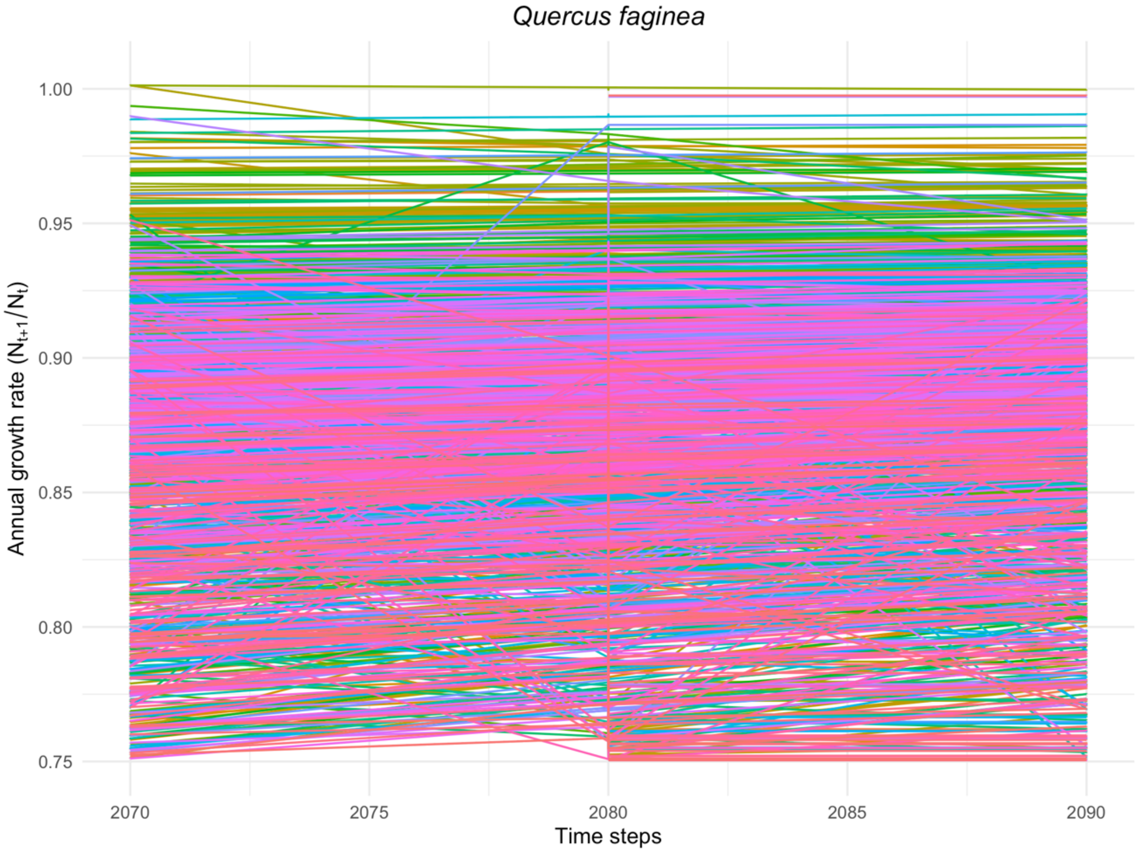

Figure S49. Time series of simulated annual growth rates (N_{t+1}/N_t) for *Quercus faginea*, which we averaged to calculate λ (after discarding transient dynamics). The colors represent the multiple simulations and sites (see species-specific details in SI). The variation in λ is largely attributed to variation among sites as we ensured to remove sites from analyses where λ values changed direction in simulations.

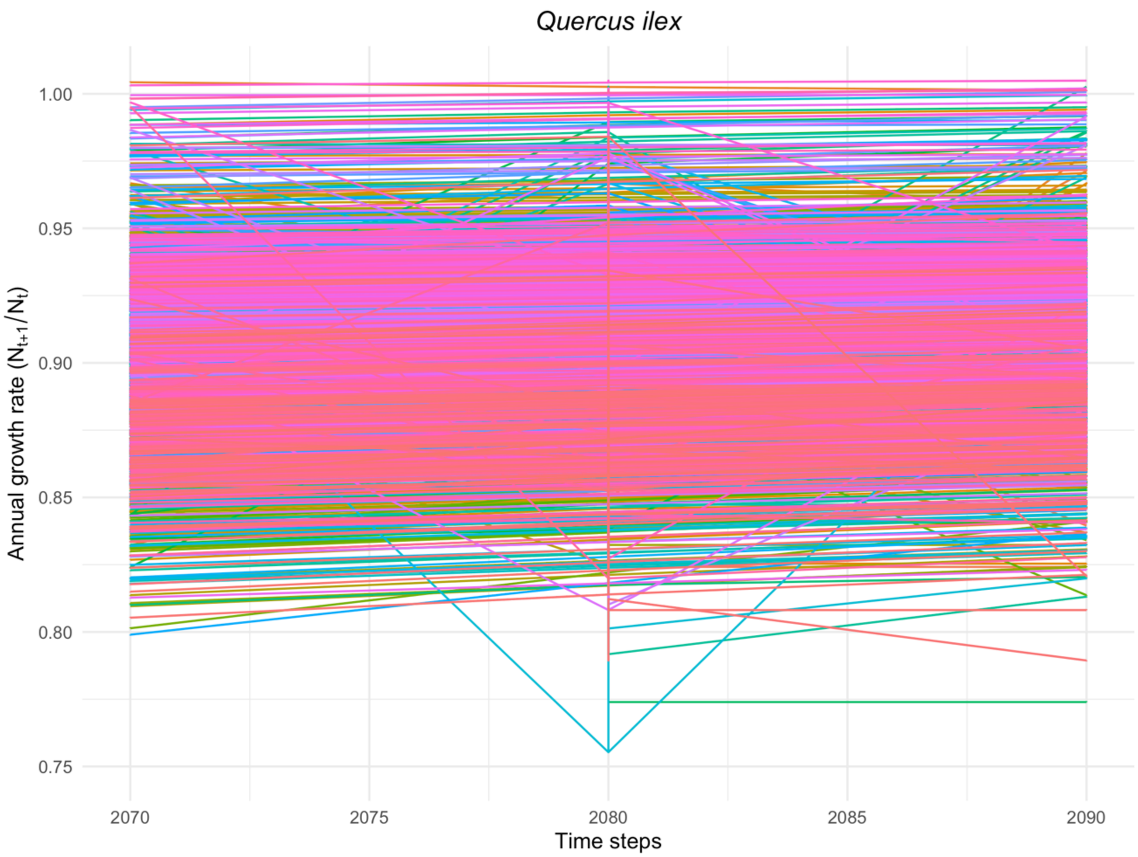

Figure S50. Time series of simulated annual growth rates (N_{t+1}/N_t) for *Quercus ilex*, which we averaged to calculate λ (after discarding transient dynamics). The colors represent the multiple simulations and sites (see species-specific details in SI). The variation in λ is largely attributed to variation among sites as we ensured to remove sites from analyses where λ values changed direction in simulations.

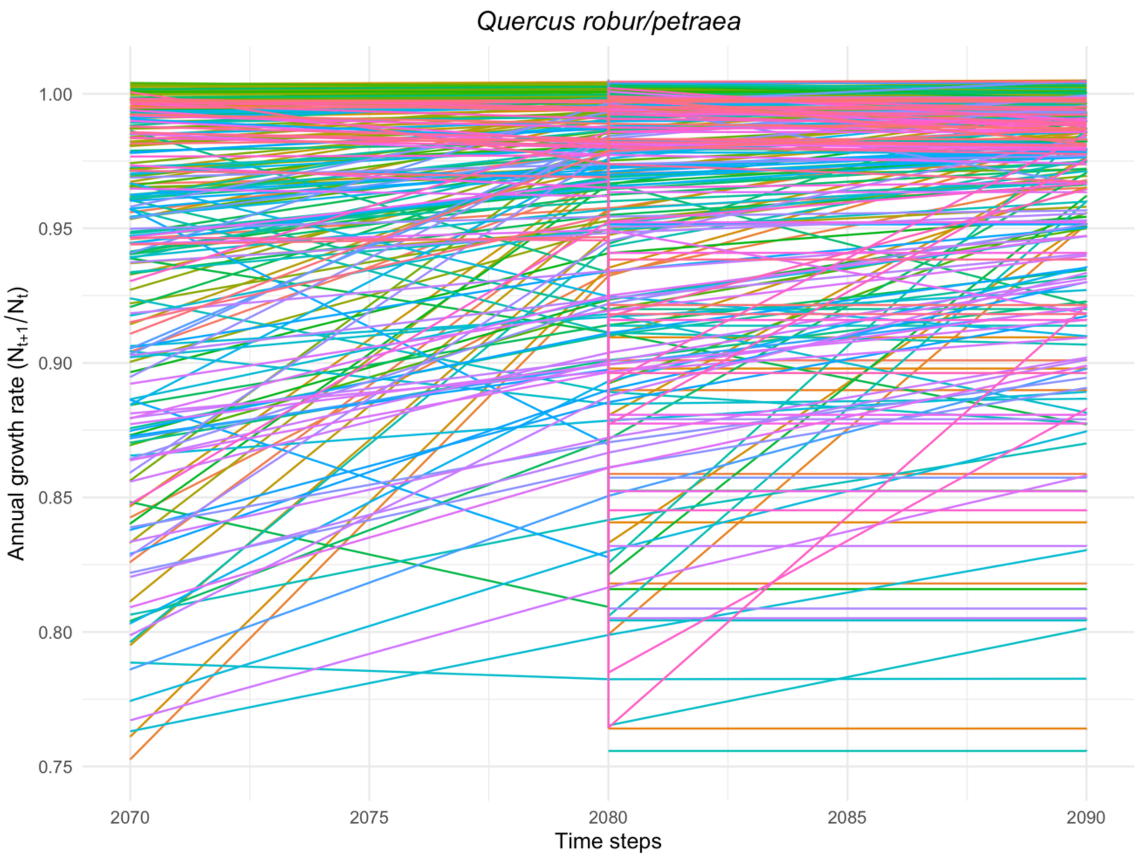

Figure S51. Time series of simulated annual growth rates (N_{t+1}/N_t) for *Quercus robur/petraea*, which we averaged to calculate λ (after discarding transient dynamics) The colors represent the multiple simulations and sites (see species-specific details in SI). The variation in λ is largely attributed to variation among sites as we ensured to remove sites from analyses where λ values changed direction in simulations.

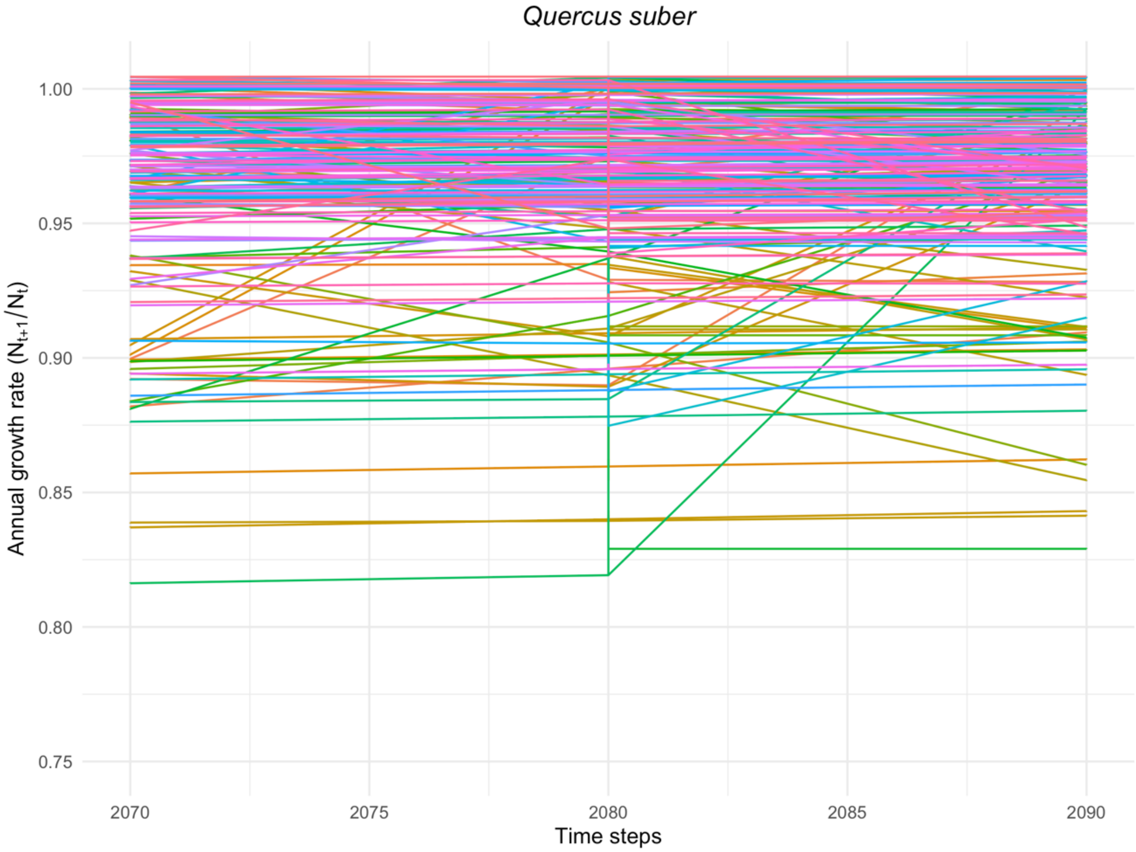

Figure S52. Time series of simulated annual growth rates (N_{t+1}/N_t) for *Quercus suber,* which we averaged to calculate λ (after discarding transient dynamics). The colors represent the multiple simulations and sites (see species-specific details in SI). The variation in λ is largely attributed to variation among sites as we ensured to remove sites from analyses where λ values changed direction in simulations.

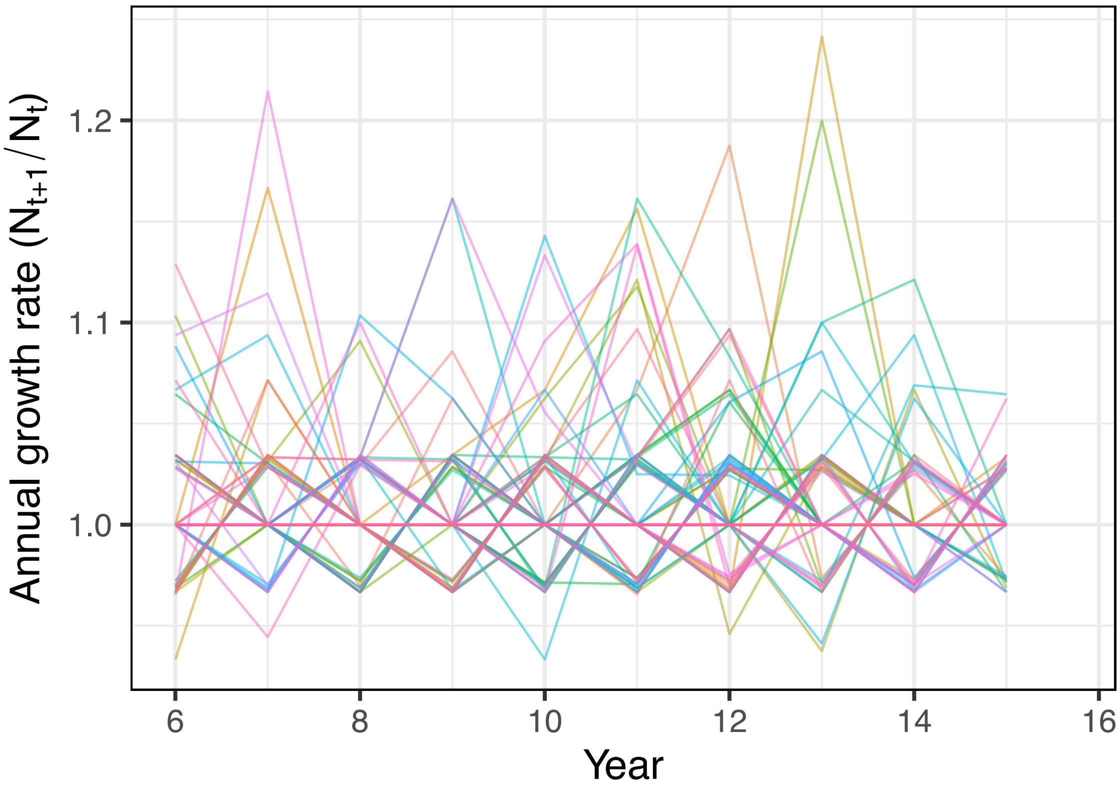

Figure S53. Time series of simulated annual growth rates (N_{t+1}/N_t) for *Oryctolagus cuniculus,* which we averaged to calculate λ (after discarding transient dynamics of year 1-5). The colors represent the multiple simulations and sites (see species-specific details in SI). The variation in λ is largely attributed to variation among sites as we ensured to remove sites from analyses where λ values changed direction in simulations. The colors represent the multiple simulations (n = 100).
